# Quantitative analysis of mouse corpus callosum from electron microscopy images

**DOI:** 10.1016/j.dib.2015.08.022

**Published:** 2015-09-03

**Authors:** Kathryn L. West, Nathaniel D. Kelm, Robert P. Carson, Mark D. Does

**Affiliations:** a Department of Biomedical Engineering, Vanderbilt University, United States; b Vanderbilt University Institute of Imaging Science, Vanderbilt University, United States; c Department of Pediatric Neurology, United States; d Department of Radiology and Radiological Sciences, Vanderbilt University School of Medicine, United States; e Department of Electrical Engineering, Vanderbilt University, United States

**Keywords:** G ratio, MRI, Histology, Hypomyelinated, Tuberous sclerosis

## Abstract

This article provides morphometric analysis of 72 electron microscopy images from control (*n*=4) and hypomyelinated (*n*=2) mouse corpus callosum. Measures of axon diameter and g-ratio were tabulated across all brains from two regions of the corpus callosum and a non-linear relationship between axon diameter and g-ratio was observed. These data are related to the accompanying research article comparing multiple methods of measuring g-ratio entitled ‘A revised model for estimating g-ratio from MRI’ (West et al., *NeuroImage*, 2015).

**Specifications Table**

**Table:** 

Subject area	*Neuroanatomy*
More specific subject area	*Morphometry*
Type of data	*Electron Microscopy Images (.tif), data figures*
How data was acquired	*TEM, Philips/FEI Tecnai T12 electron microscope*
Data format	*Raw*
Experimental factors	*Tissue was perfusion and immersion fixed with 2.5% glutaraldehyde+2% paraformaldehyde with 1* *mM Gadolinium (Gd) in PBS followed by washing in 1X PBS with 1* *mM Gd. Samples were stained with 1% toluidine blue, 2% uranyl acetate (aqueous), and lead citrate.*
Experimental features	*EM images were analyzed using MATLAB with a semi-automatic method for segmentation and morphometric analysis*
Data source location	*Nashville, TN*
Data accessibility	*Data is provided in this article*

**Value of the data**

• There are few resources for morphometric mouse histology and with a larger number of high resolution images provided, researchers can test their own hypotheses.
• Our work investigates the average values of axon diameter and g-ratio in normal and hypomyelinated mouse corpus callosum.
• We observe a non-linear relationship between axon diameter and g-ratio.

## 1 Data, experimental design, materials and methods

Animal studies were approved by the Vanderbilt University Institutional Animal Care and Use Committee. Histology was acquired from control and *Rictor* conditional knockout (CKO) mice, similar to a previously described mouse model of tuberous sclerosis complex [4]. Six adult mice were anesthetized with isoflurane and sacrificed via transcardial perfusion of 1X phosphate-buffered saline (PBS) wash followed by 2.5% glutaraldehyde+2% paraformaldehyde in PBS (modified Karnovsky solution). Following perfusion, brains were quickly removed from the skull and immersed in the fixative solution for 1 week. For MRI studies not presented here, the perfusion and immersion solutions included a paramagnetic MRI contrast agent and the fixative was washed out of brains prior to imaging and subsequent histology. For histologic preparation, a 1–2 mm sagittal slice of tissue was cut from the left hemisphere beginning at the mid-brain from each of 6 brains (*n*=4 control and *n*=2 CKO). Subsequently, 2 regions of white matter from the corpus callosum (genu- GCC and midbody- MidCC) were cut from each slice. Tissue samples were then processed for transmission electron microscopy in the Vanderbilt Cell Imaging Shared Resource-Research Electron Microscopy facility. Thick sections (0.5–1 µm) were collected using a Leica Ultracut microtome (UC-7), then stained with 1% toluidine blue. Ultra-thin sections (70–80 nm) were then cut and collected on 300-mesh copper grids. Copper grids were post-section stained at room temperature with 2% uranyl acetate (aqueous) for 15 min and then with lead citrate for 10 min. Ultra-thin sections were imaged on the Philips/FEI Tecnai T12 electron microscope at 15,000×magnification. From each section, six images were acquired using a side-mounted AMT CCD camera, resulting in a total of 6 mice × 2 regions× 6 images/region/mouse=72 images.

**Fig. 1:**
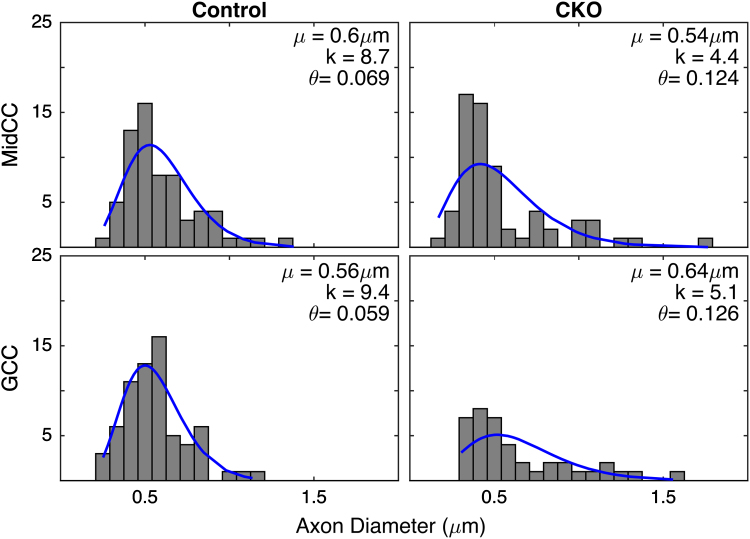
Representative axon diameter histograms with *γ*-distribution fits (blue line) from MidCC (top) and GCC (bottom) regions of the corpus callosum of control (left) and *Rictor* CKO (right) mice. In each frame, the fitted distribution parameters, characteristic shape (*k*) and scale (*θ* ), and mean diameter (*µ*) are also shown.

The pipeline of histology analysis is summarized in the attached manuscript (West et al., 2015). Images were segmented using the histogram of pixel gray scale values, defining the threshold between myelin and non-myelin pixels at the nadir. This provided a binary image where myelin=1 and non-myelin=0 (Fig. 1c), and an estimate of *MVF*. From the binary image, each myelinated axon was manually identified and its area (*A*_A*i*_, for the *i*th axon) was computed using a region growing algorithm. This value provided an estimate of axon radius $r_{i}=\sqrt{A_{Ai}/\pi }$, and the sum of all axon areas provided an estimate of *AVF*. For each axon, the thickness of the surrounding myelin (*∆*_*i*_) was calculated as the average of manual measurements made in two locations, and the g-ratio was estimated as $g_{i}=r_{i}/\left(r_{i}+\Delta_{i}\right)$.

**Fig. 2:**
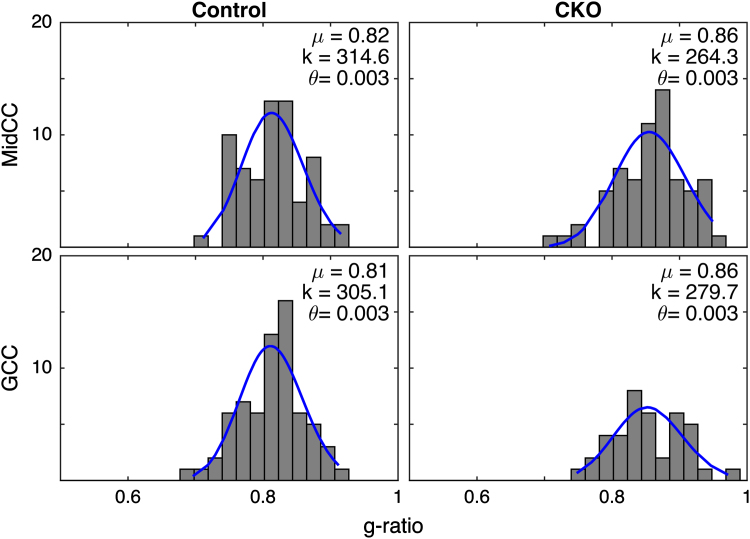
Representative g-ratio histograms with *γ*-distribution (blue line) fits from MidCC (top) and GCC (bottom) regions of the corpus callosum of control (left) and *Rictor* CKO (right) mice. In each frame, the fitted distribution parameters, characteristic shape (*k*) and scale (*θ*), and mean g-ratio (*µ*) are also shown.

Fig. 1 displays histograms of axon diameters (~60) from representative images of control and CKO brains for both regions of the corpus callosum (MidCC, top and GCC, bottom). Following the analysis of previous studies [1,2] the ensemble of axon diameters from each of the 72 images was fitted with a *γ*-distribution. Similarly, Fig. 2 displays representative histograms of g-ratios and the fitted *γ*-distributions. A statistical analysis of the data supported the use of the γ-distribution to describe both the axon diameters and g-ratios. In 66/72 cases for axon diameters and 68/72 cases for g-ratios, the null hypothesis that the data were γ-distributed could not be rejected (Kolmogorov–Smirnov goodness of fit test, *P*<0.05), approximately the number of rejections you would expect by chance.

**Fig. 3:**
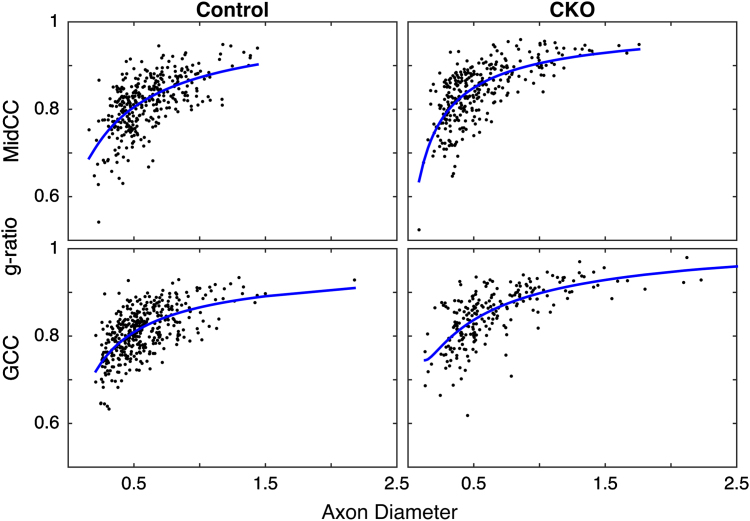
Representative g-ratio versus axon diameter scatter plots with log-linear fits (blue) from MidCC (top) and GCC (bottom) regions of the corpus callosum of control (left) and *Rictor* CKO (right) mice.

These images demonstrate the generally similar axon diameters but higher g-ratios found in CKO compared to control brains. Across all brains and images, the mean±SD axon diameter was 0.56±0.32 for controls and 0.62±0.37 in CKO brains, while the mean±SD g-ratio was 0.81±0.07 for controls and 0.85±0.08 in CKO brains. Fig. 3 shows scatter plots of g-ratio versus axon diameter for all 6 images from the representative control and CKO brains for both regions. In all cases, the relationship between axon diameter and *g* exhibited a curved shape seen in some previous studies [3,5], and appear to be well described by the log-linear equation proposed by Berthold et al. (*nl*=*C*_0_*+C*_1_*d+*C*_2_*log(*d*)); where *nl*=number of myelin lamellae and *d=*axon diameter (blue line) [3]. These observations are in contrast to a recent similar histological evaluation of the macaque corpus callosum [6] who showed only moderate linear correlations between g-ratio and axon diameter.
