# Supplementary figures and images for: Quantitative analysis of mouse corpus callosum from electron microscopy images
Source: Data Brief. 2015 Sep 3;5:124–8. doi: 10.1016/j.dib.2015.08.022 (PMC4576400; doi:10.1016/j.dib.2015.08.022)

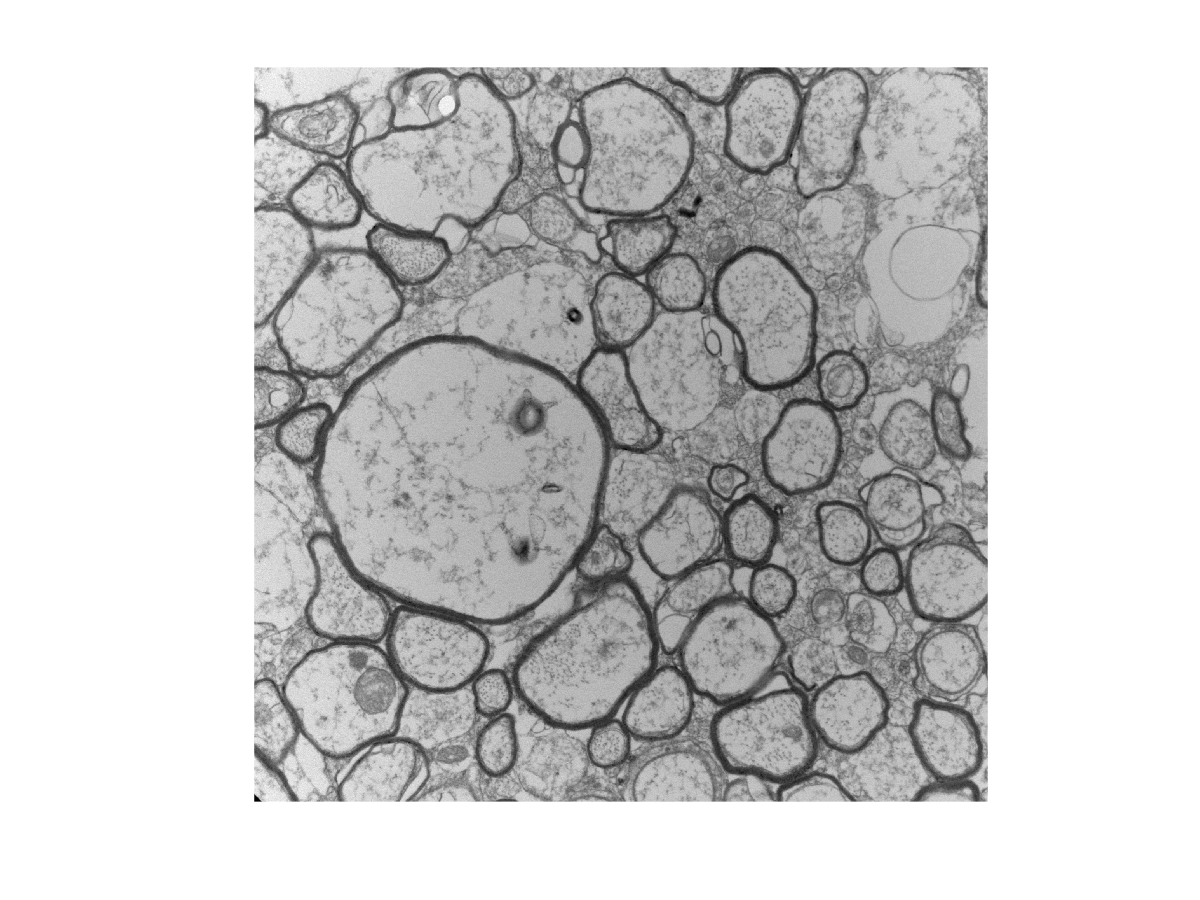

Supplement: Supplementary file 1 — Supplementary material [file mmc1.zip › Histology/CKO_5_GCC_1.tif]

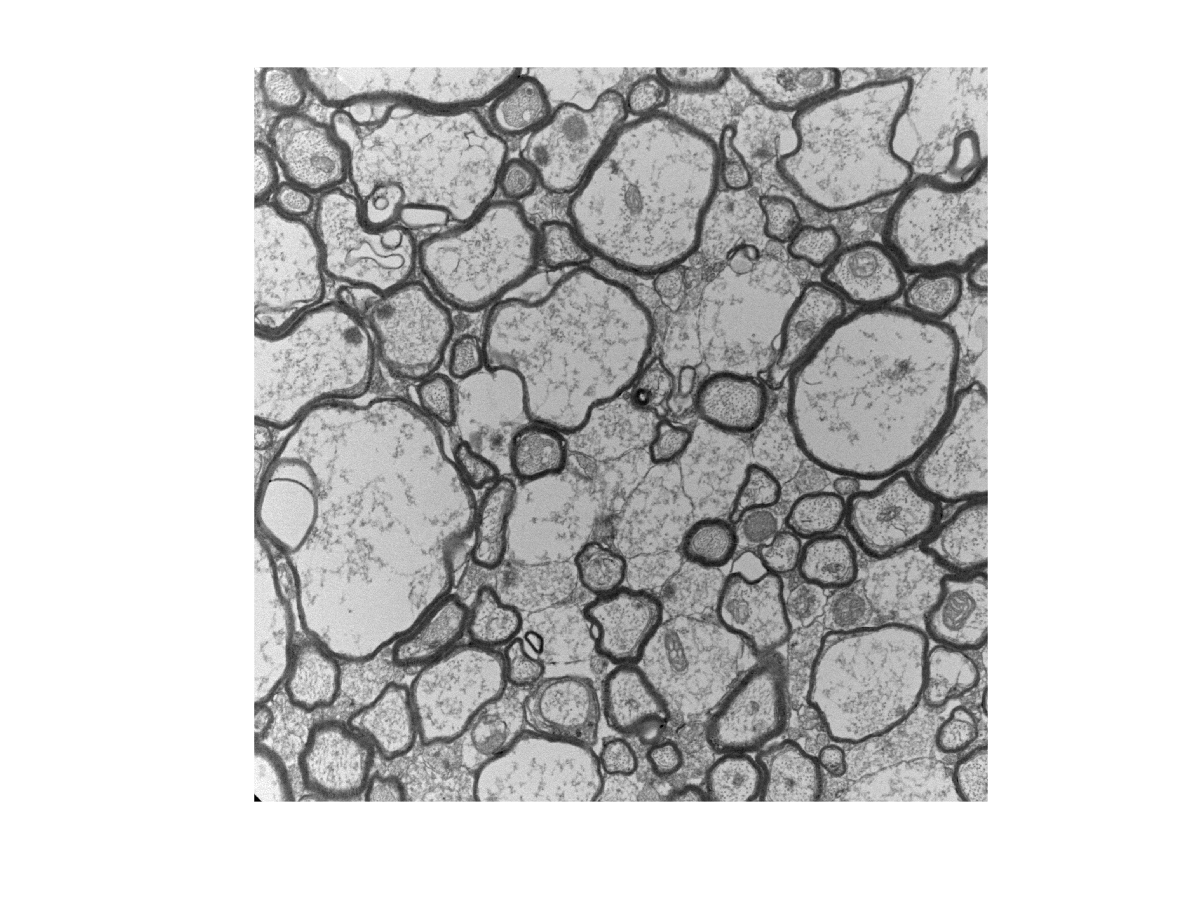

Supplement: Supplementary file 1 — Supplementary material [file mmc1.zip › Histology/CKO_5_GCC_2.tif]

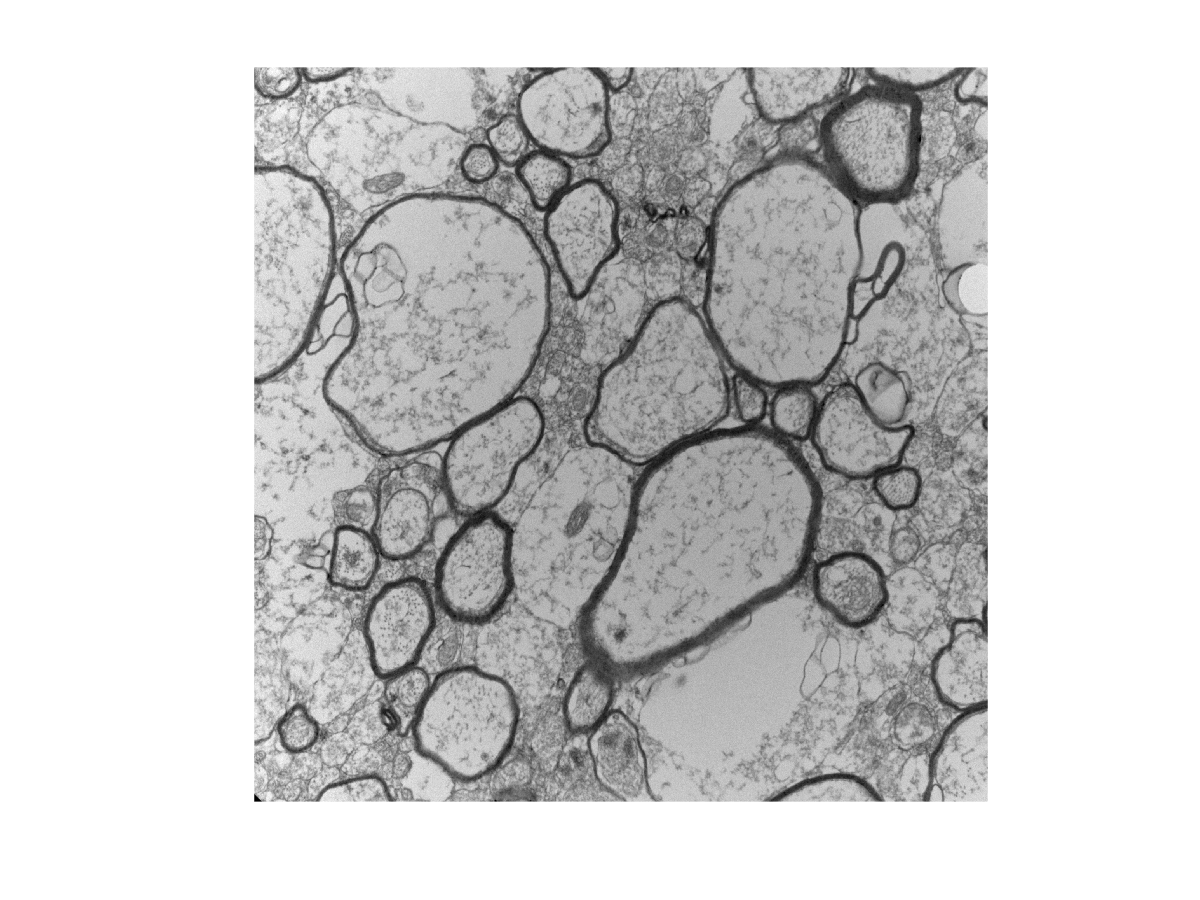

Supplement: Supplementary file 1 — Supplementary material [file mmc1.zip › Histology/CKO_5_GCC_3.tif]

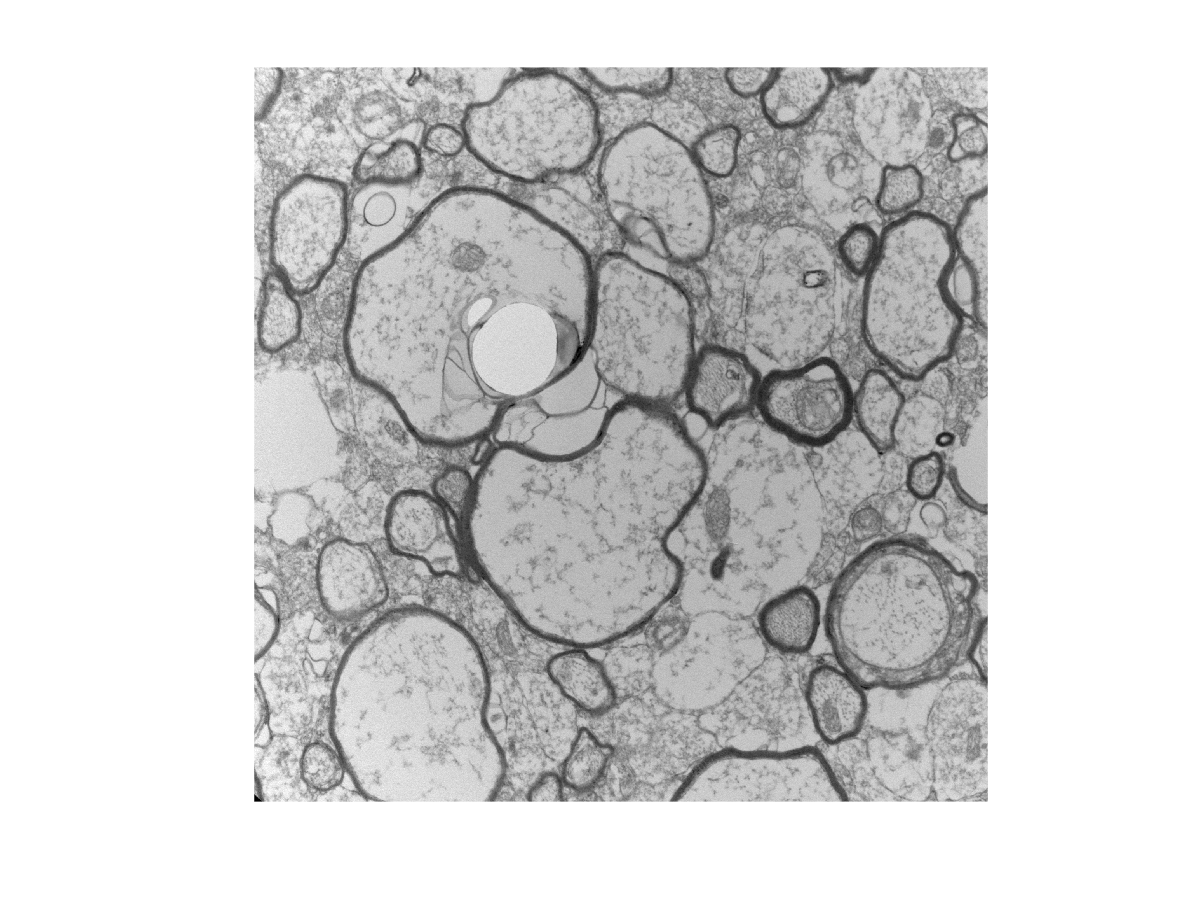

Supplement: Supplementary file 1 — Supplementary material [file mmc1.zip › Histology/CKO_5_GCC_4.tif]

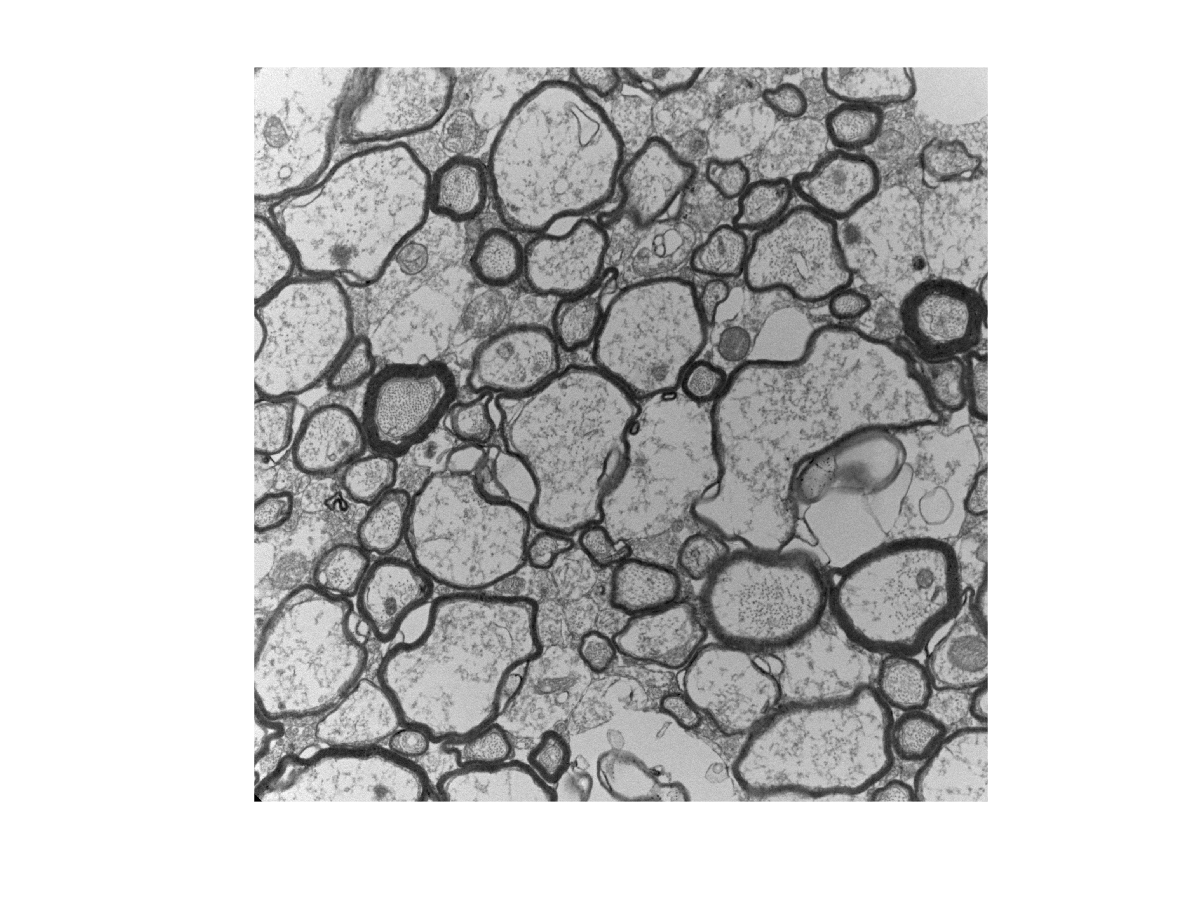

Supplement: Supplementary file 1 — Supplementary material [file mmc1.zip › Histology/CKO_5_GCC_5.tif]

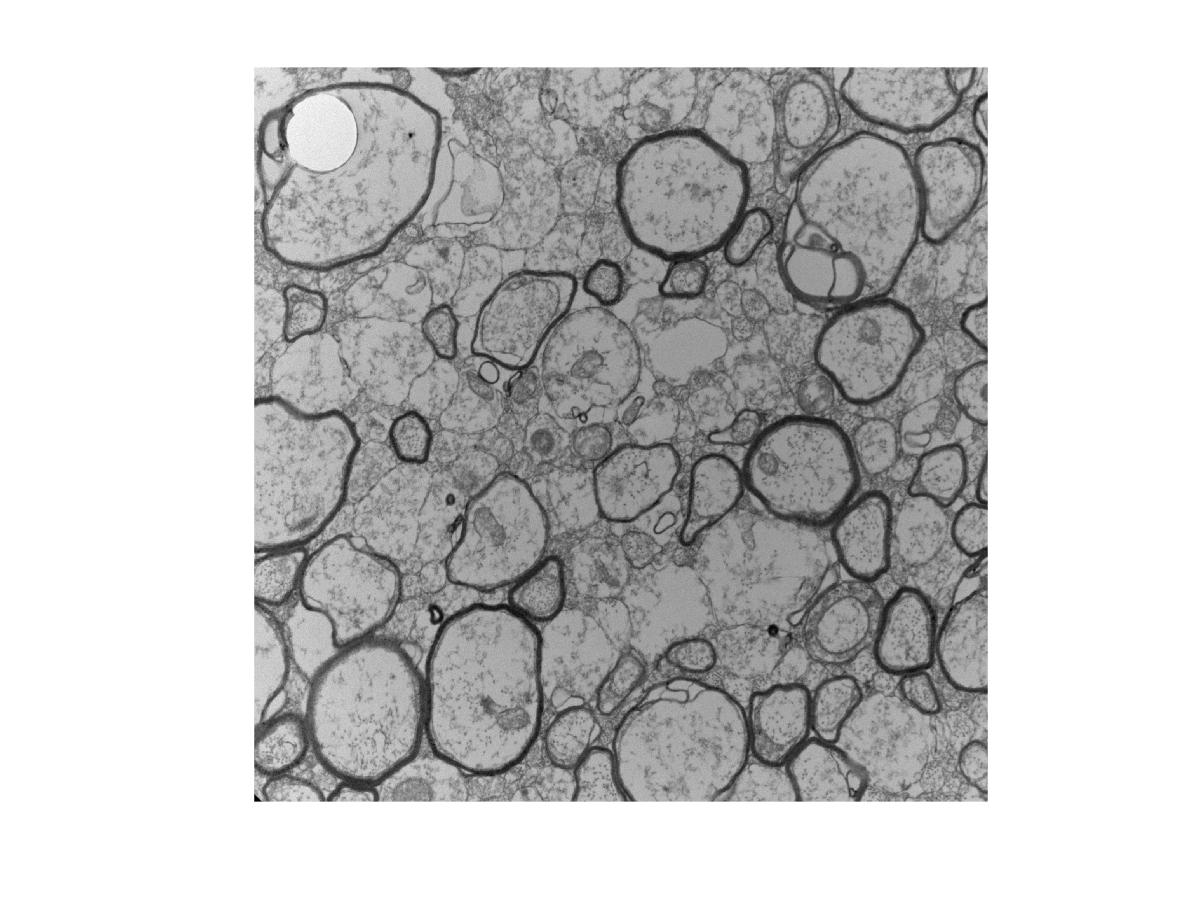

Supplement: Supplementary file 1 — Supplementary material [file mmc1.zip › Histology/CKO_5_GCC_6.tif]

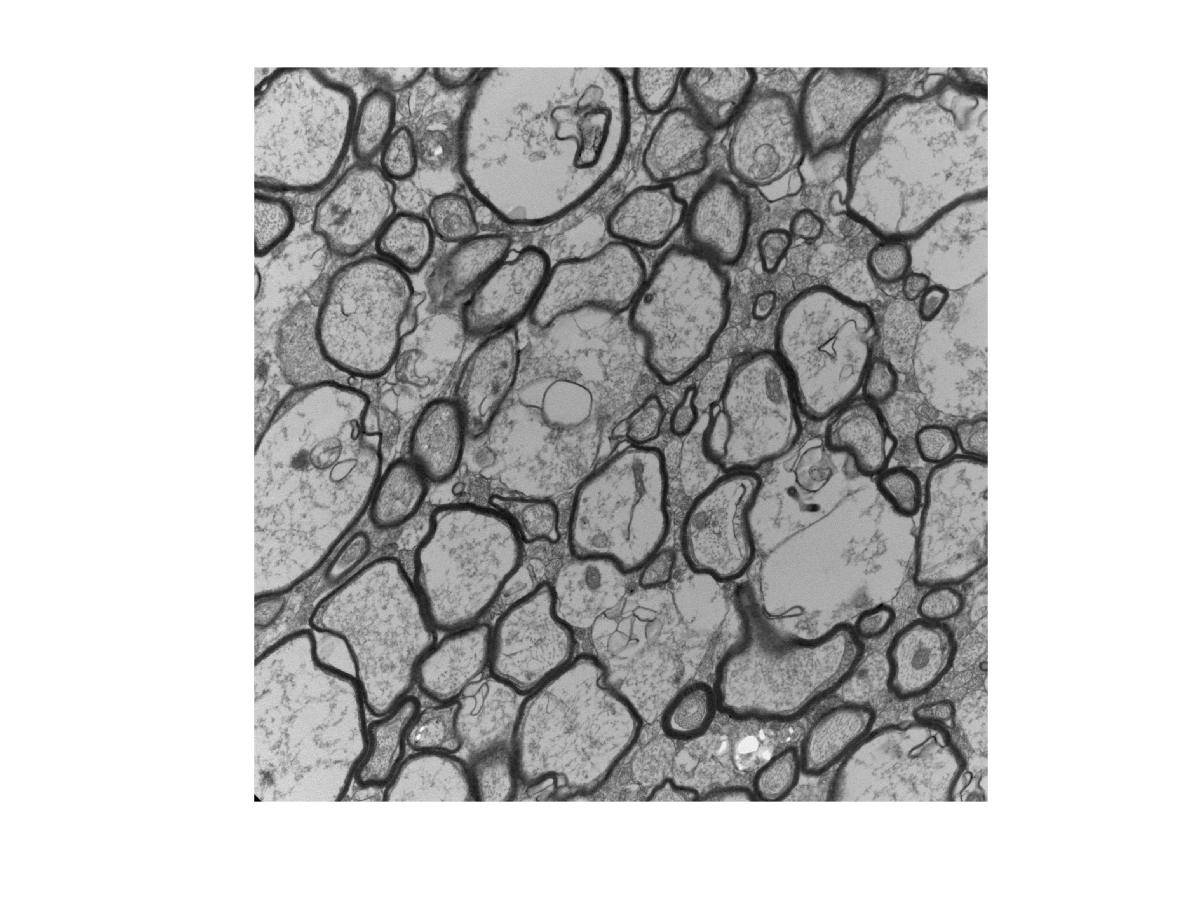

Supplement: Supplementary file 1 — Supplementary material [file mmc1.zip › Histology/CKO_5_MidCC_1.tif]

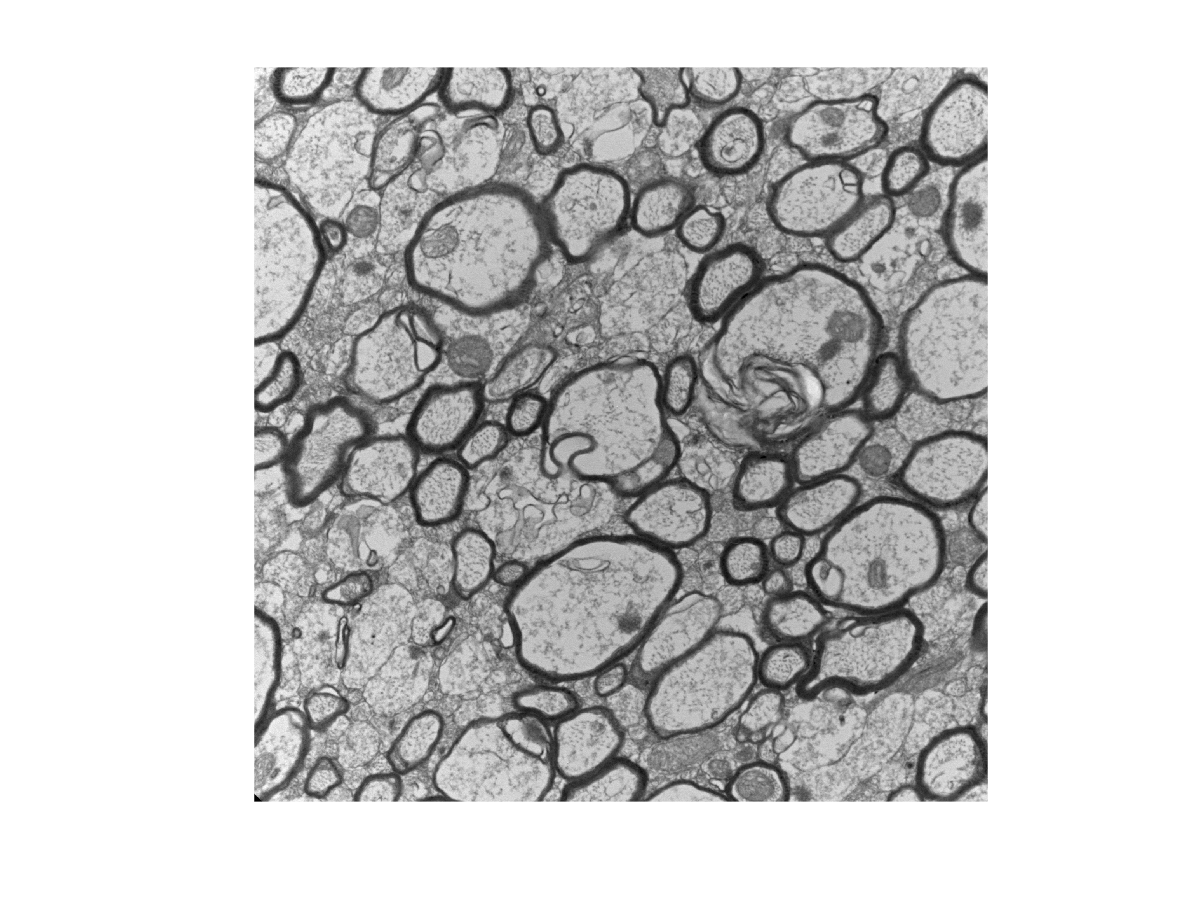

Supplement: Supplementary file 1 — Supplementary material [file mmc1.zip › Histology/CKO_5_MidCC_2.tif]

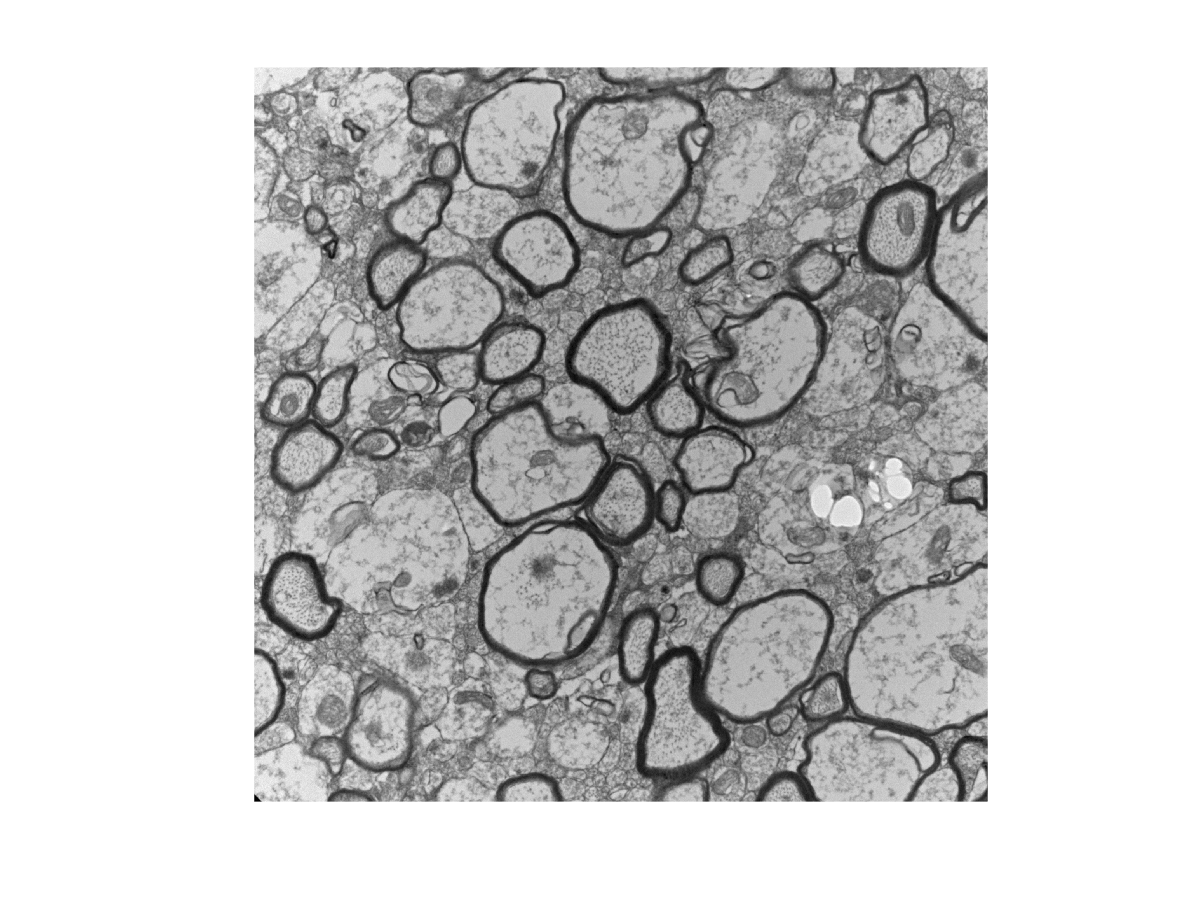

Supplement: Supplementary file 1 — Supplementary material [file mmc1.zip › Histology/CKO_5_MidCC_3.tif]

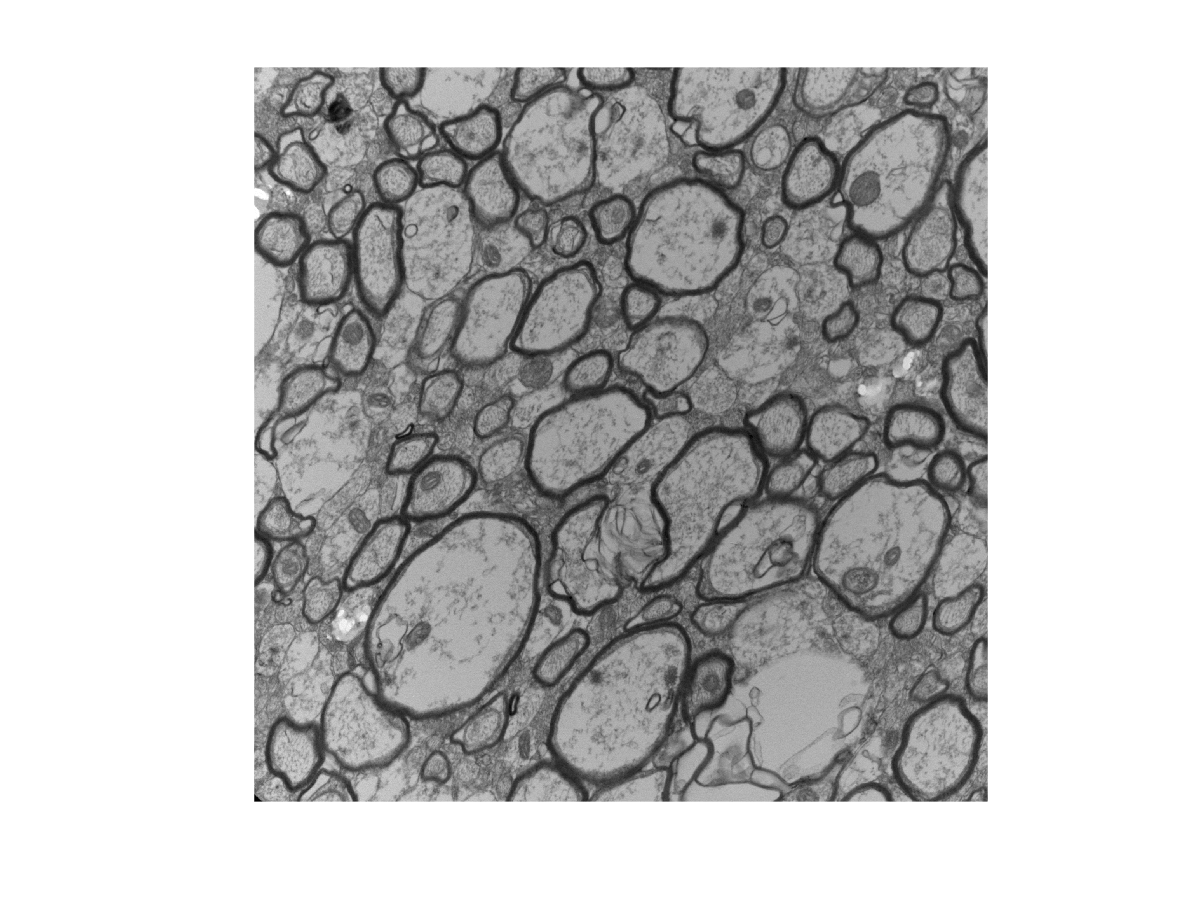

Supplement: Supplementary file 1 — Supplementary material [file mmc1.zip › Histology/CKO_5_MidCC_4.tif]

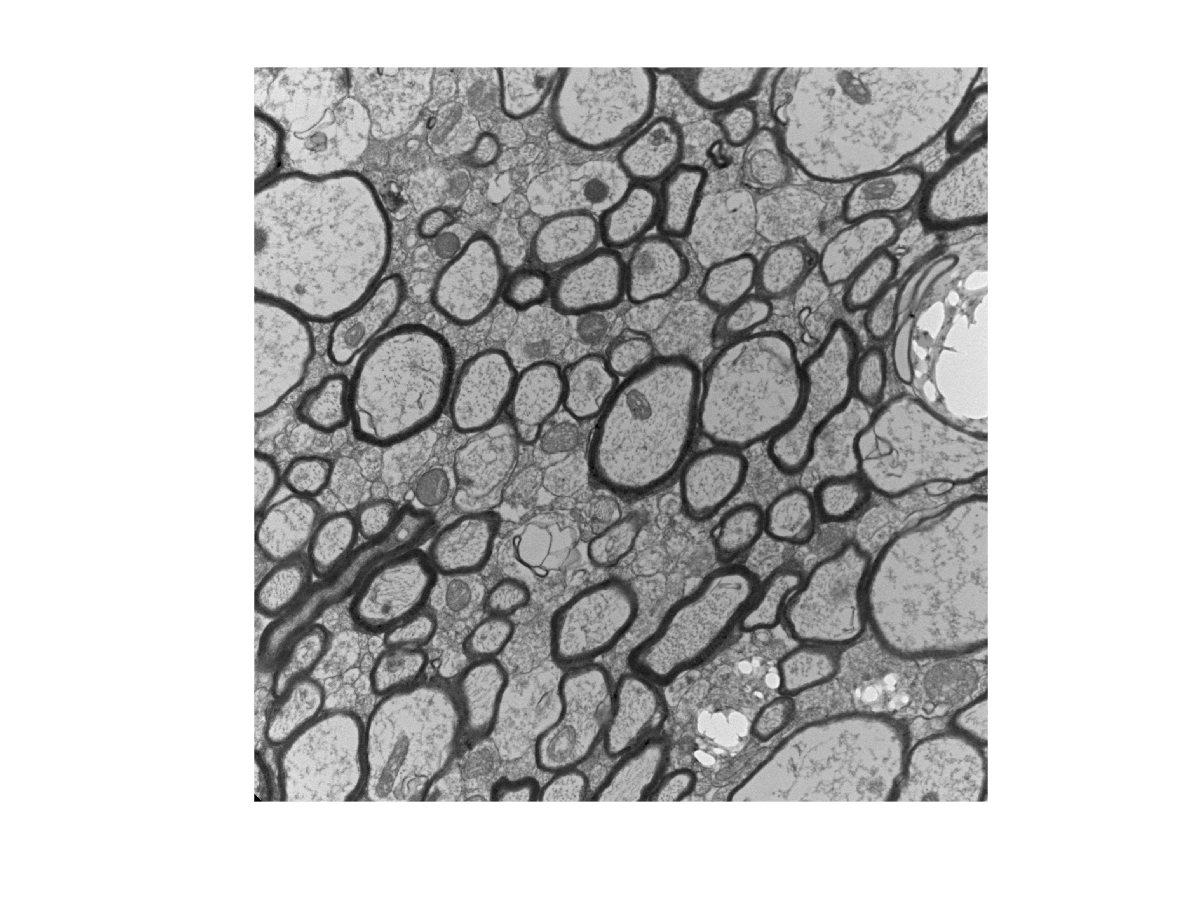

Supplement: Supplementary file 1 — Supplementary material [file mmc1.zip › Histology/CKO_5_MidCC_5.tif]

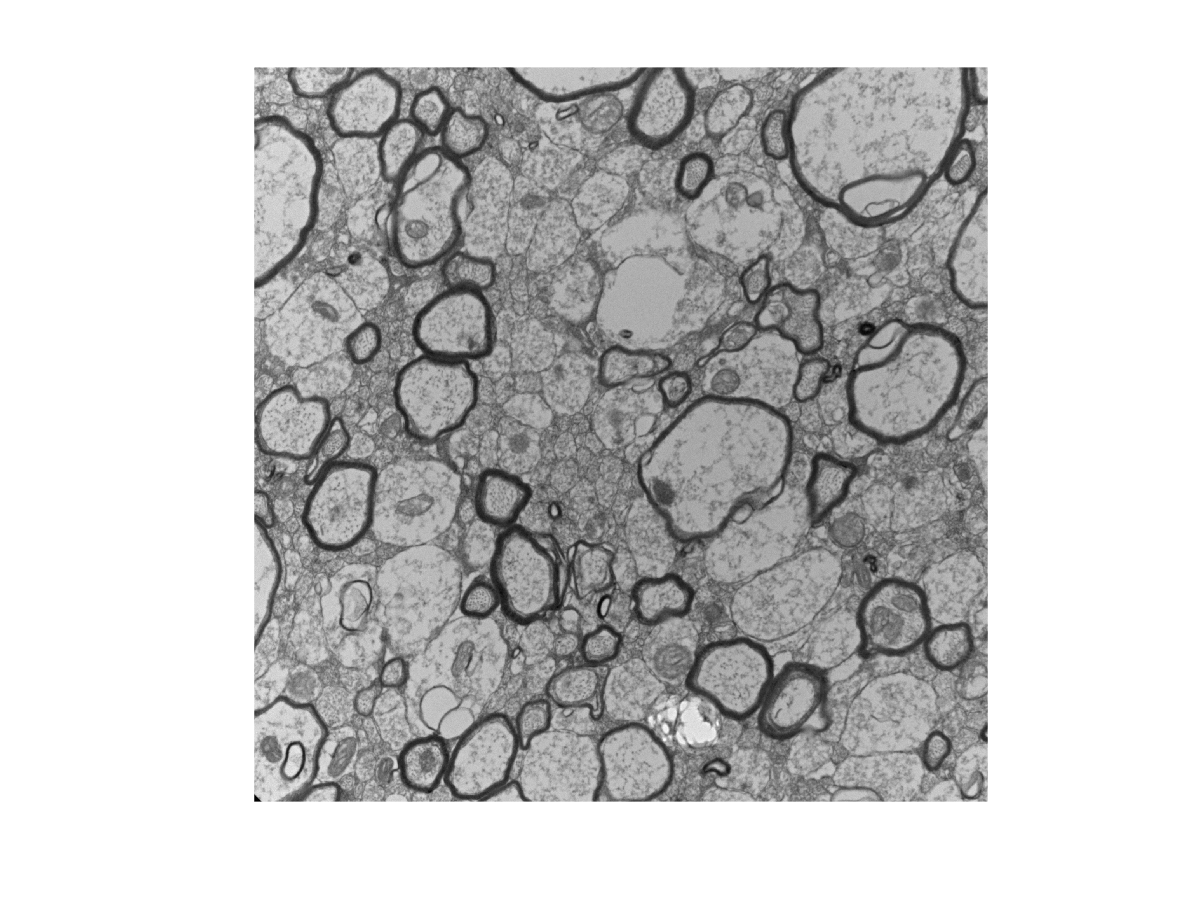

Supplement: Supplementary file 1 — Supplementary material [file mmc1.zip › Histology/CKO_5_MidCC_6.tif]

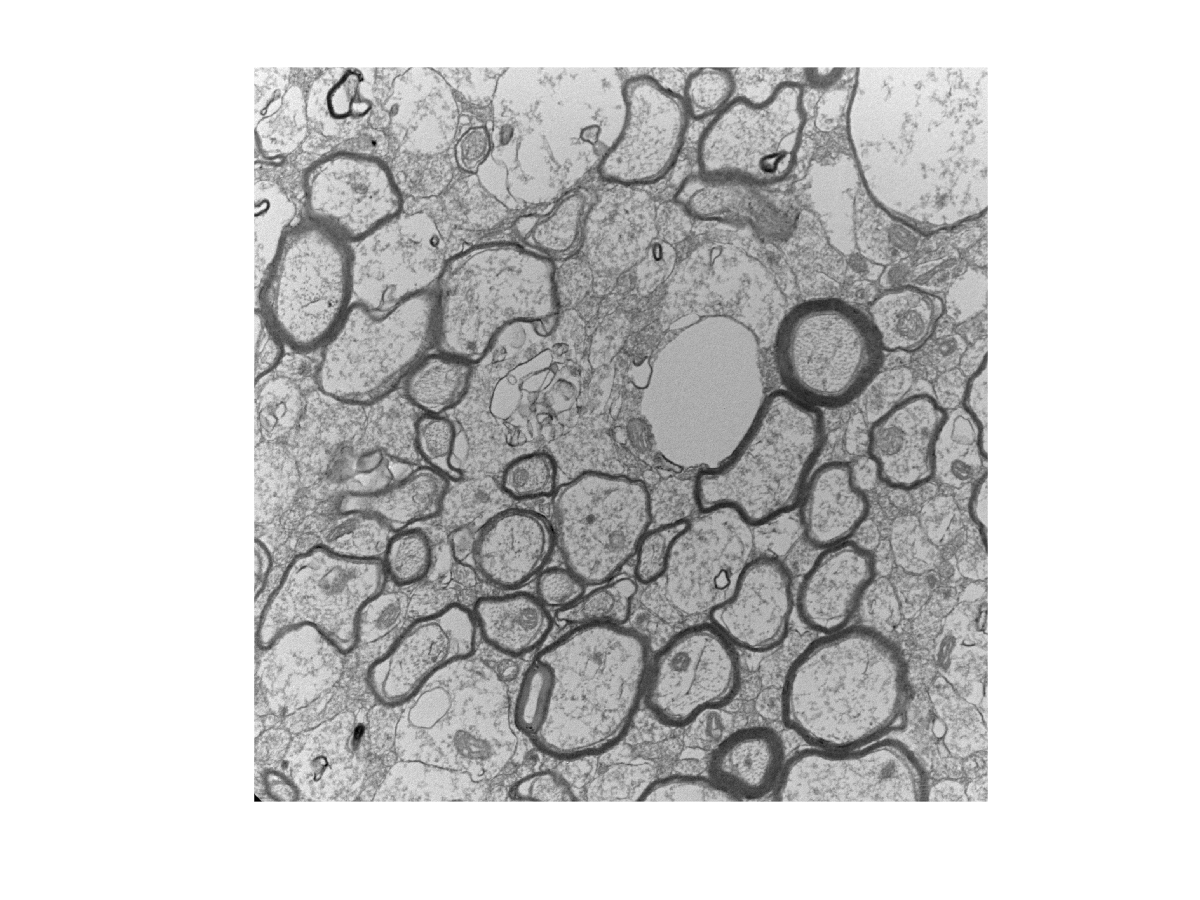

Supplement: Supplementary file 1 — Supplementary material [file mmc1.zip › Histology/CKO_6_GCC_1.tif]

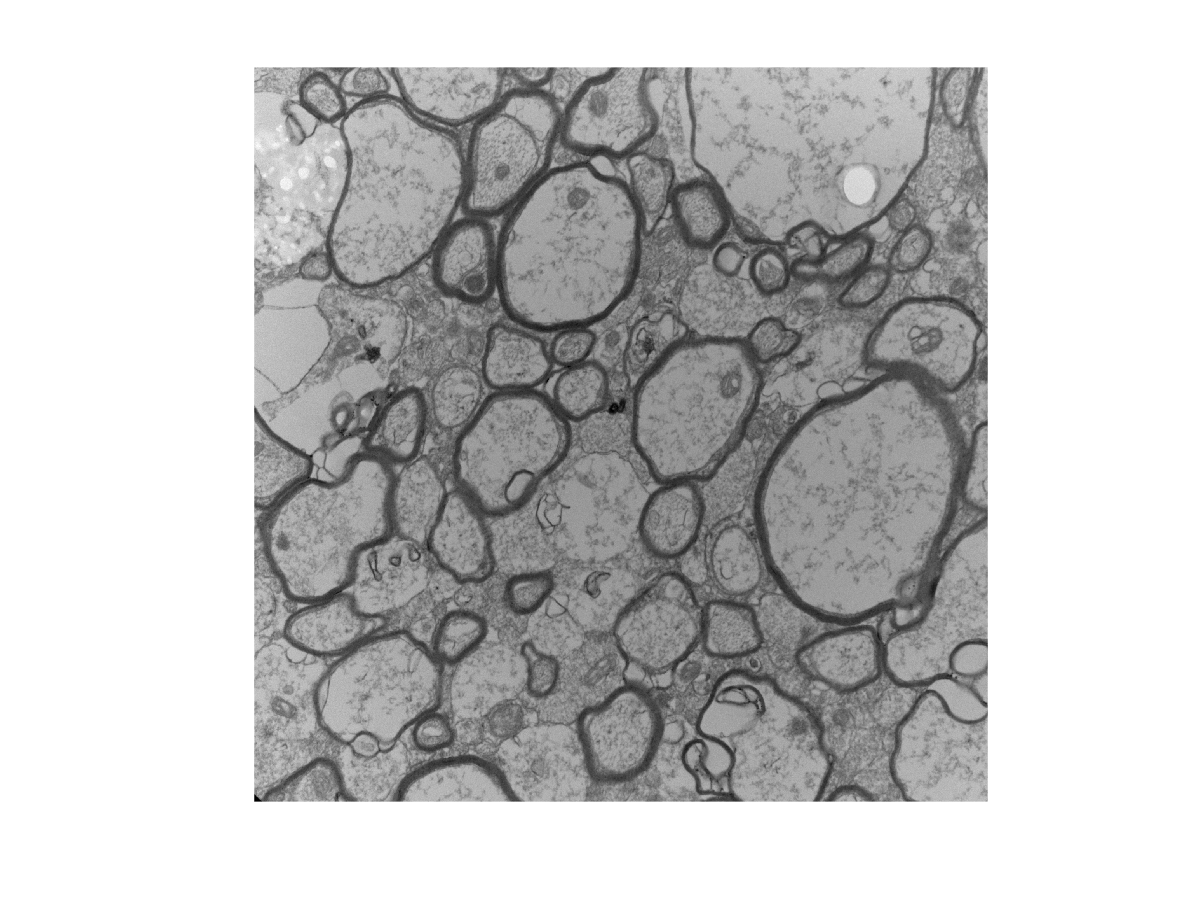

Supplement: Supplementary file 1 — Supplementary material [file mmc1.zip › Histology/CKO_6_GCC_2.tif]

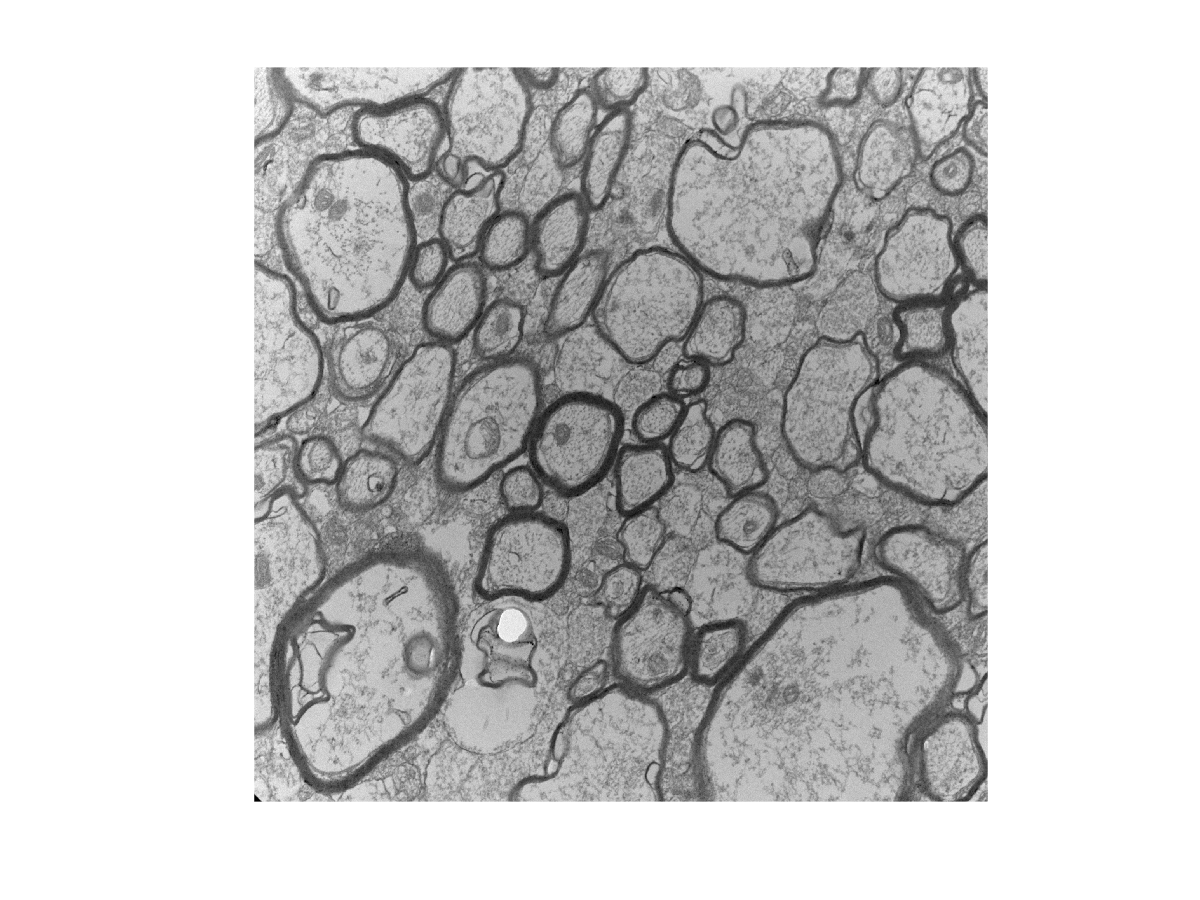

Supplement: Supplementary file 1 — Supplementary material [file mmc1.zip › Histology/CKO_6_GCC_3.tif]

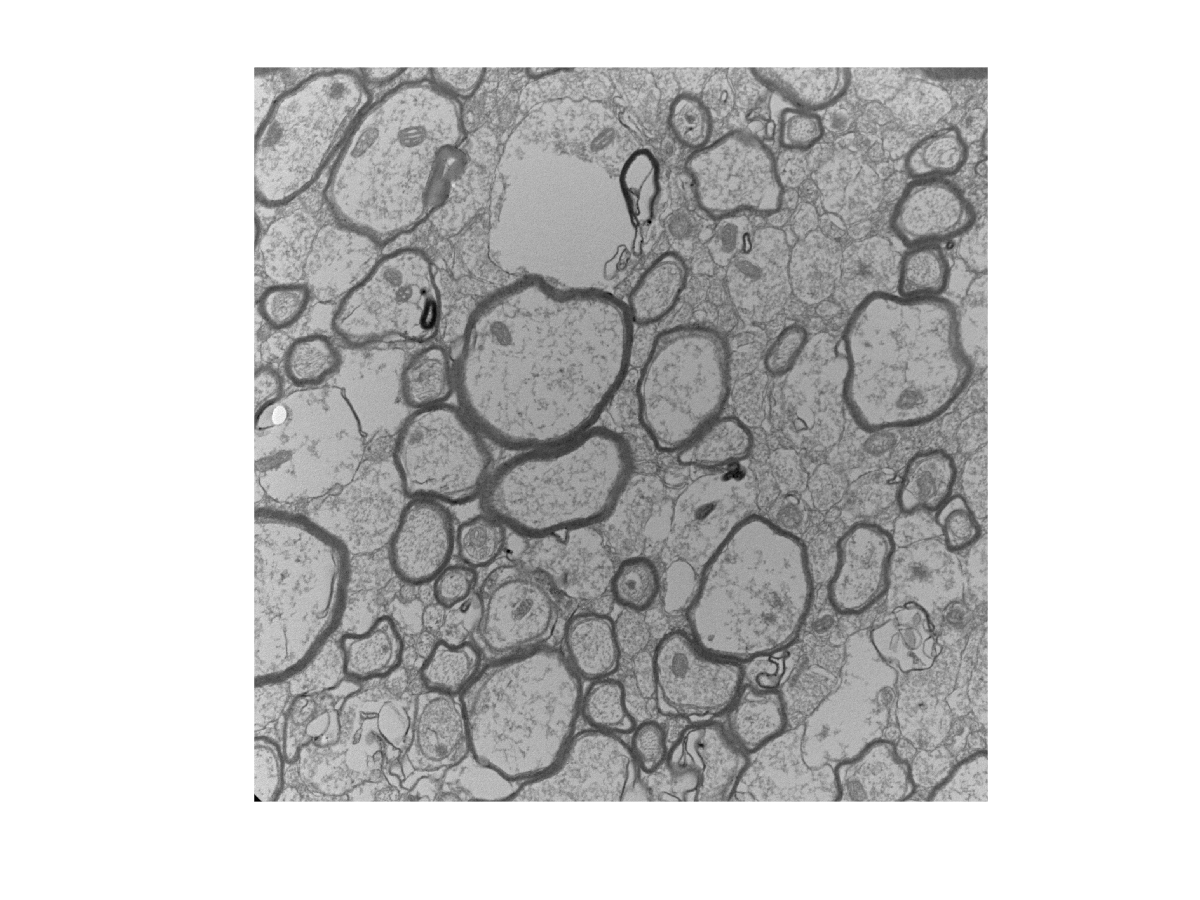

Supplement: Supplementary file 1 — Supplementary material [file mmc1.zip › Histology/CKO_6_GCC_4.tif]

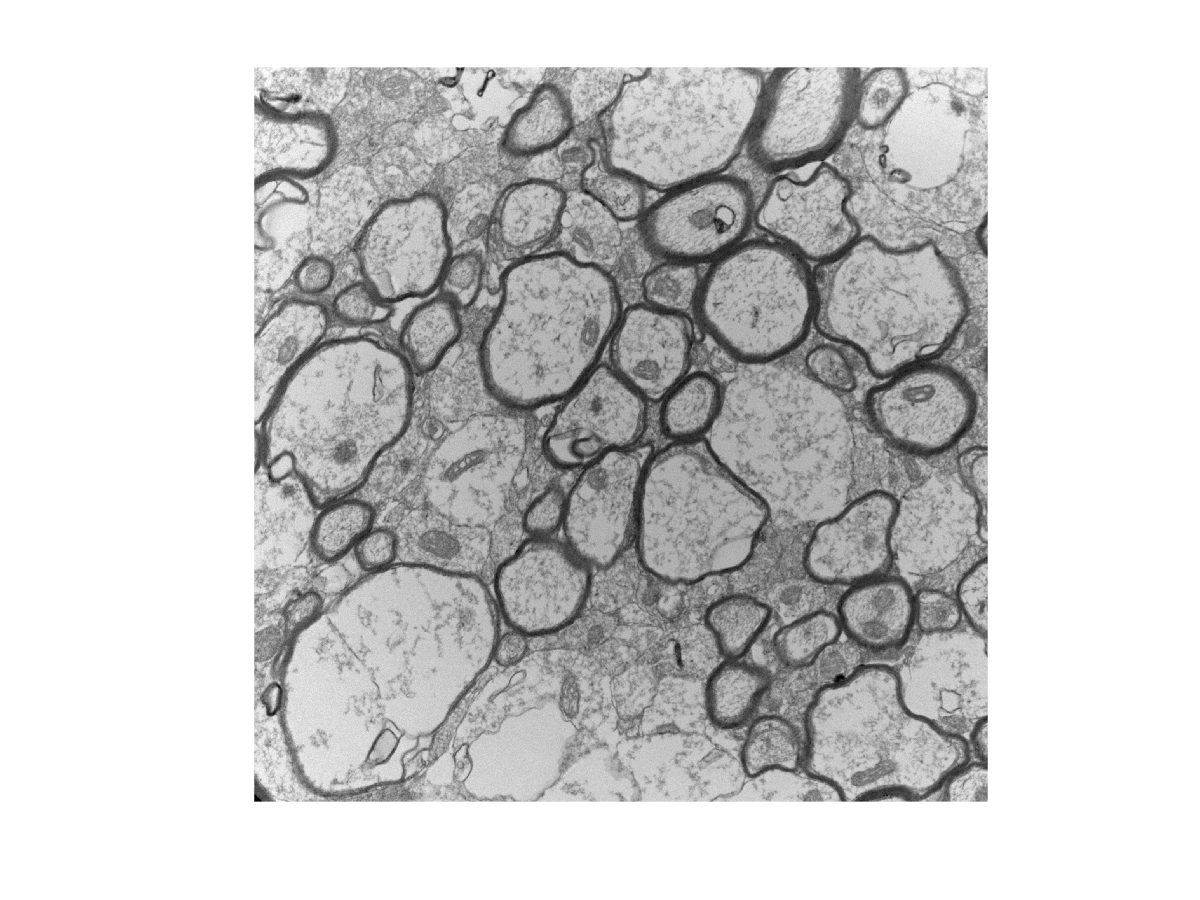

Supplement: Supplementary file 1 — Supplementary material [file mmc1.zip › Histology/CKO_6_GCC_5.tif]

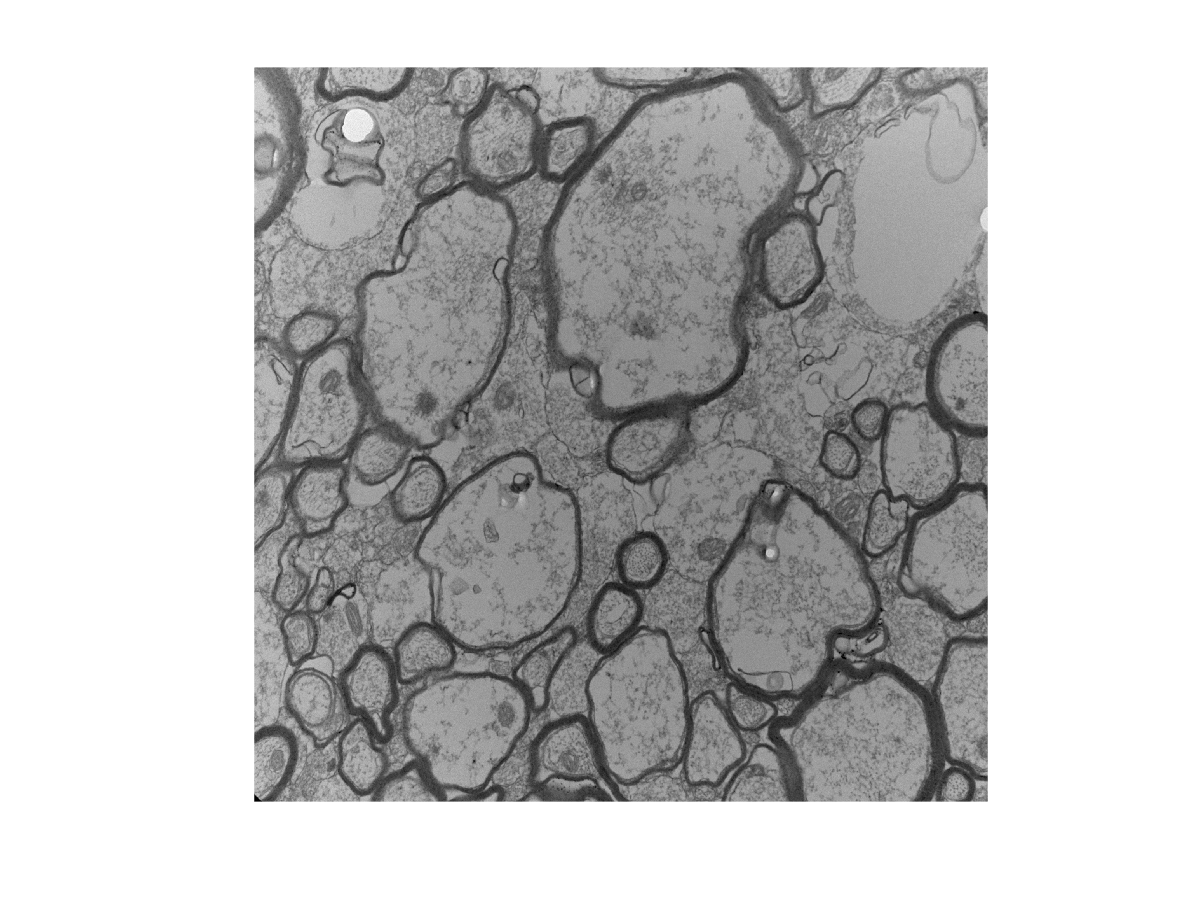

Supplement: Supplementary file 1 — Supplementary material [file mmc1.zip › Histology/CKO_6_GCC_6.tif]

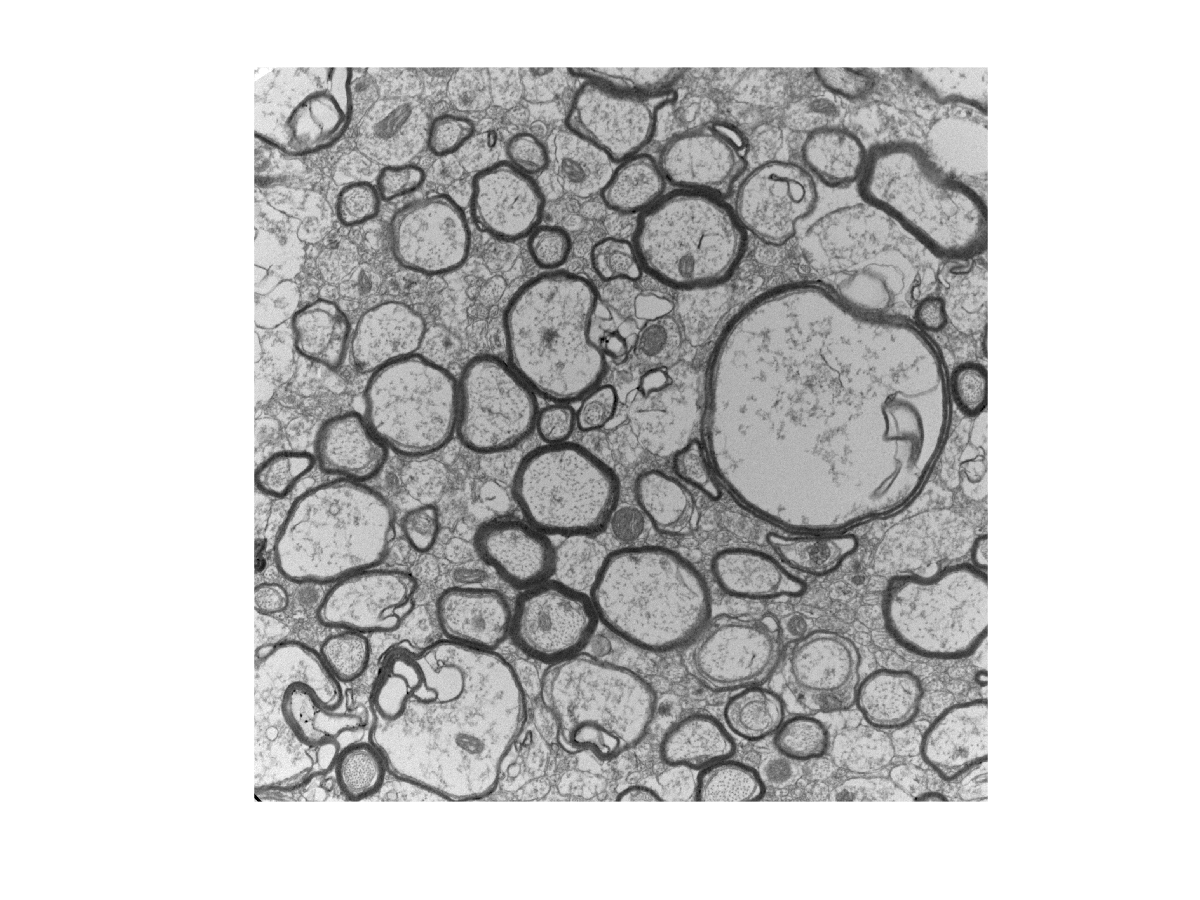

Supplement: Supplementary file 1 — Supplementary material [file mmc1.zip › Histology/CKO_6_MidCC_1.tif]

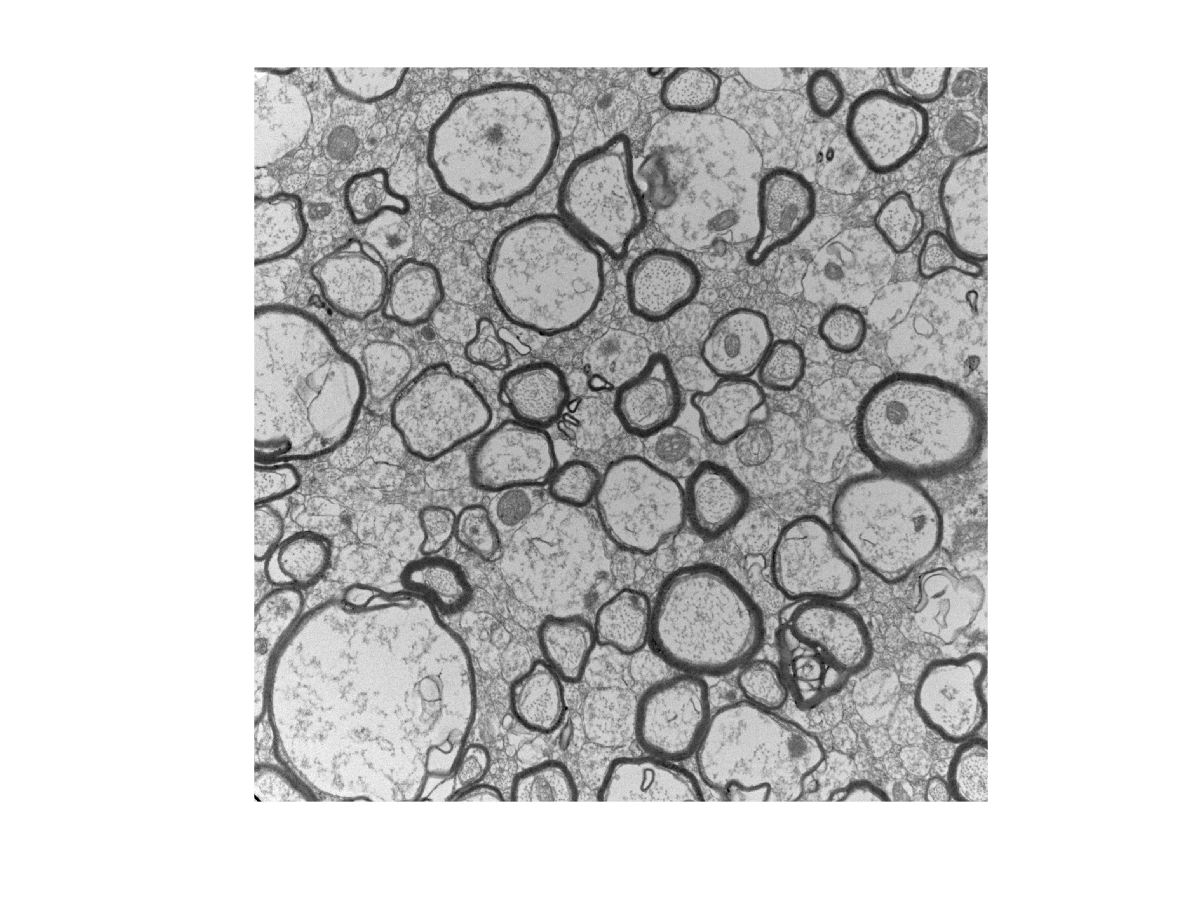

Supplement: Supplementary file 1 — Supplementary material [file mmc1.zip › Histology/CKO_6_MidCC_2.tif]

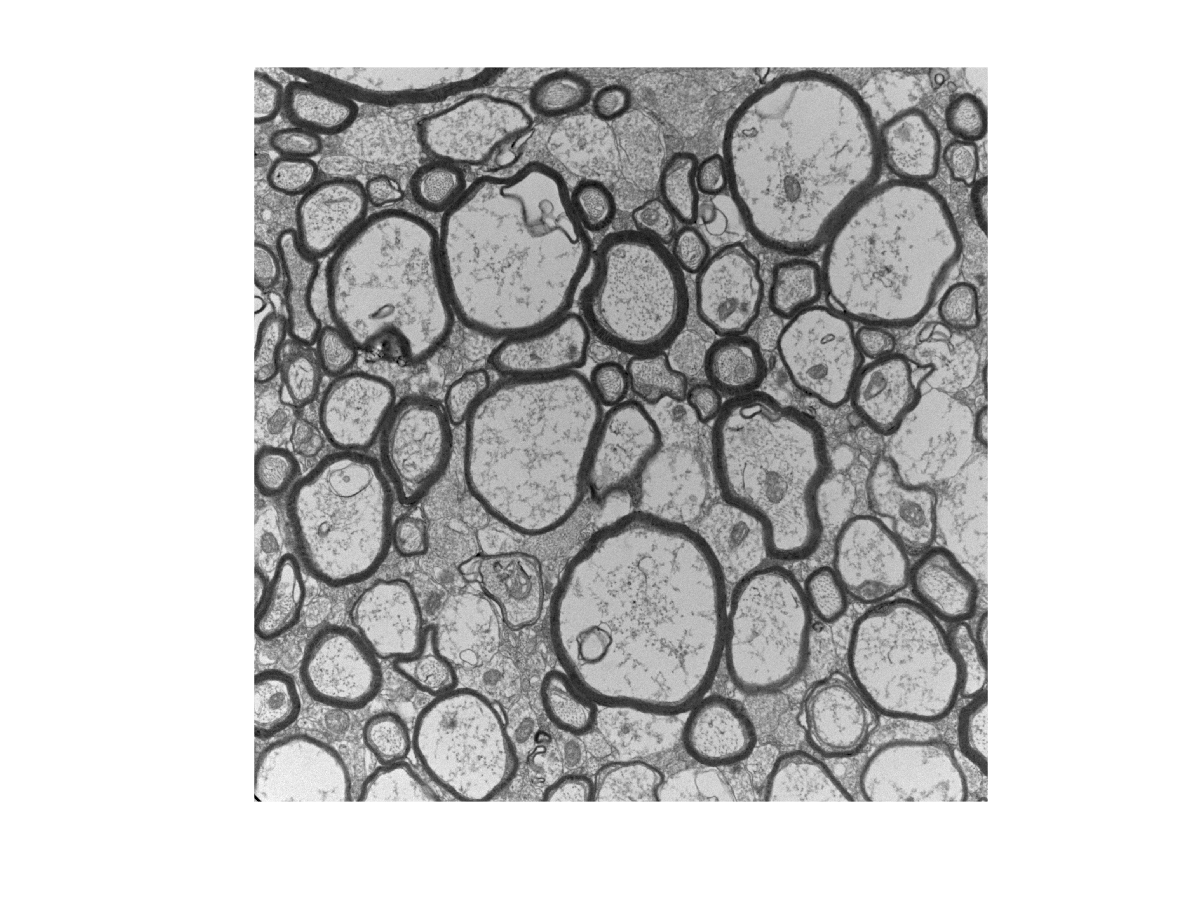

Supplement: Supplementary file 1 — Supplementary material [file mmc1.zip › Histology/CKO_6_MidCC_3.tif]

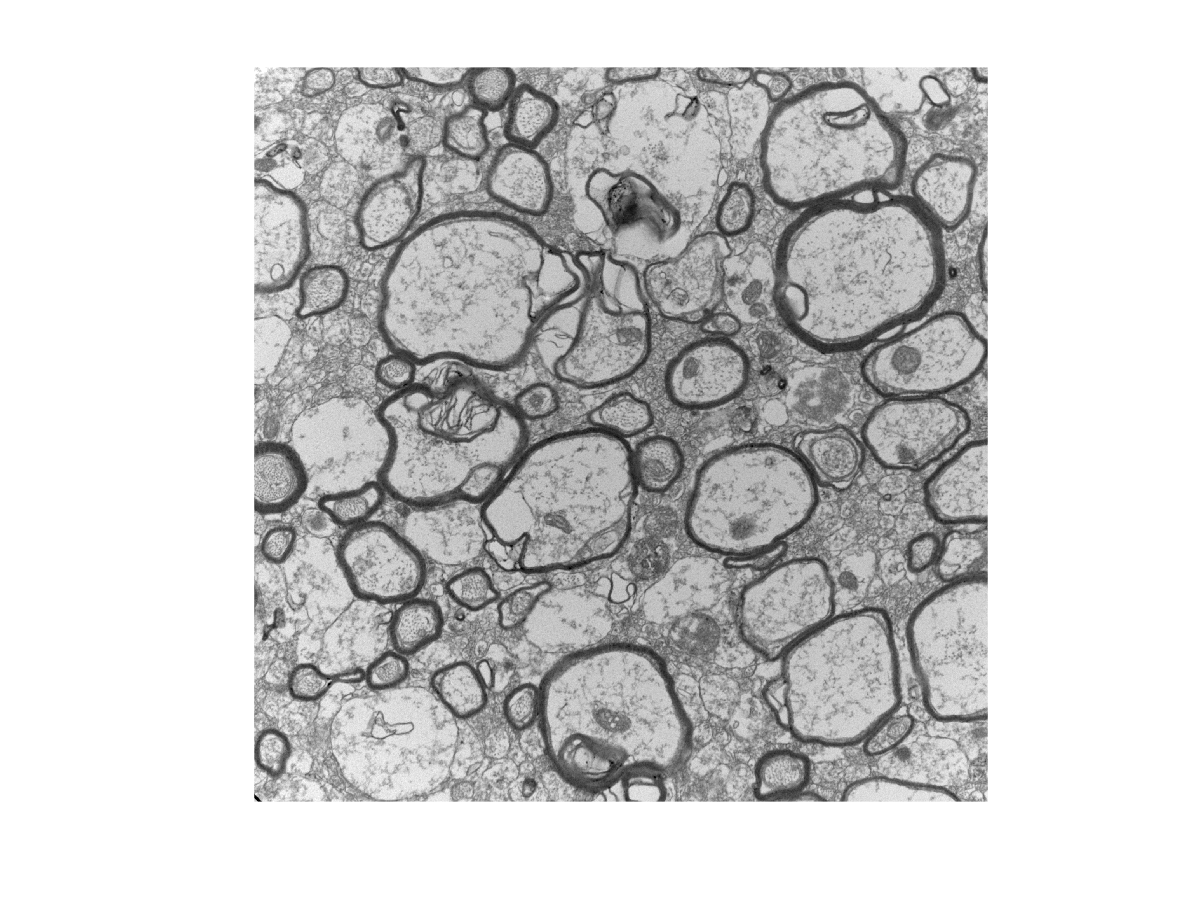

Supplement: Supplementary file 1 — Supplementary material [file mmc1.zip › Histology/CKO_6_MidCC_4.tif]

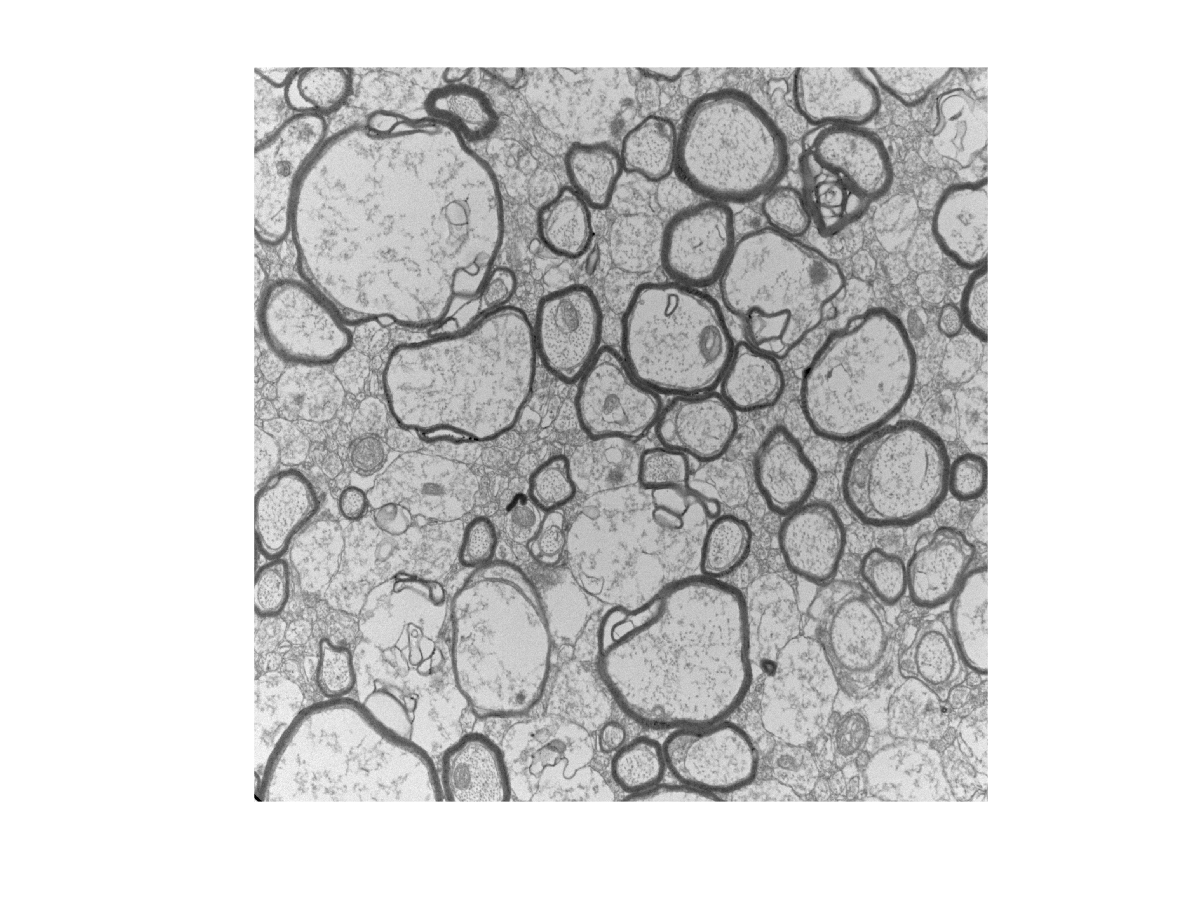

Supplement: Supplementary file 1 — Supplementary material [file mmc1.zip › Histology/CKO_6_MidCC_5.tif]

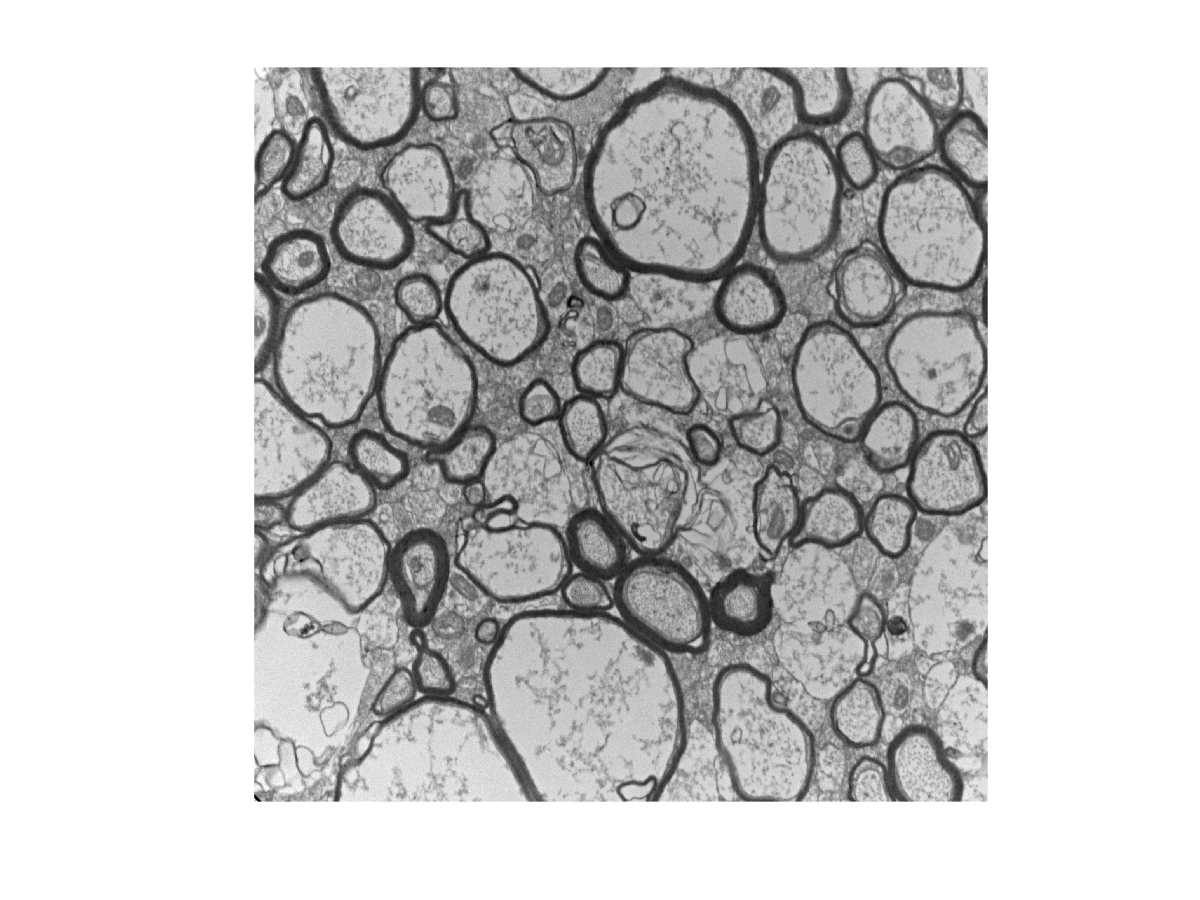

Supplement: Supplementary file 1 — Supplementary material [file mmc1.zip › Histology/CKO_6_MidCC_6.tif]

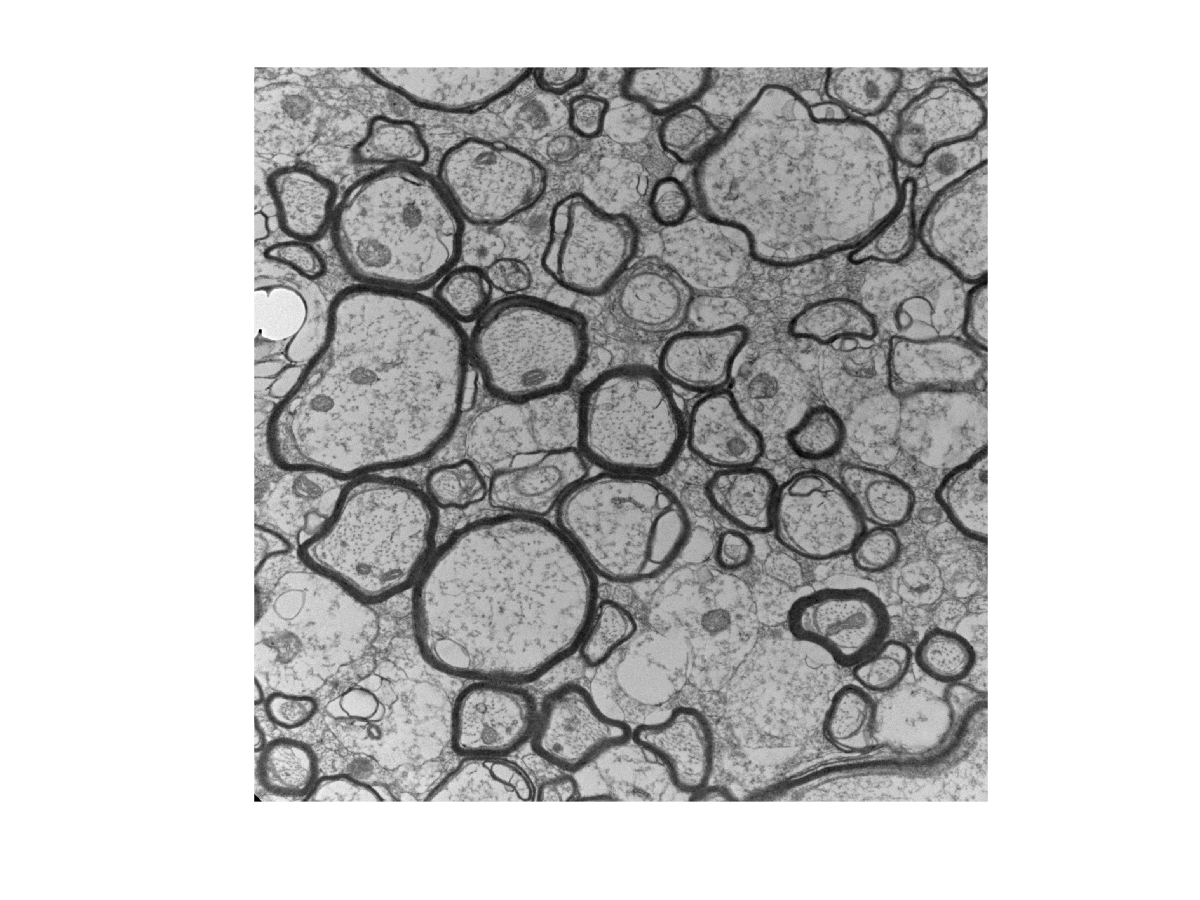

Supplement: Supplementary file 1 — Supplementary material [file mmc1.zip › Histology/Control_1_GCC_1.tif]

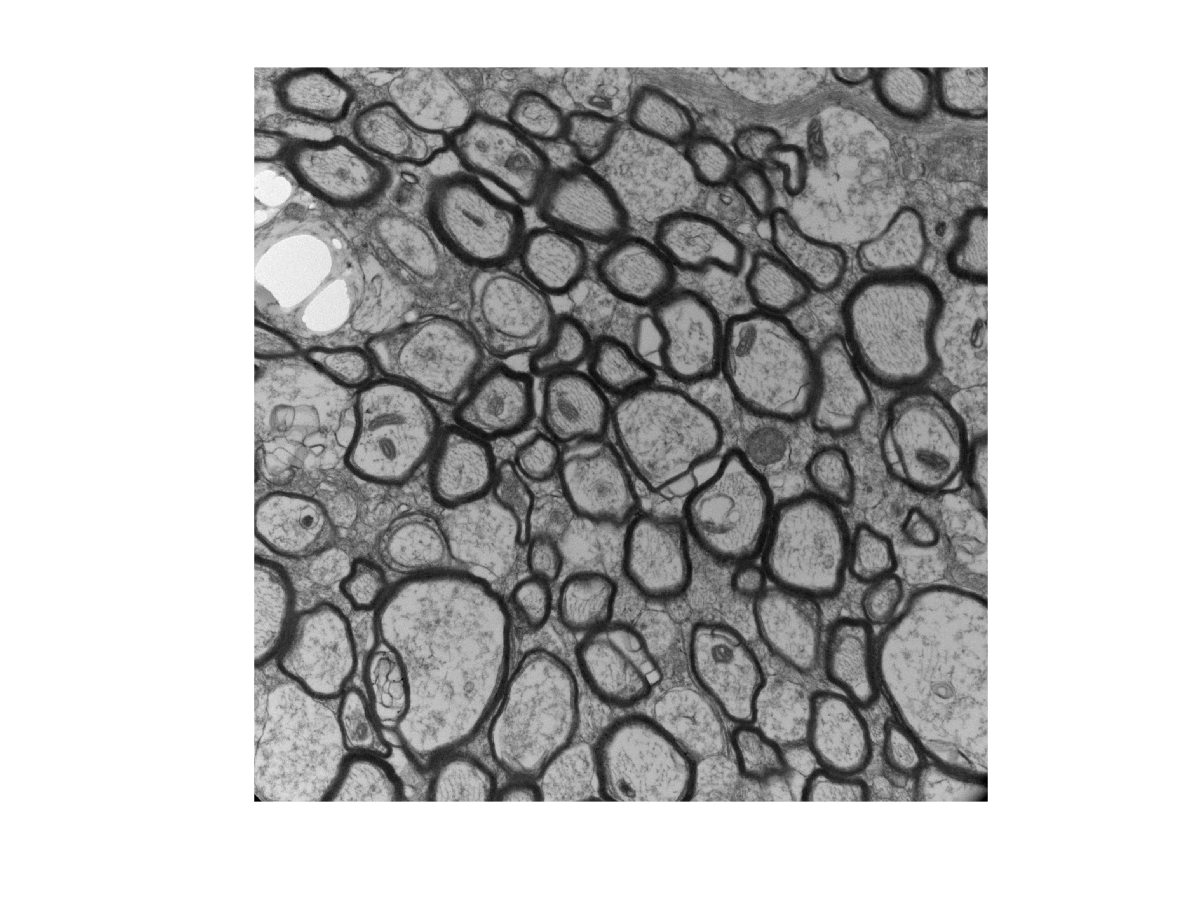

Supplement: Supplementary file 1 — Supplementary material [file mmc1.zip › Histology/Control_1_GCC_2.tif]

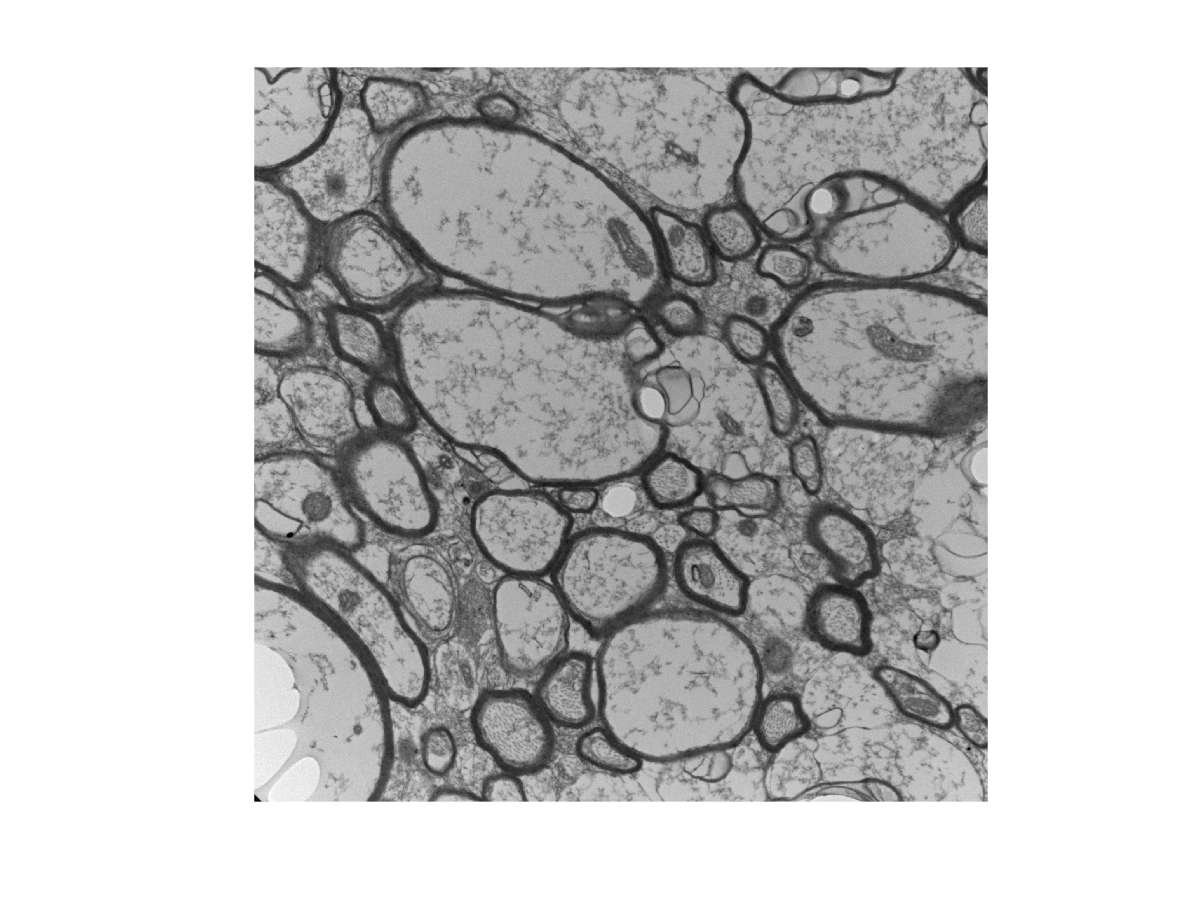

Supplement: Supplementary file 1 — Supplementary material [file mmc1.zip › Histology/Control_1_GCC_3.tif]

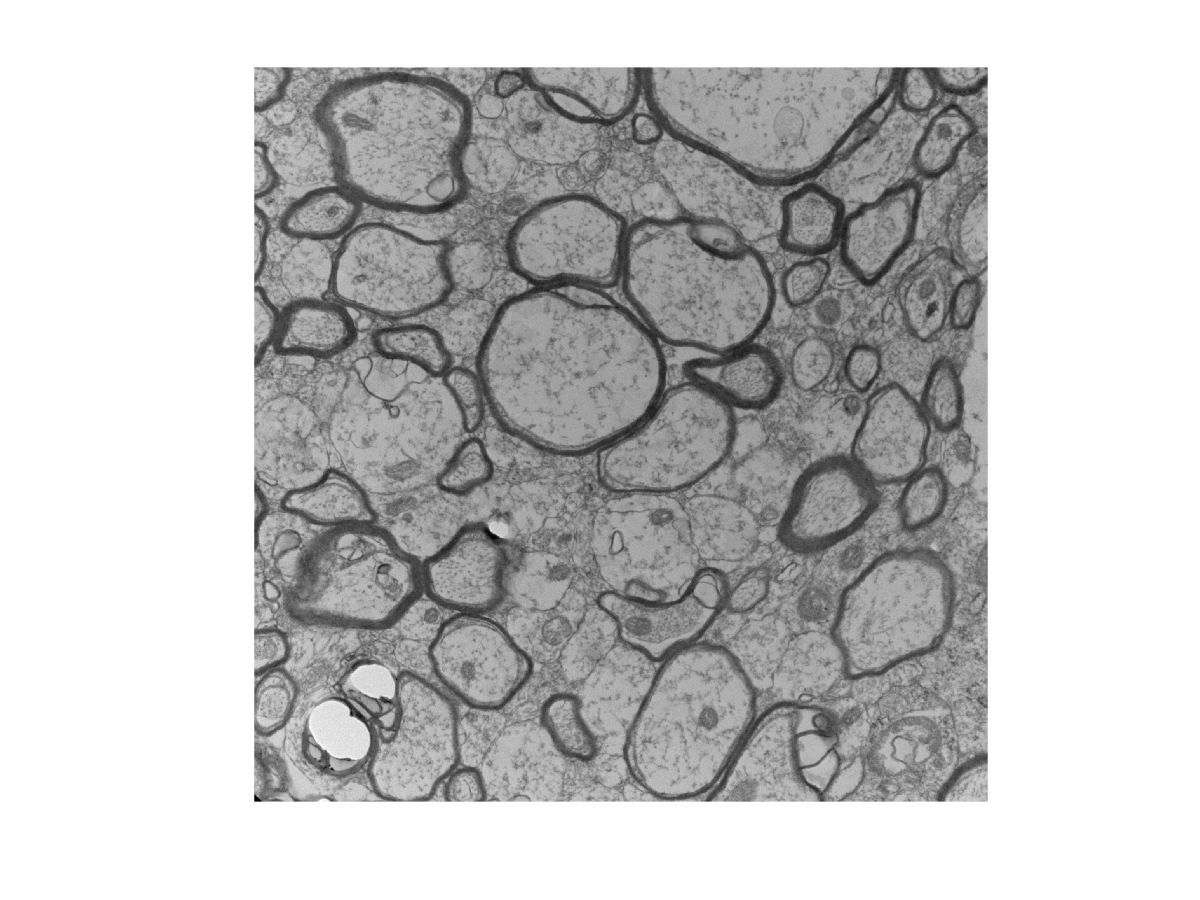

Supplement: Supplementary file 1 — Supplementary material [file mmc1.zip › Histology/Control_1_GCC_4.tif]

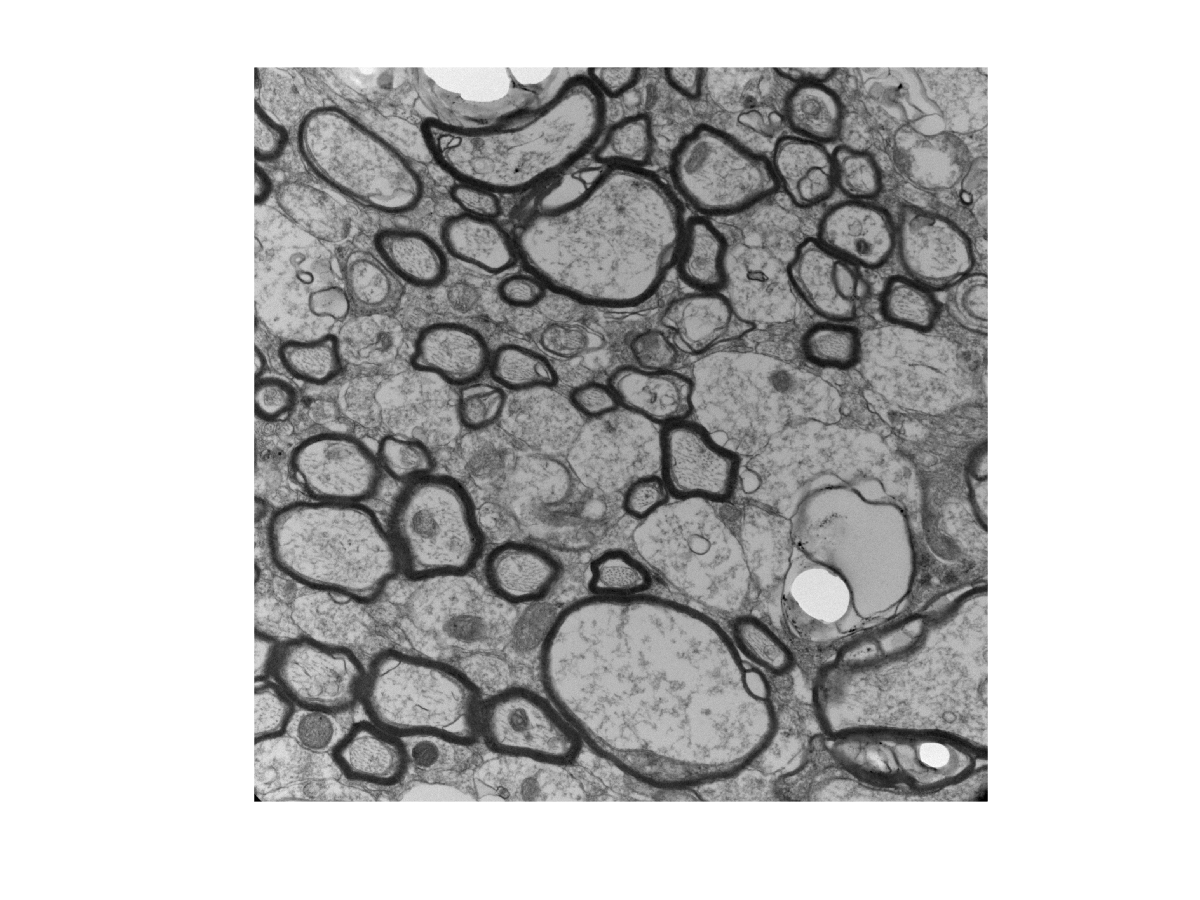

Supplement: Supplementary file 1 — Supplementary material [file mmc1.zip › Histology/Control_1_GCC_5.tif]

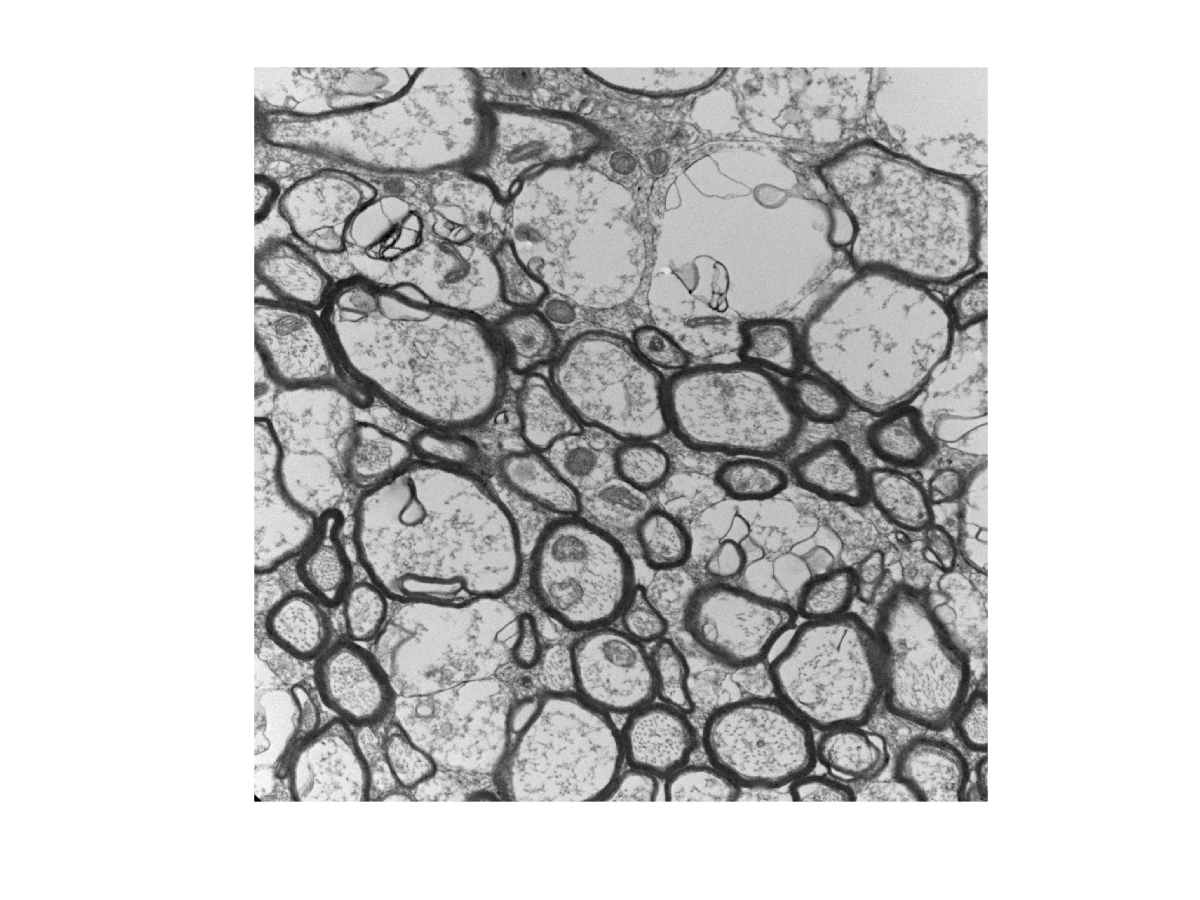

Supplement: Supplementary file 1 — Supplementary material [file mmc1.zip › Histology/Control_1_GCC_6.tif]

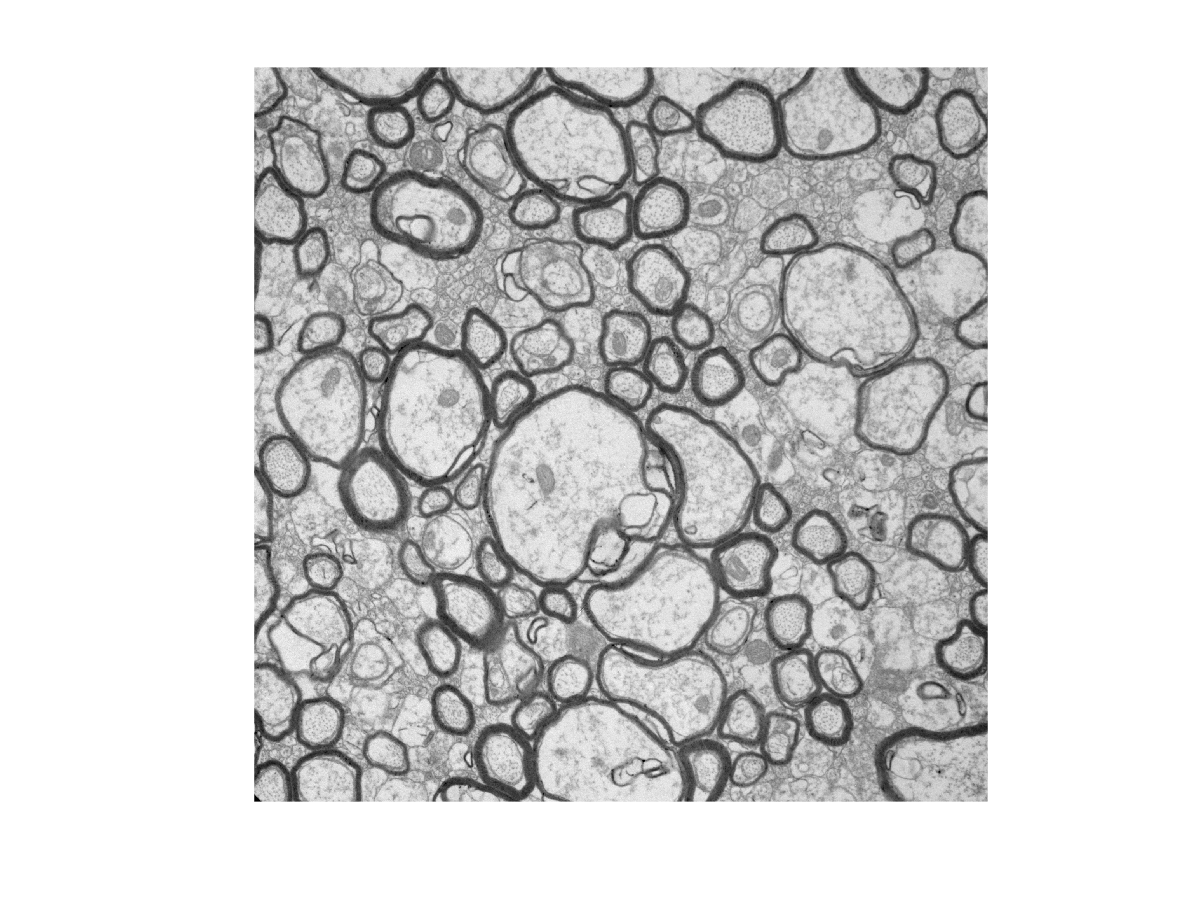

Supplement: Supplementary file 1 — Supplementary material [file mmc1.zip › Histology/Control_1_MidCC_1.tif]

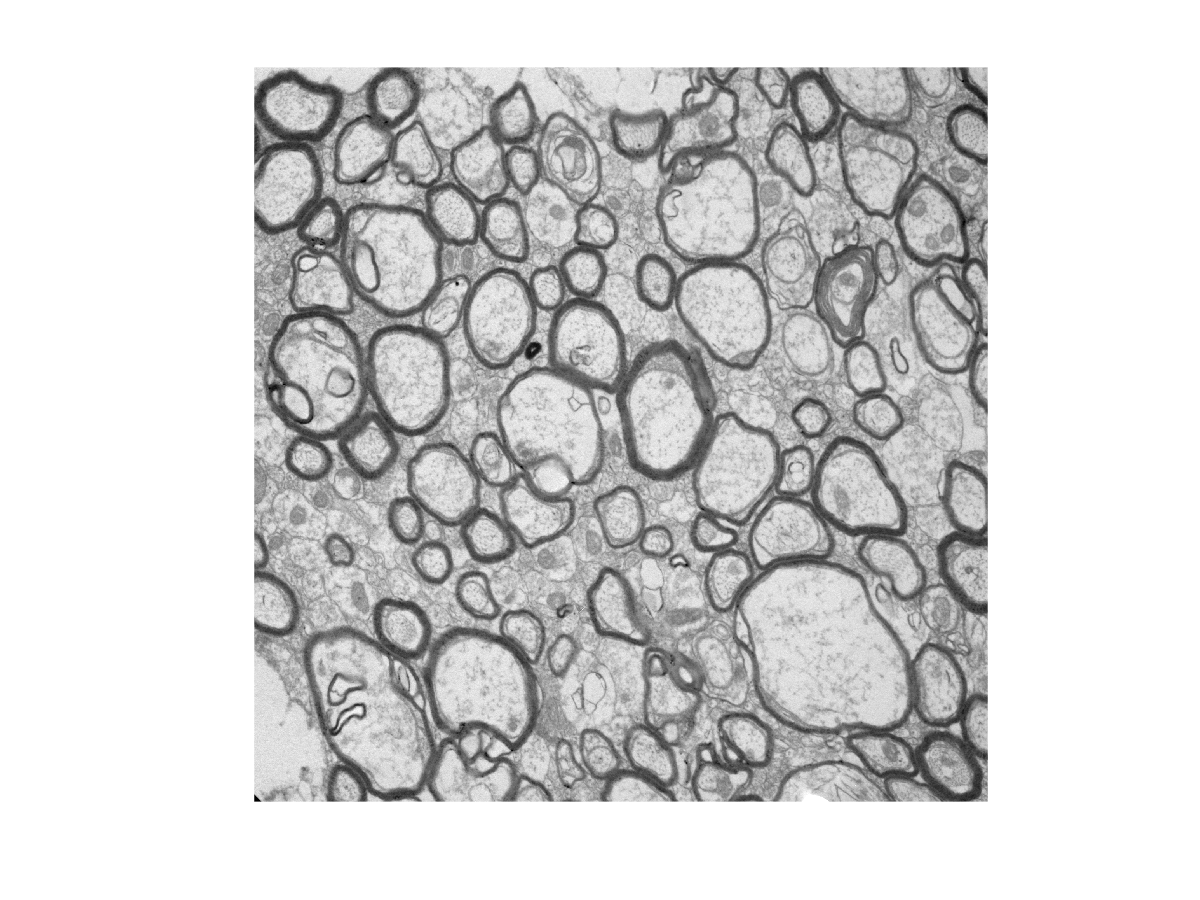

Supplement: Supplementary file 1 — Supplementary material [file mmc1.zip › Histology/Control_1_MidCC_2.tif]

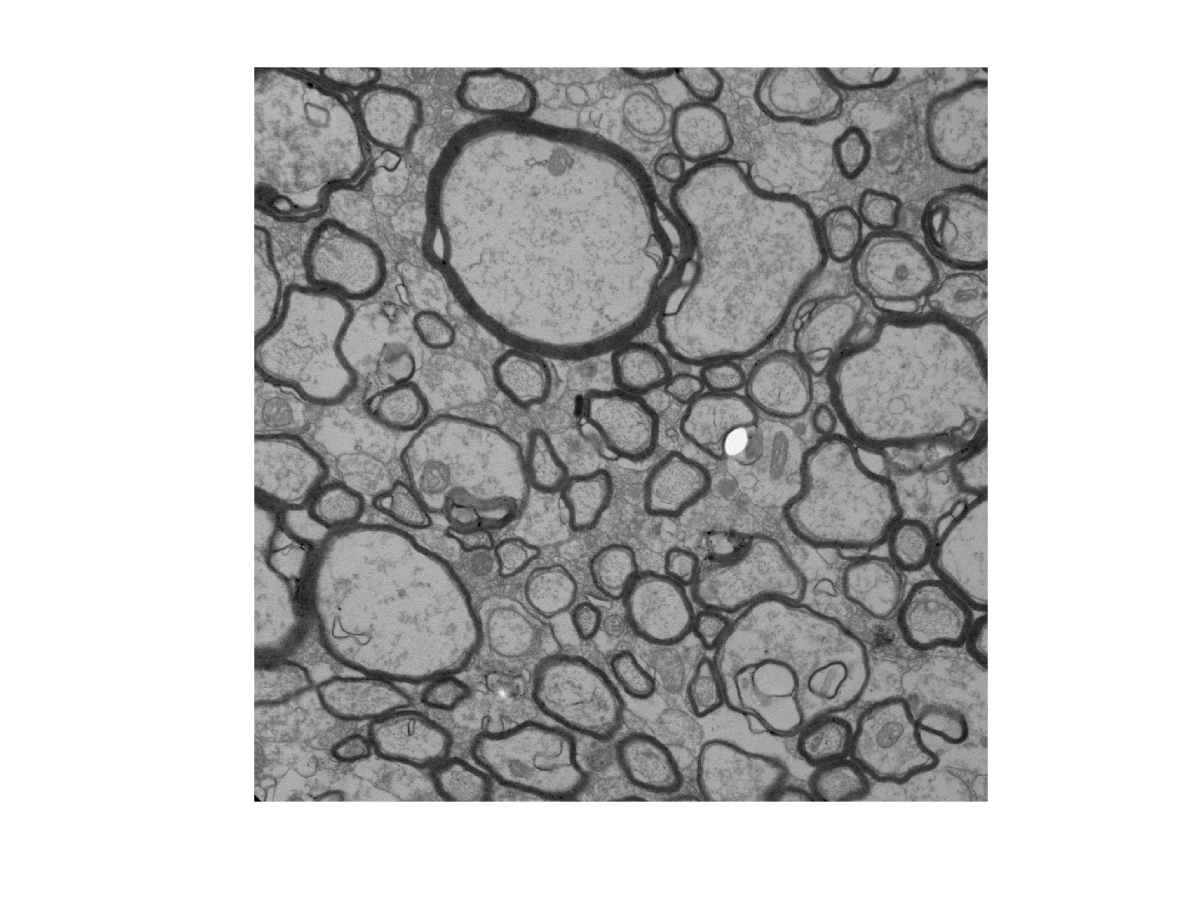

Supplement: Supplementary file 1 — Supplementary material [file mmc1.zip › Histology/Control_1_MidCC_3.tif]

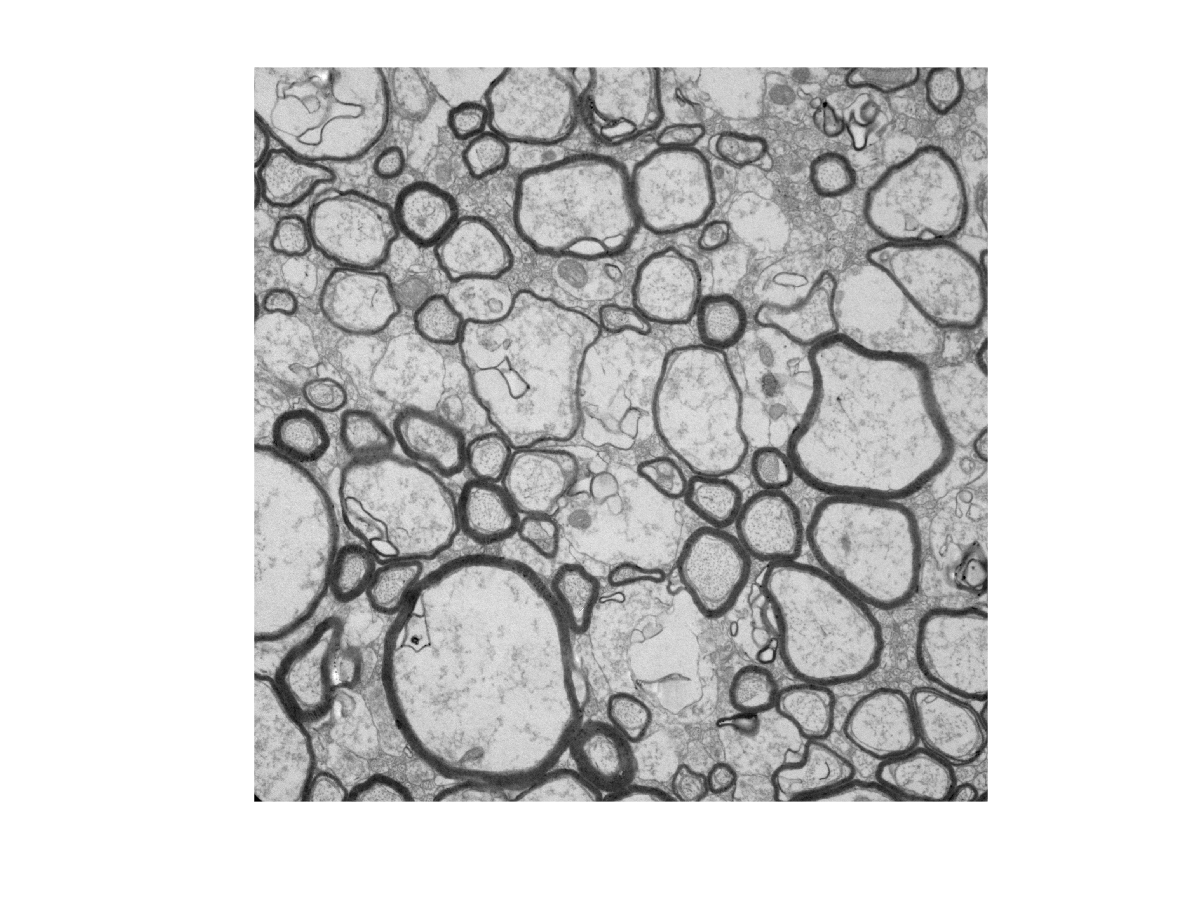

Supplement: Supplementary file 1 — Supplementary material [file mmc1.zip › Histology/Control_1_MidCC_4.tif]

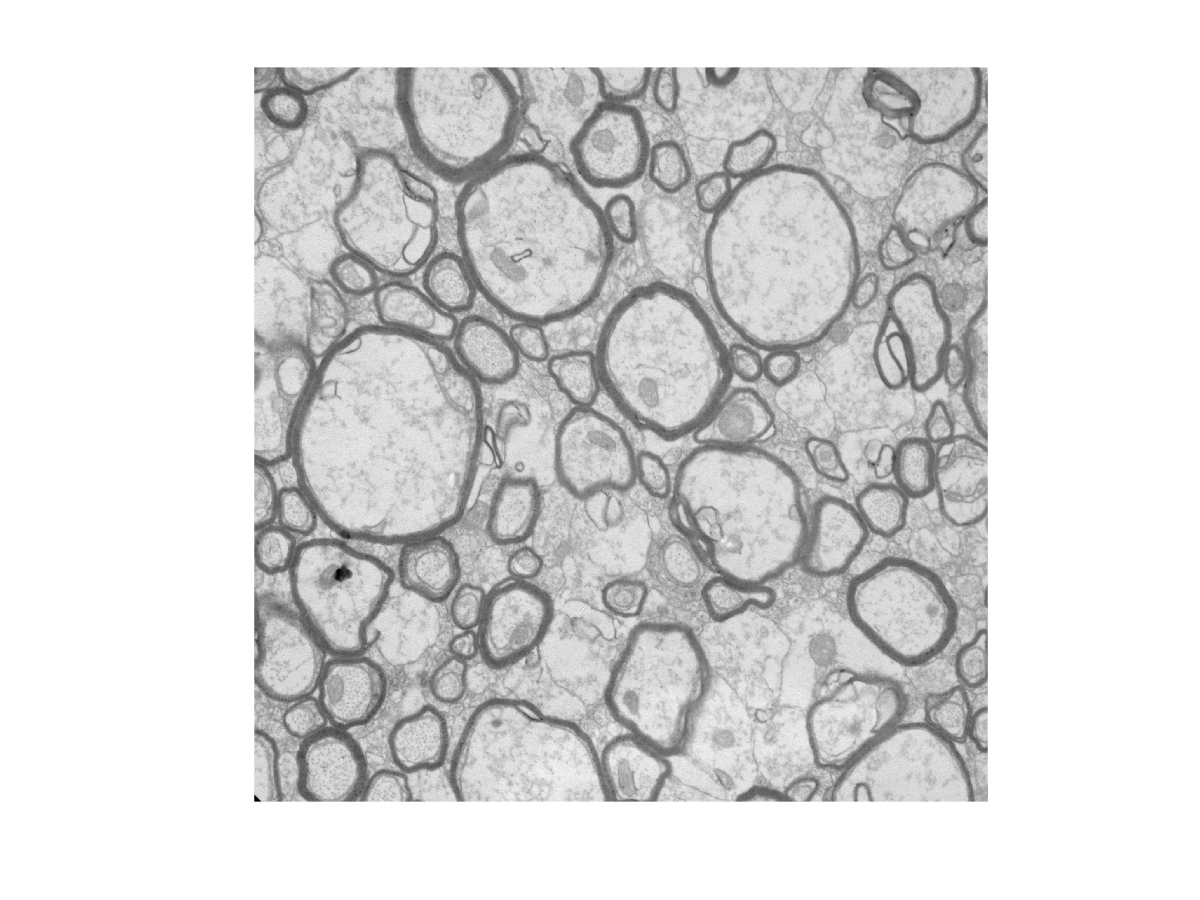

Supplement: Supplementary file 1 — Supplementary material [file mmc1.zip › Histology/Control_1_MidCC_5.tif]

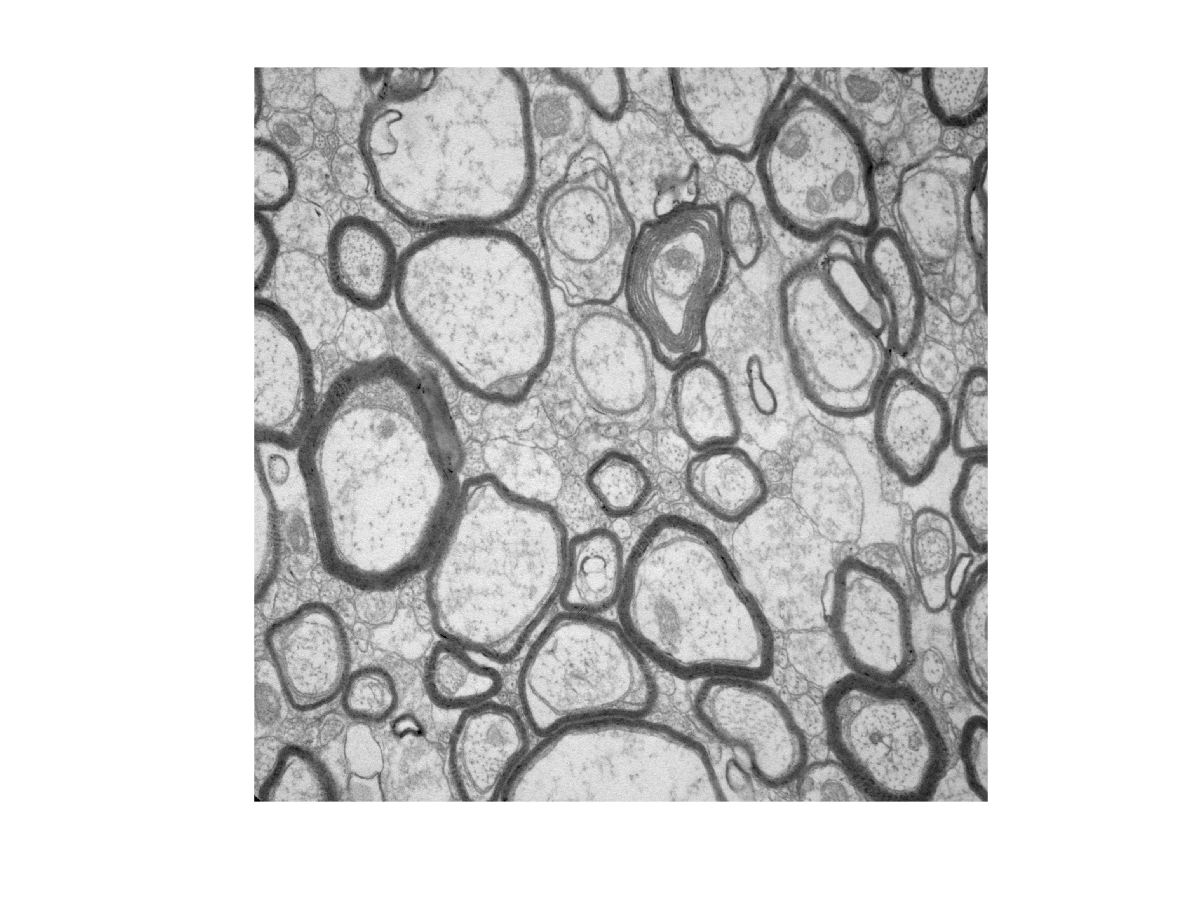

Supplement: Supplementary file 1 — Supplementary material [file mmc1.zip › Histology/Control_1_MidCC_6.tif]

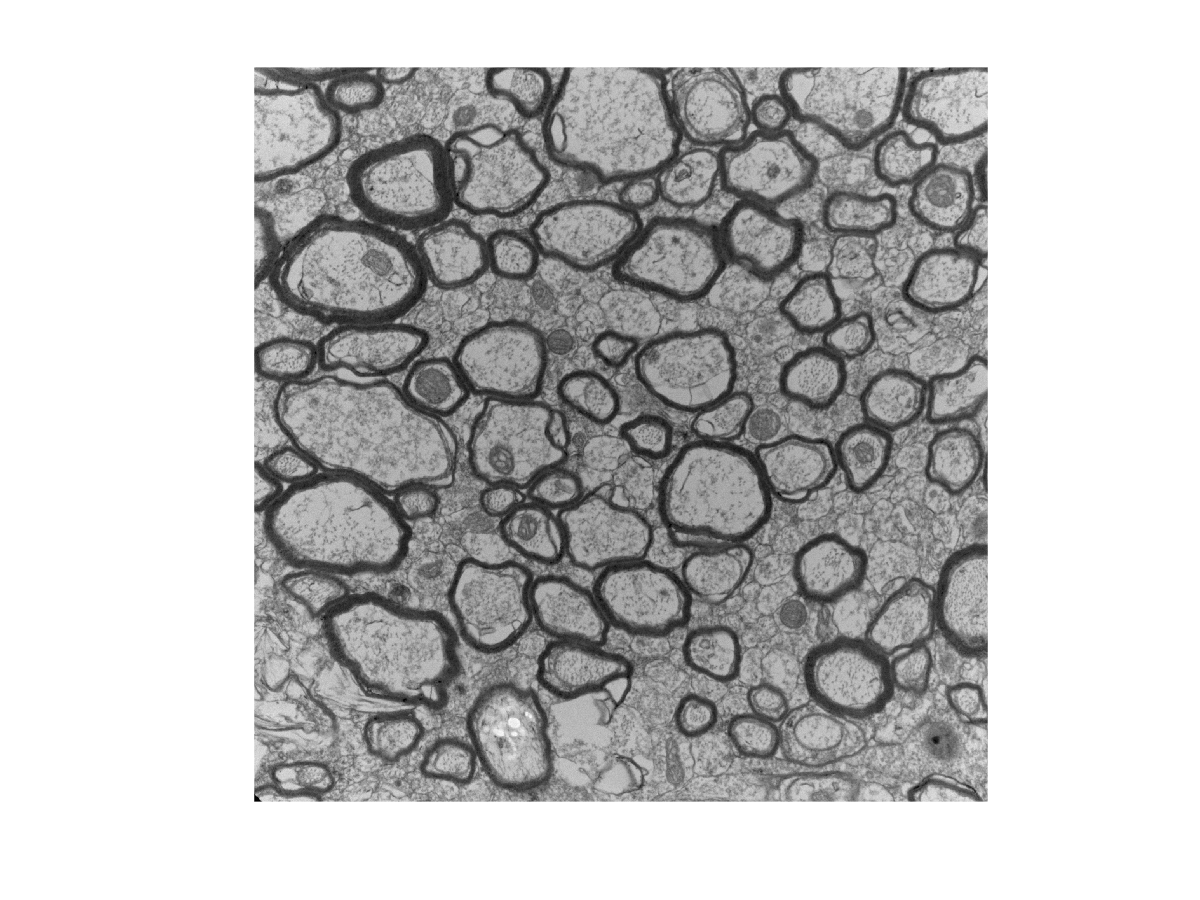

Supplement: Supplementary file 1 — Supplementary material [file mmc1.zip › Histology/Control_2_GCC_1.tif]

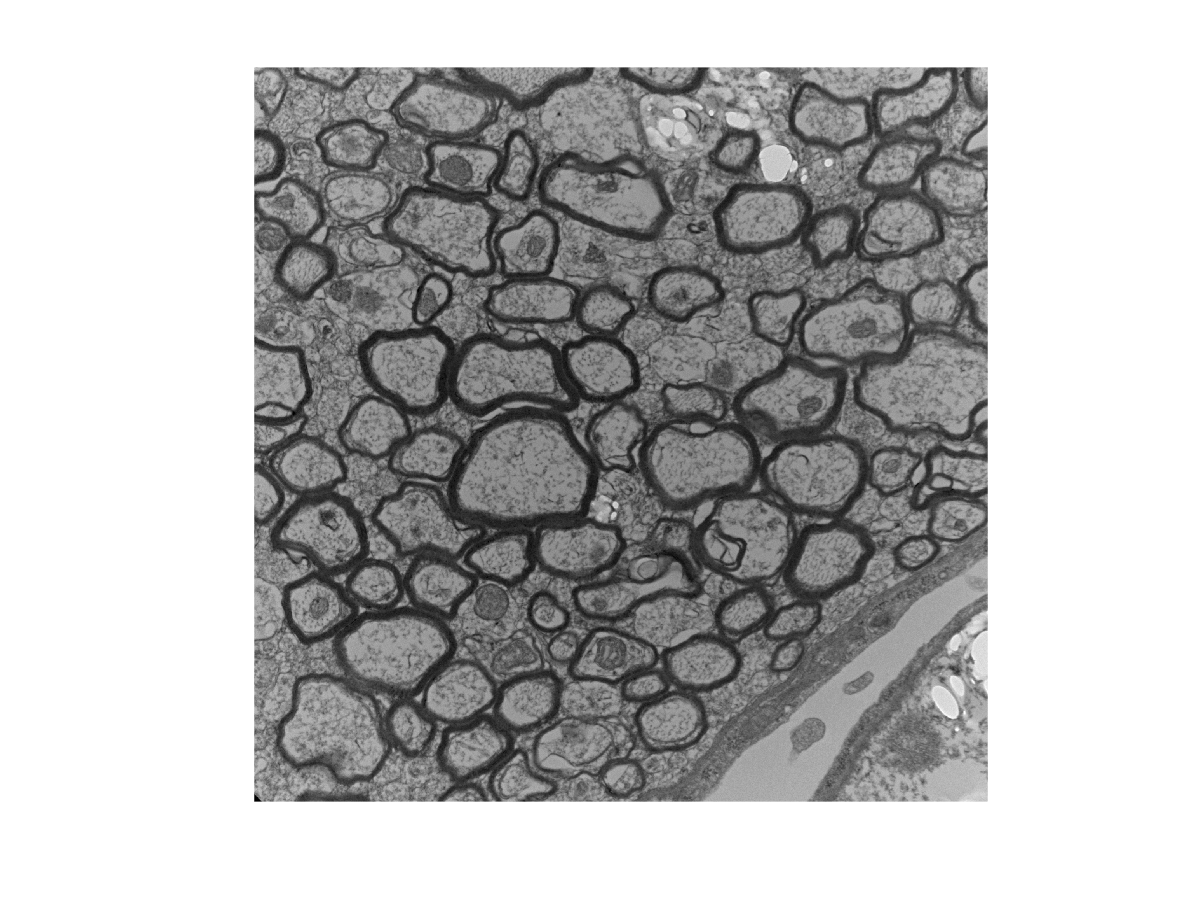

Supplement: Supplementary file 1 — Supplementary material [file mmc1.zip › Histology/Control_2_GCC_2.tif]

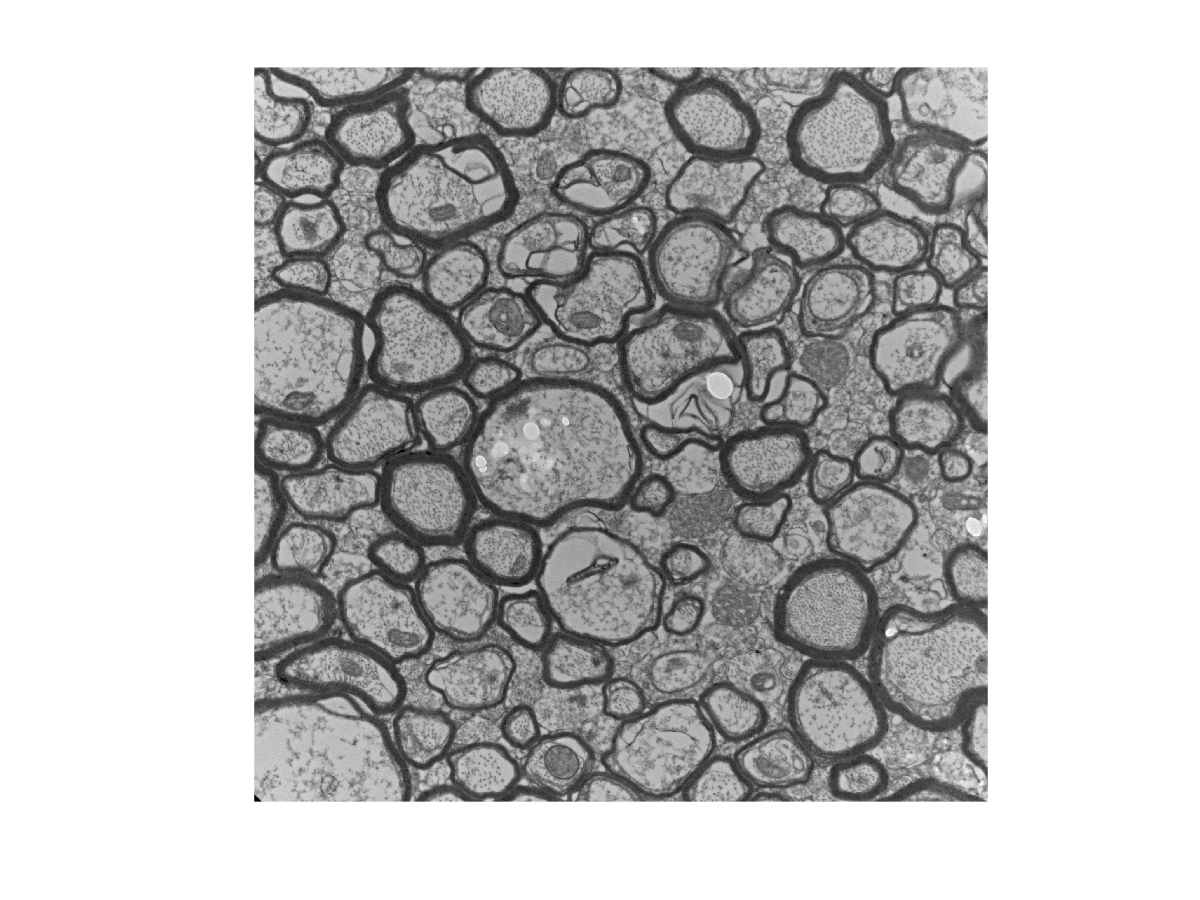

Supplement: Supplementary file 1 — Supplementary material [file mmc1.zip › Histology/Control_2_GCC_3.tif]

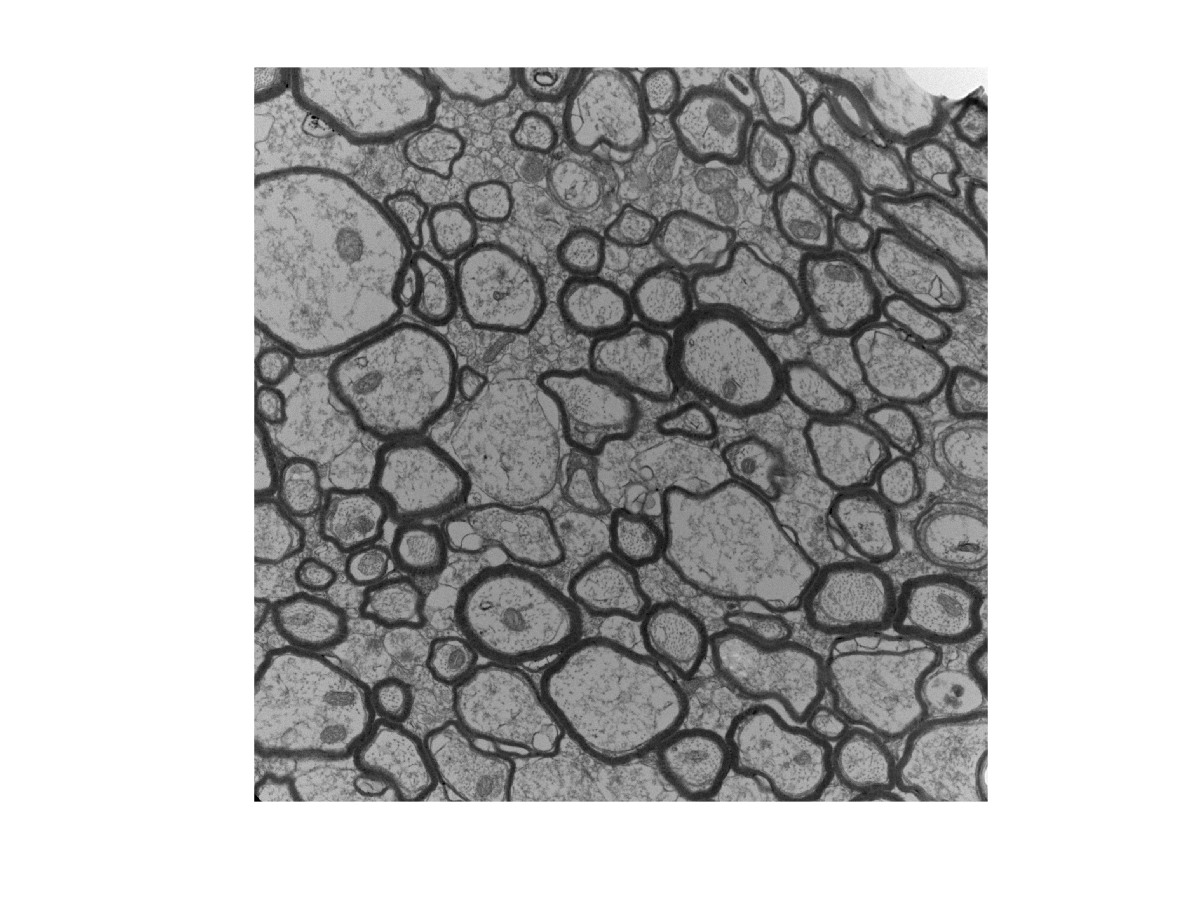

Supplement: Supplementary file 1 — Supplementary material [file mmc1.zip › Histology/Control_2_GCC_4.tif]

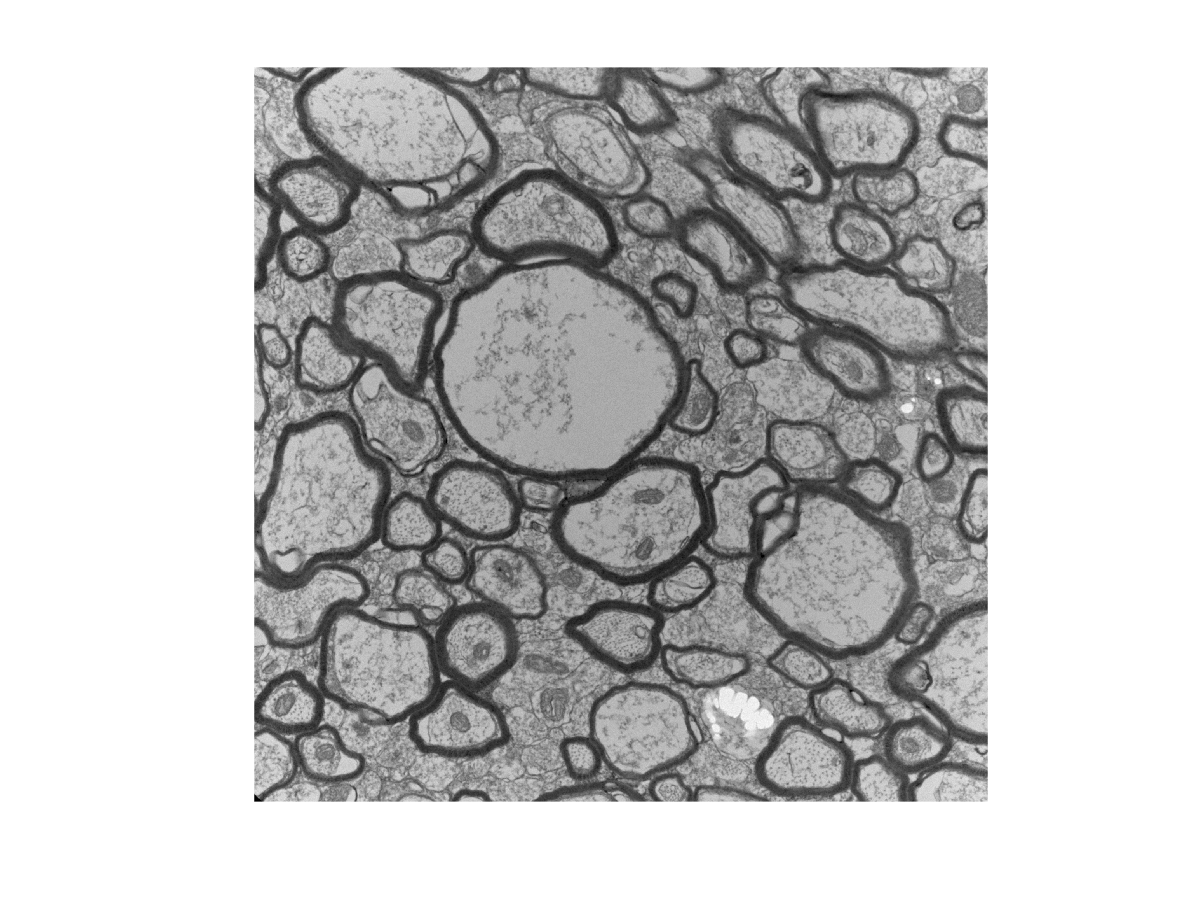

Supplement: Supplementary file 1 — Supplementary material [file mmc1.zip › Histology/Control_2_GCC_5.tif]

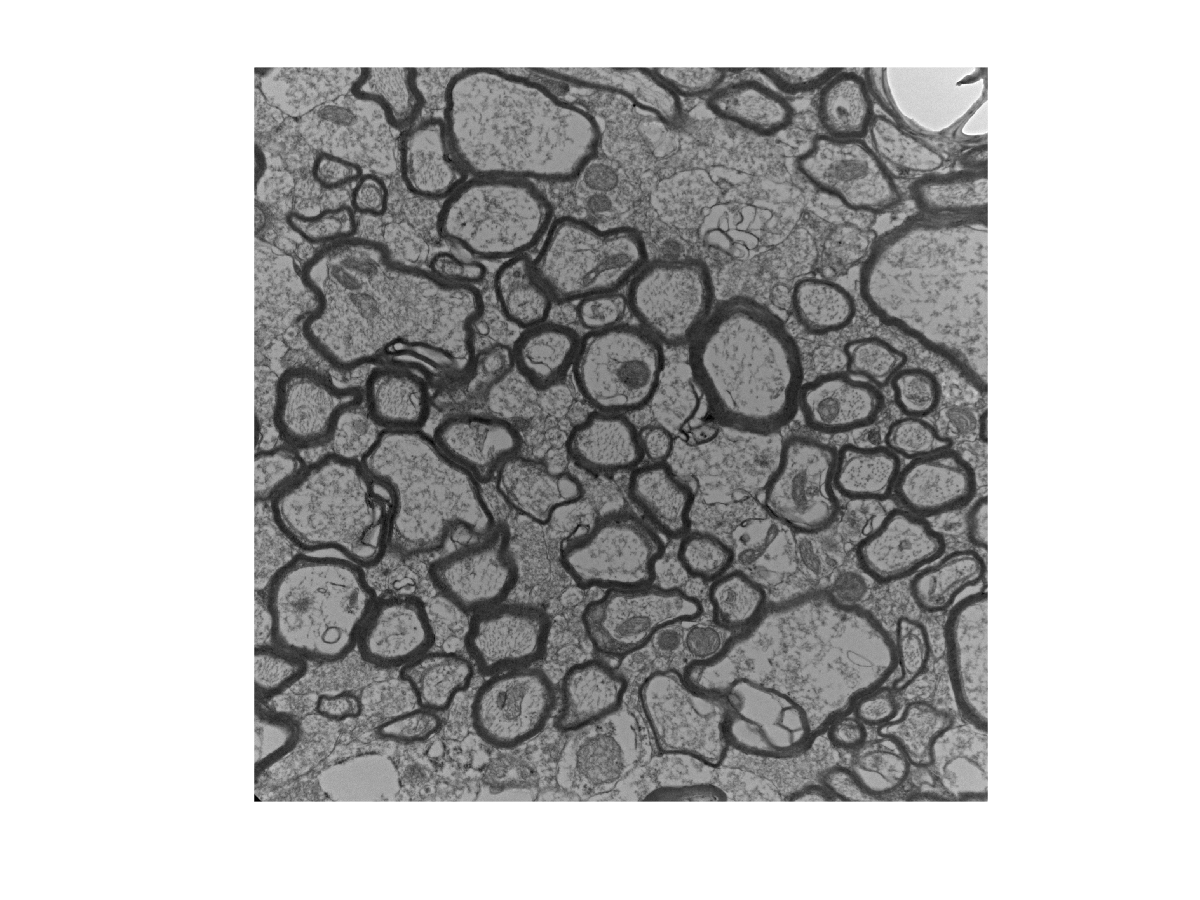

Supplement: Supplementary file 1 — Supplementary material [file mmc1.zip › Histology/Control_2_GCC_6.tif]

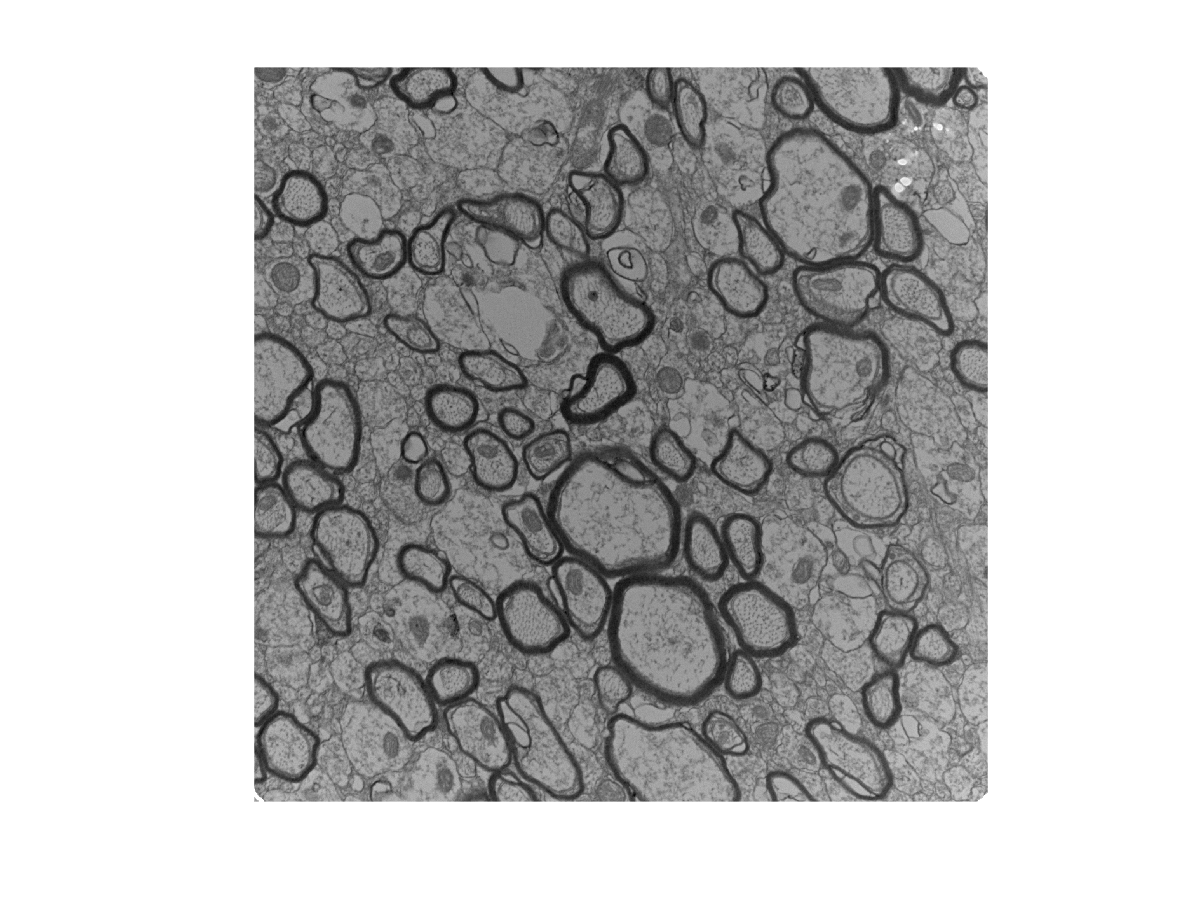

Supplement: Supplementary file 1 — Supplementary material [file mmc1.zip › Histology/Control_2_MidCC_1.tif]

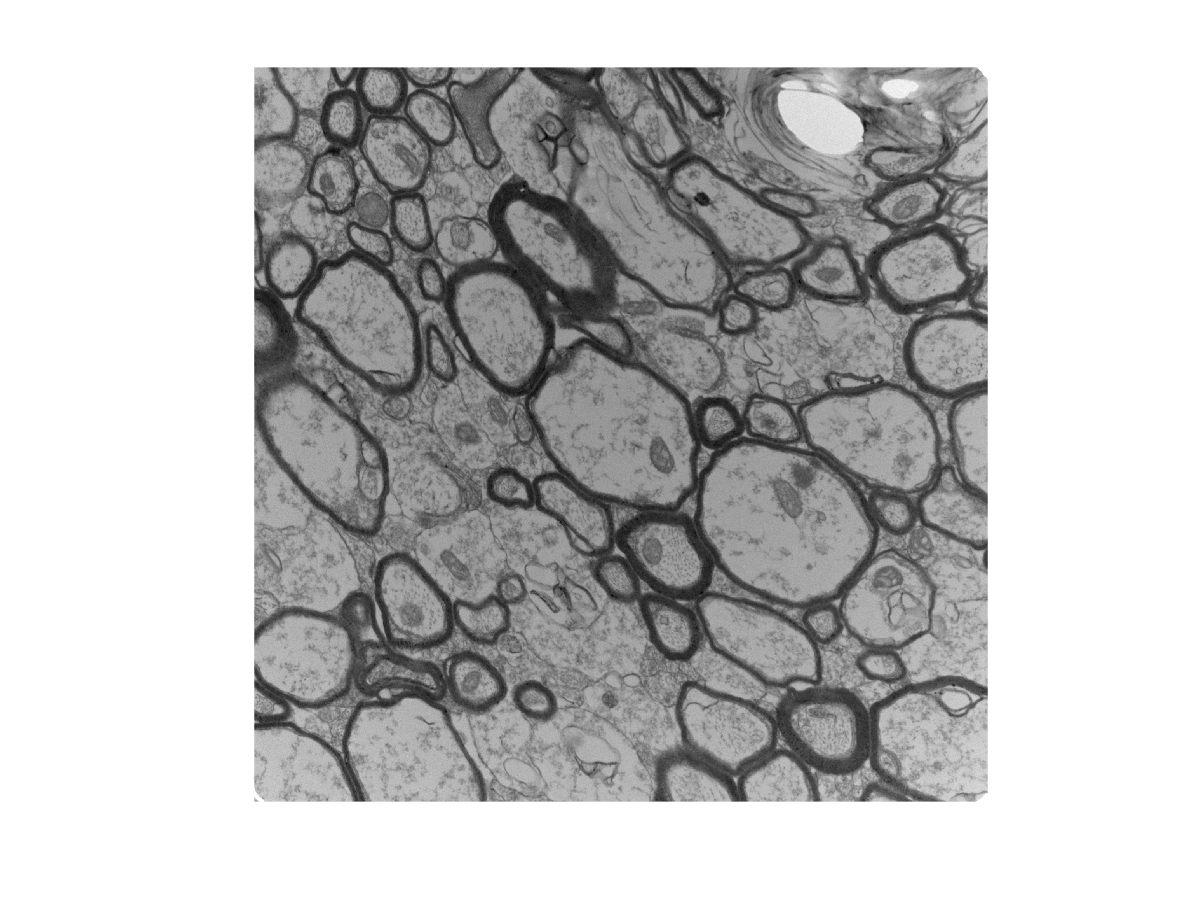

Supplement: Supplementary file 1 — Supplementary material [file mmc1.zip › Histology/Control_2_MidCC_2.tif]

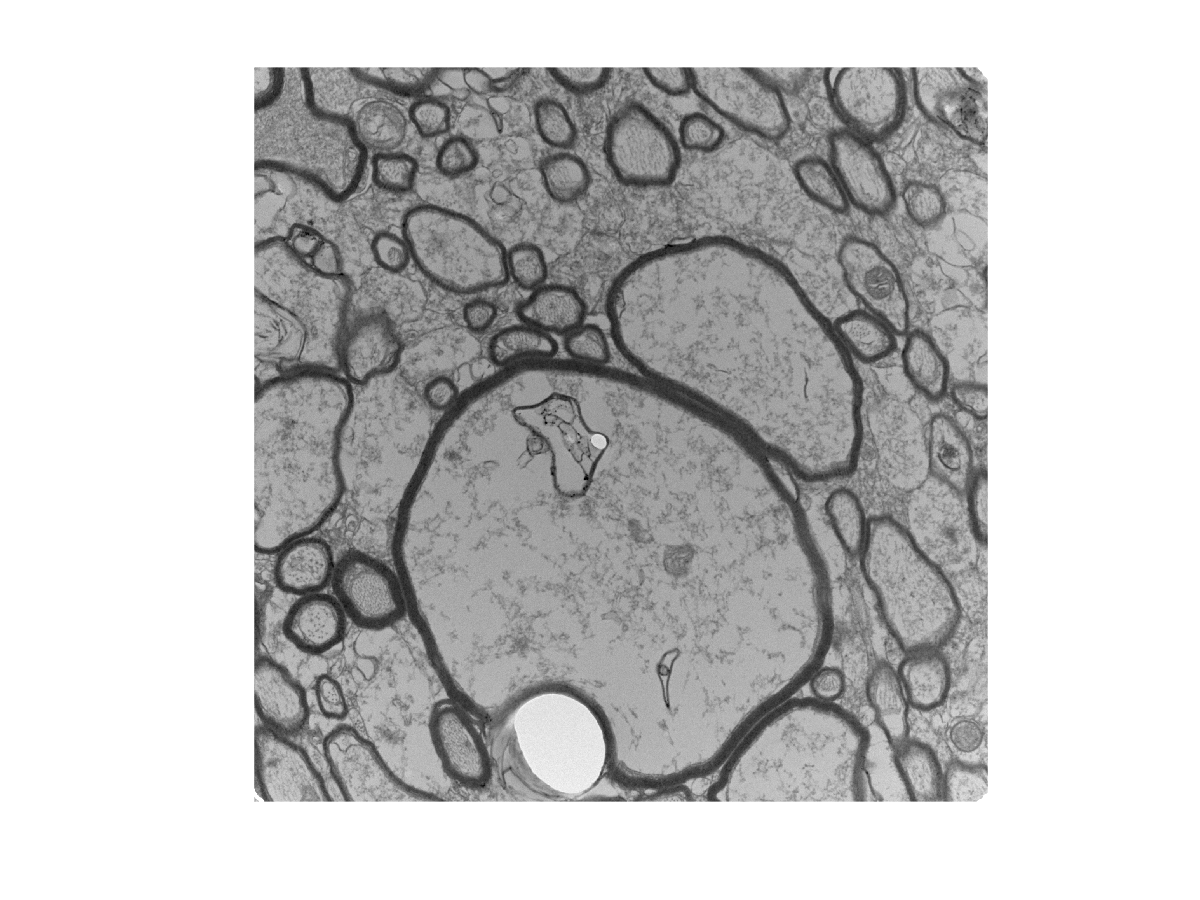

Supplement: Supplementary file 1 — Supplementary material [file mmc1.zip › Histology/Control_2_MidCC_3.tif]

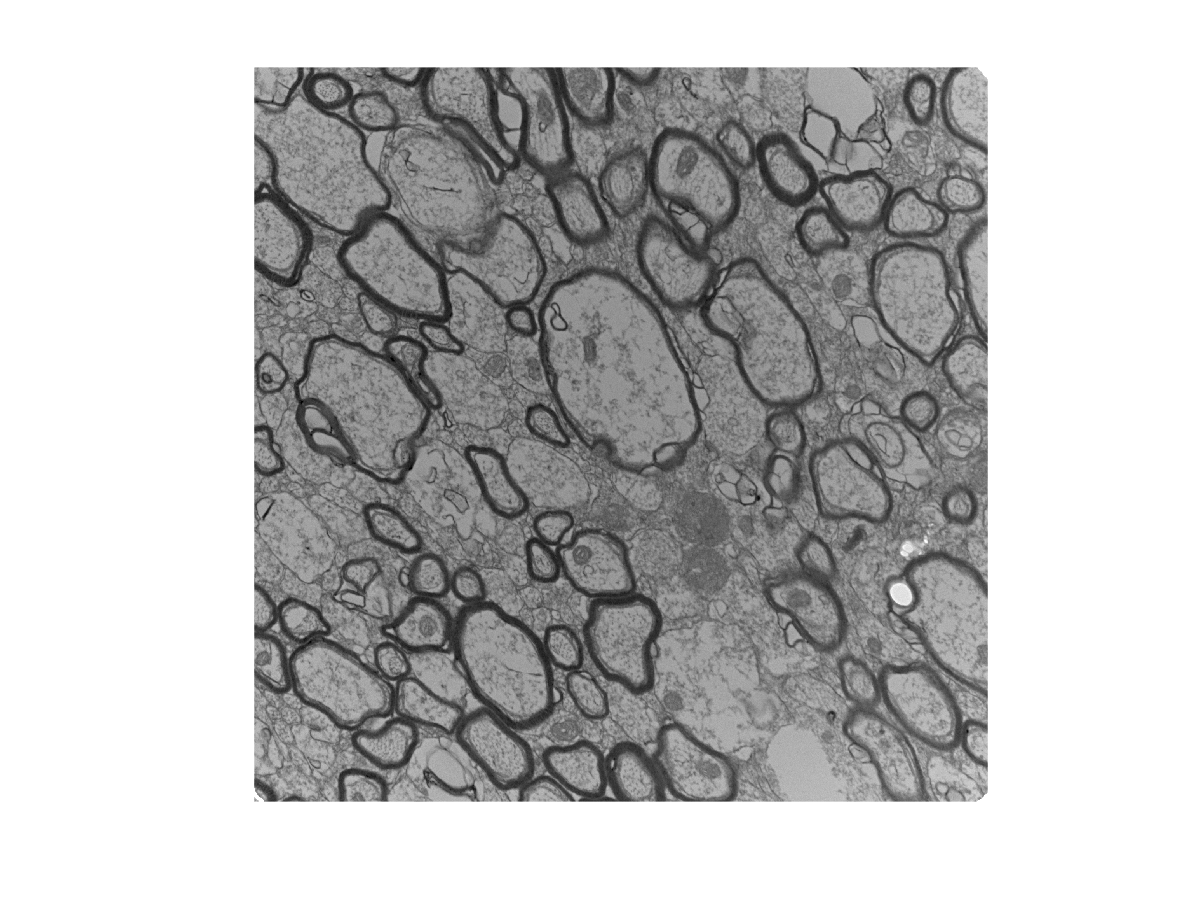

Supplement: Supplementary file 1 — Supplementary material [file mmc1.zip › Histology/Control_2_MidCC_4.tif]

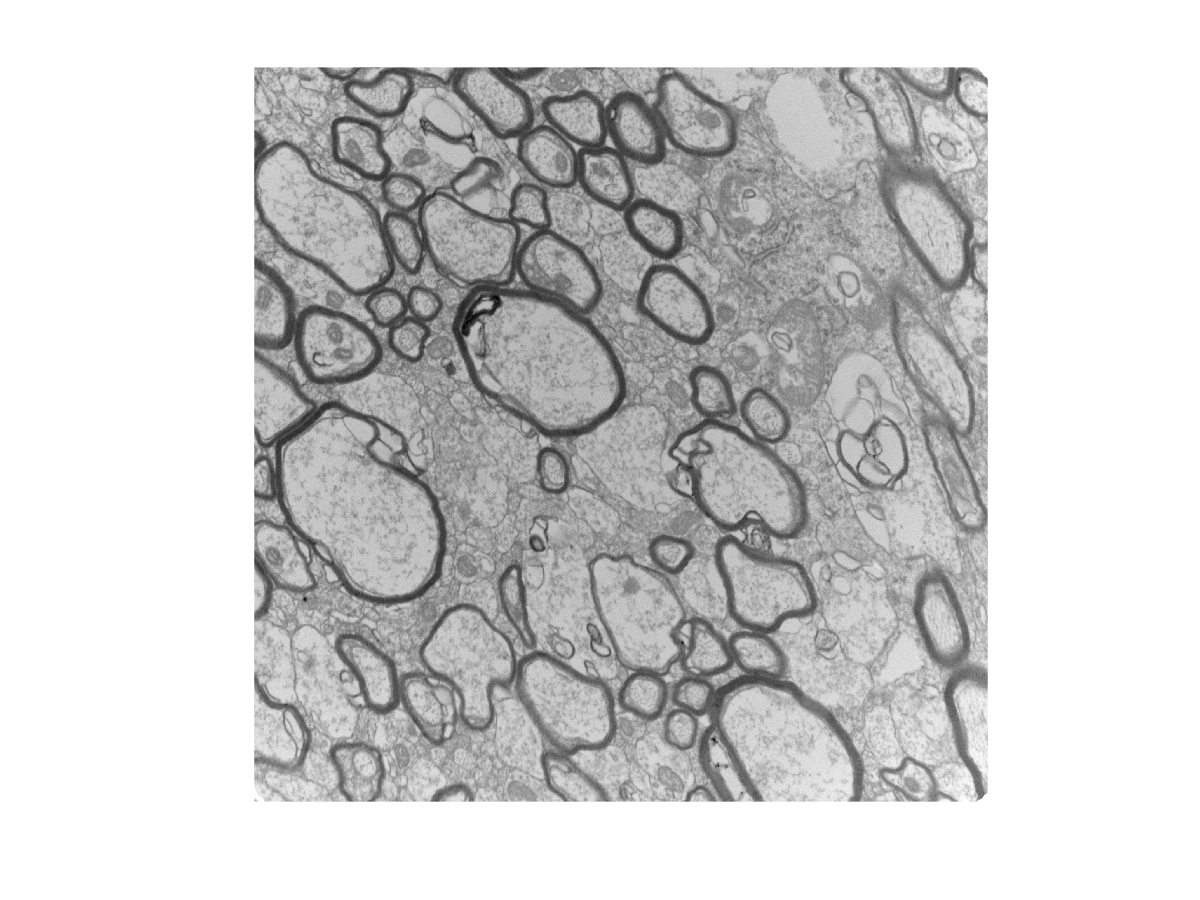

Supplement: Supplementary file 1 — Supplementary material [file mmc1.zip › Histology/Control_2_MidCC_5.tif]

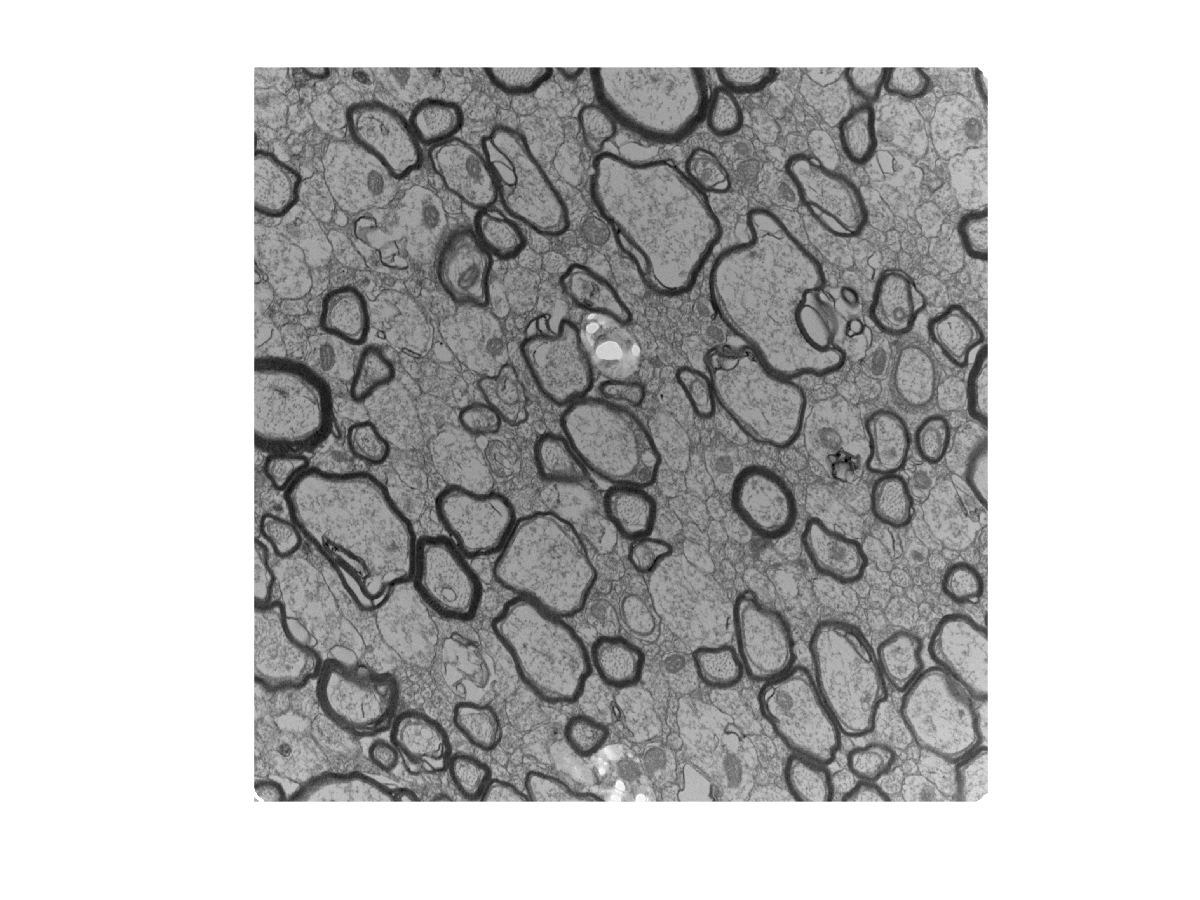

Supplement: Supplementary file 1 — Supplementary material [file mmc1.zip › Histology/Control_2_MidCC_6.tif]

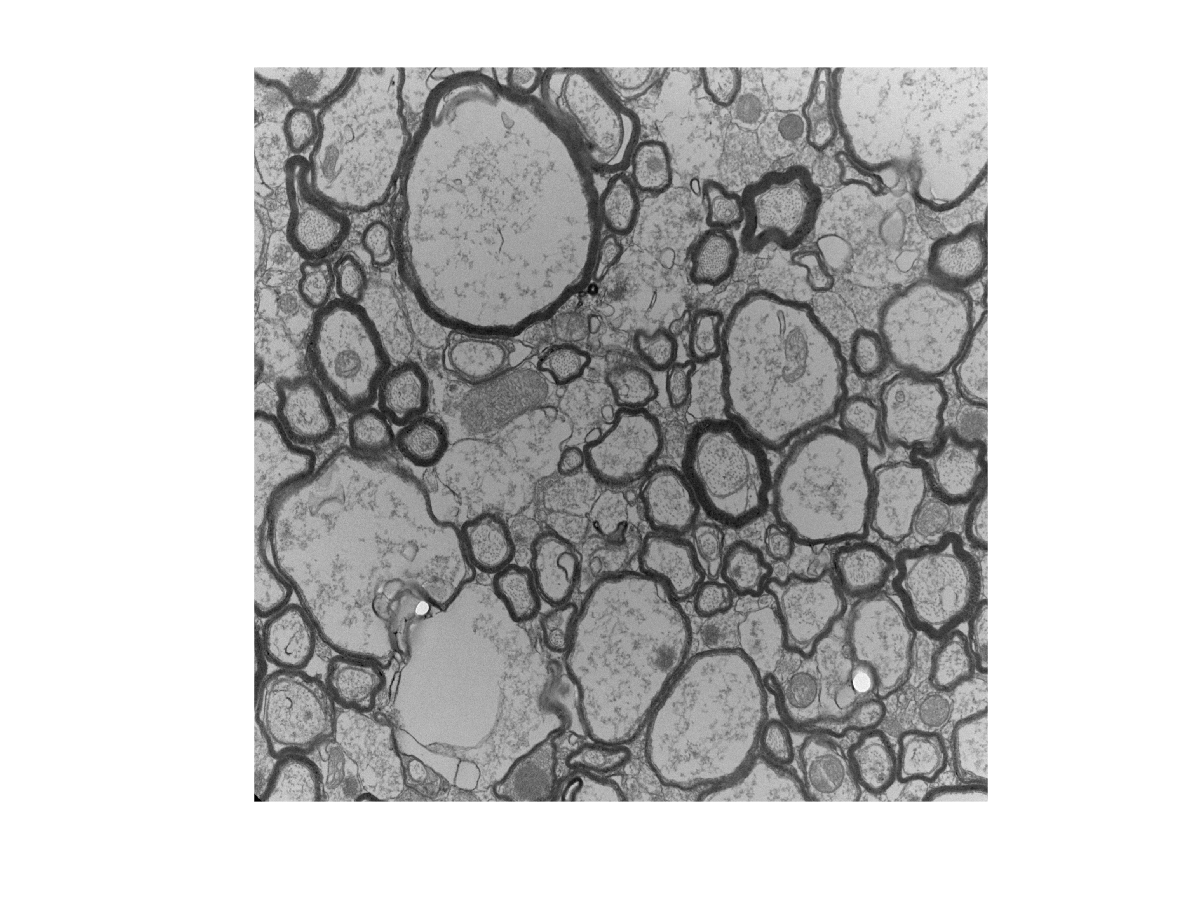

Supplement: Supplementary file 1 — Supplementary material [file mmc1.zip › Histology/Control_3_GCC_1.tif]

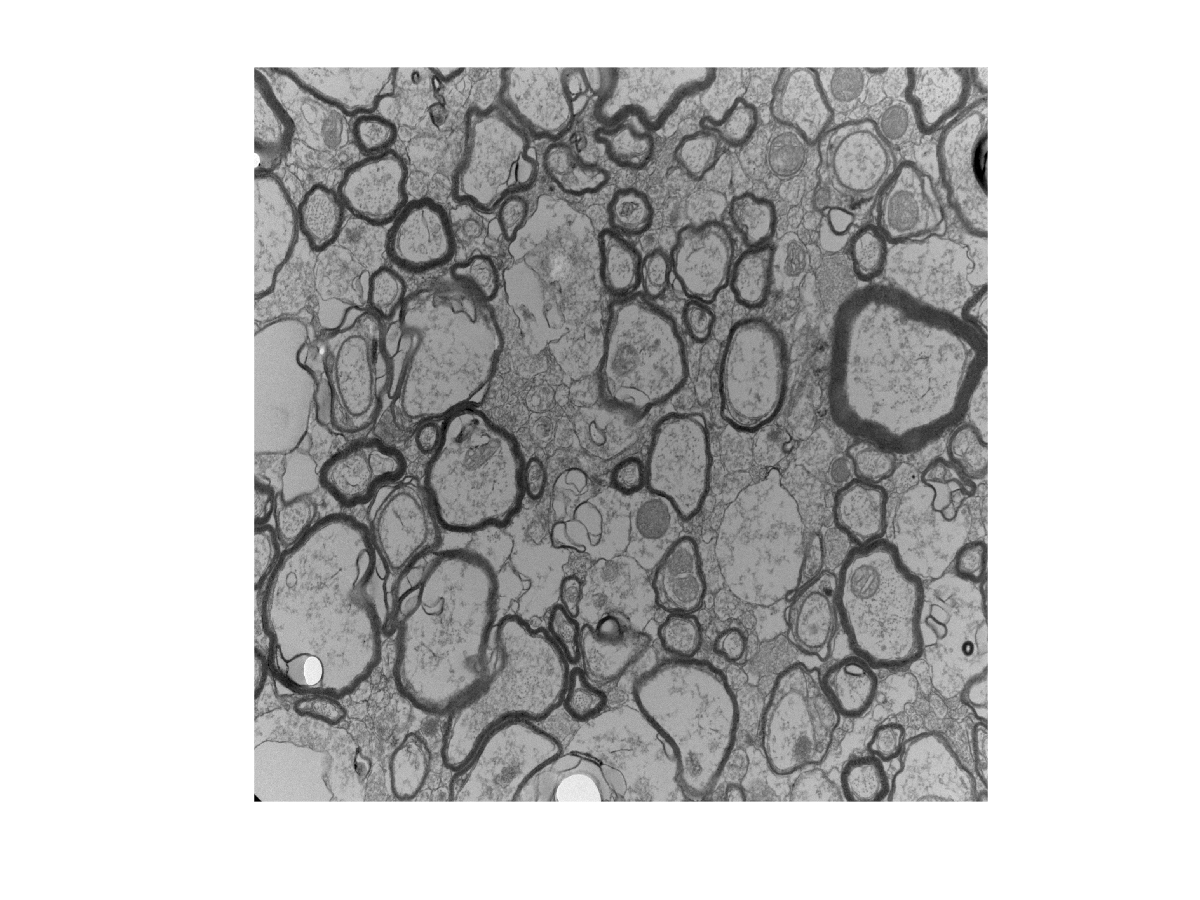

Supplement: Supplementary file 1 — Supplementary material [file mmc1.zip › Histology/Control_3_GCC_2.tif]

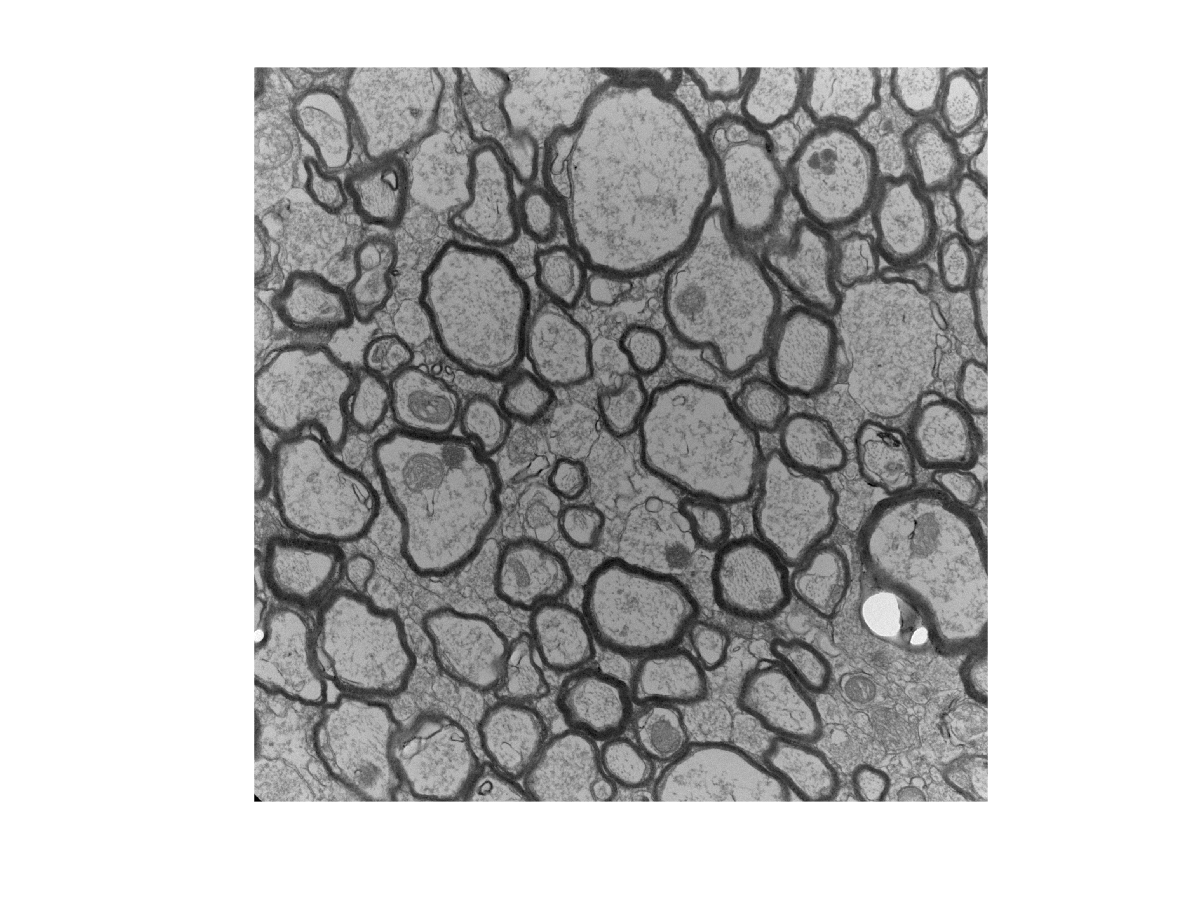

Supplement: Supplementary file 1 — Supplementary material [file mmc1.zip › Histology/Control_3_GCC_3.tif]

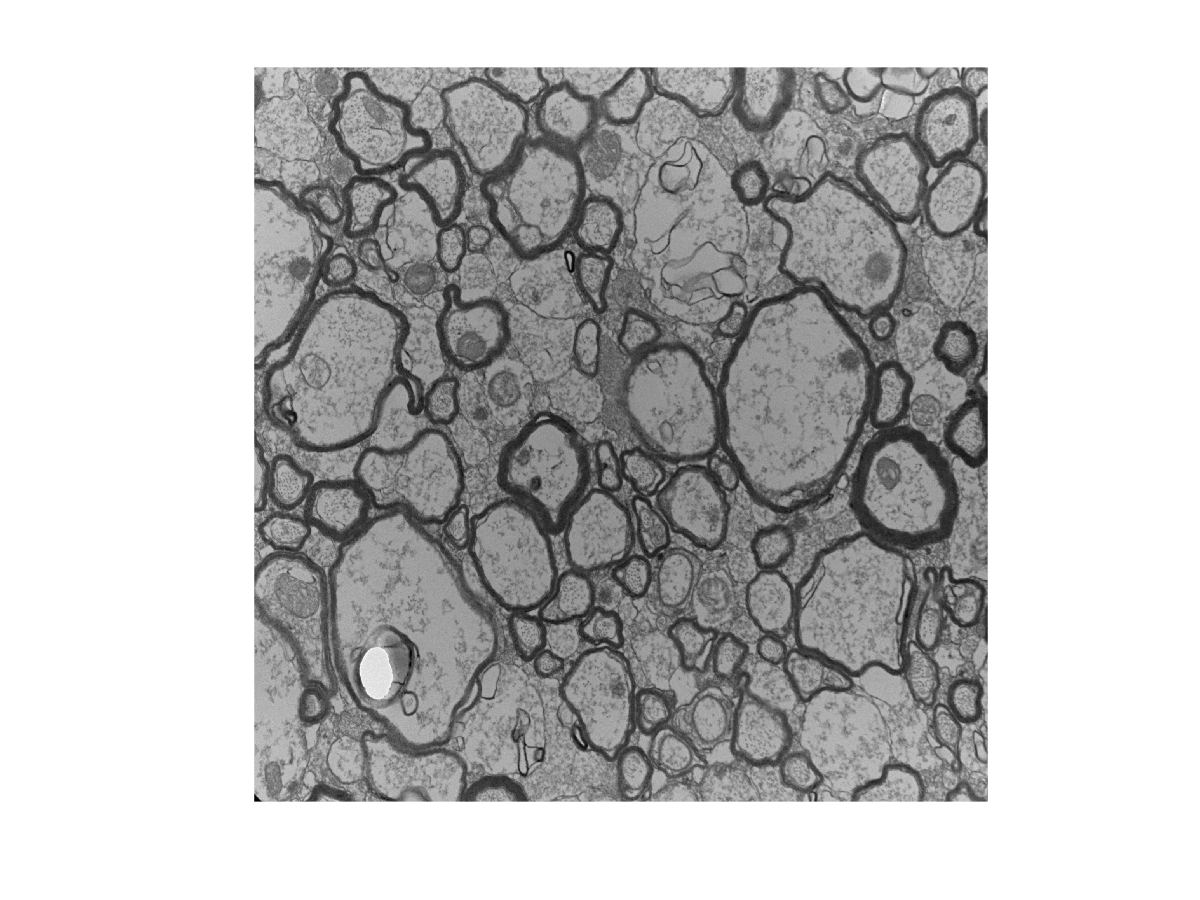

Supplement: Supplementary file 1 — Supplementary material [file mmc1.zip › Histology/Control_3_GCC_4.tif]

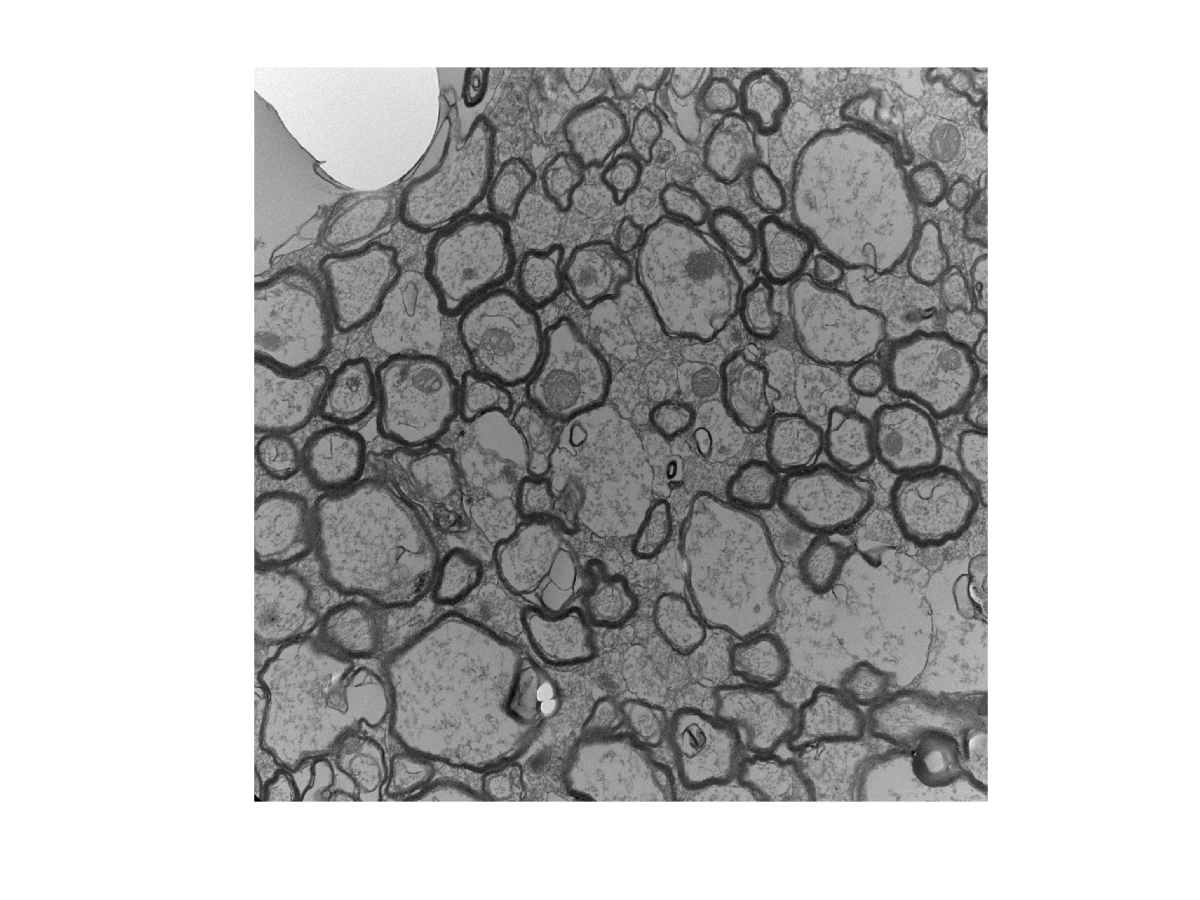

Supplement: Supplementary file 1 — Supplementary material [file mmc1.zip › Histology/Control_3_GCC_5.tif]

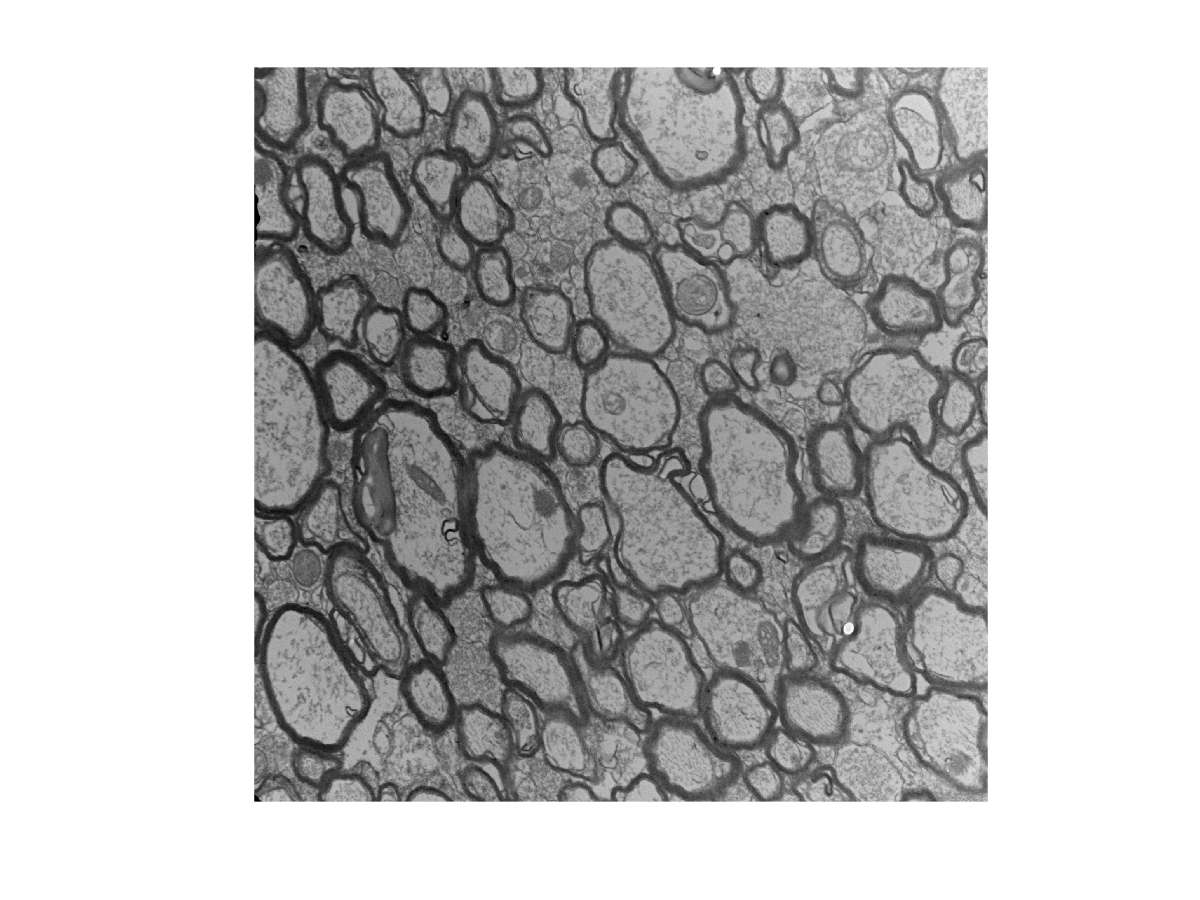

Supplement: Supplementary file 1 — Supplementary material [file mmc1.zip › Histology/Control_3_GCC_6.tif]

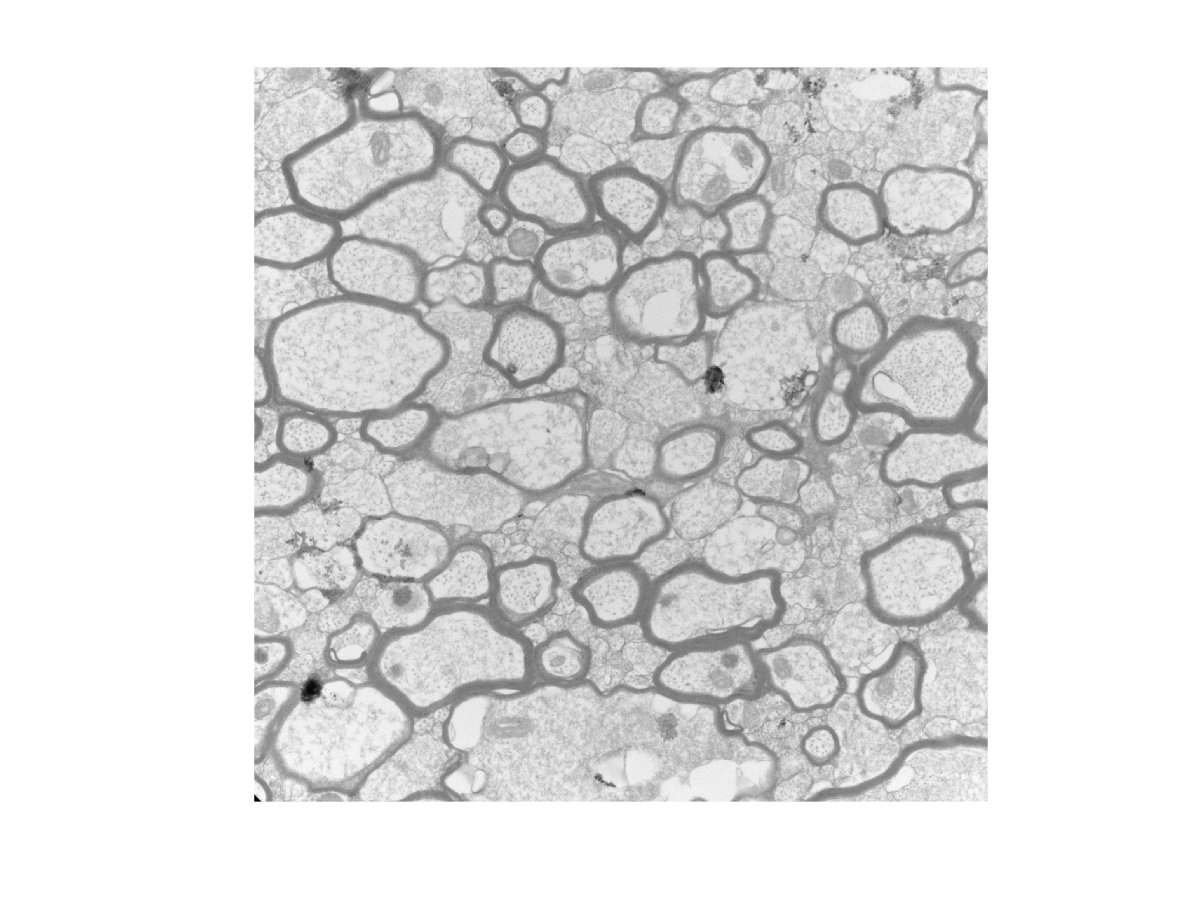

Supplement: Supplementary file 1 — Supplementary material [file mmc1.zip › Histology/Control_3_MidCC_1.tif]

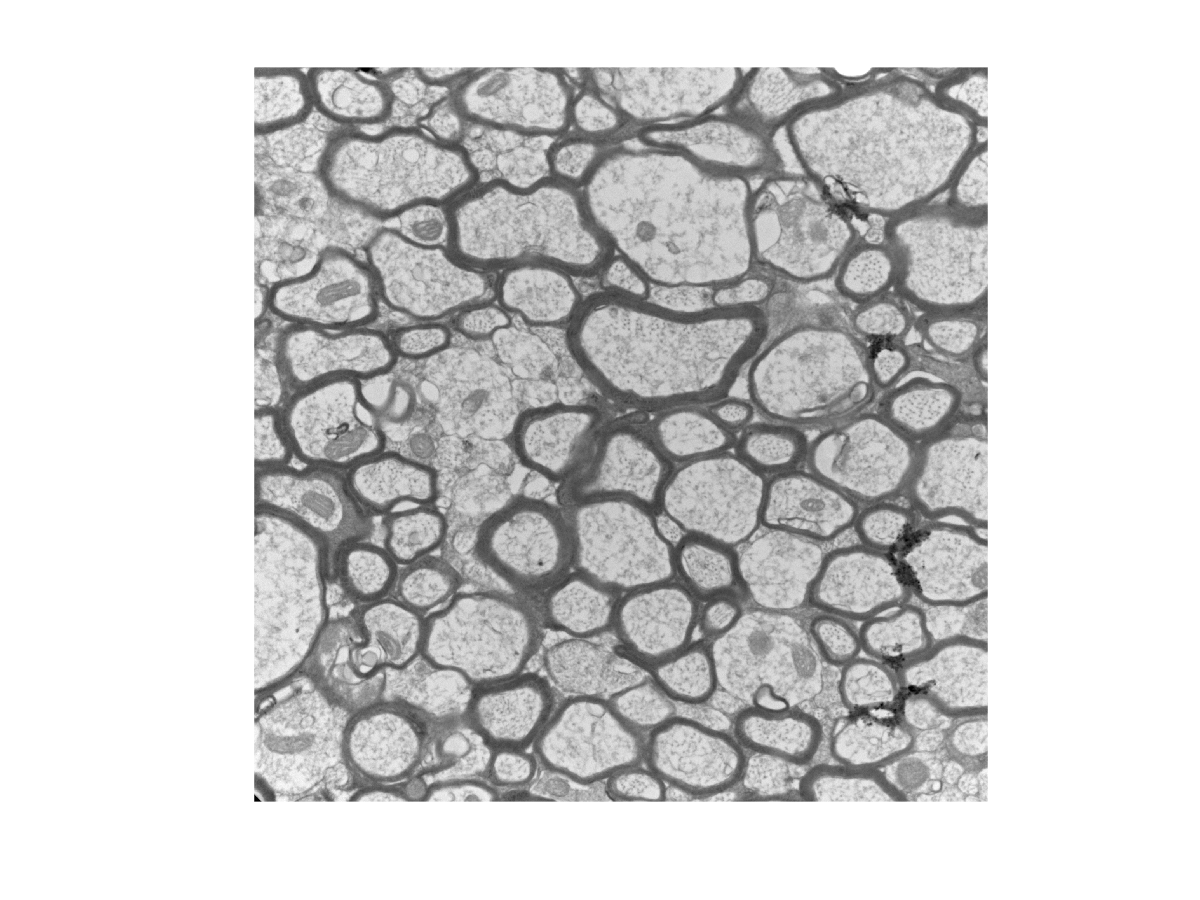

Supplement: Supplementary file 1 — Supplementary material [file mmc1.zip › Histology/Control_3_MidCC_2.tif]

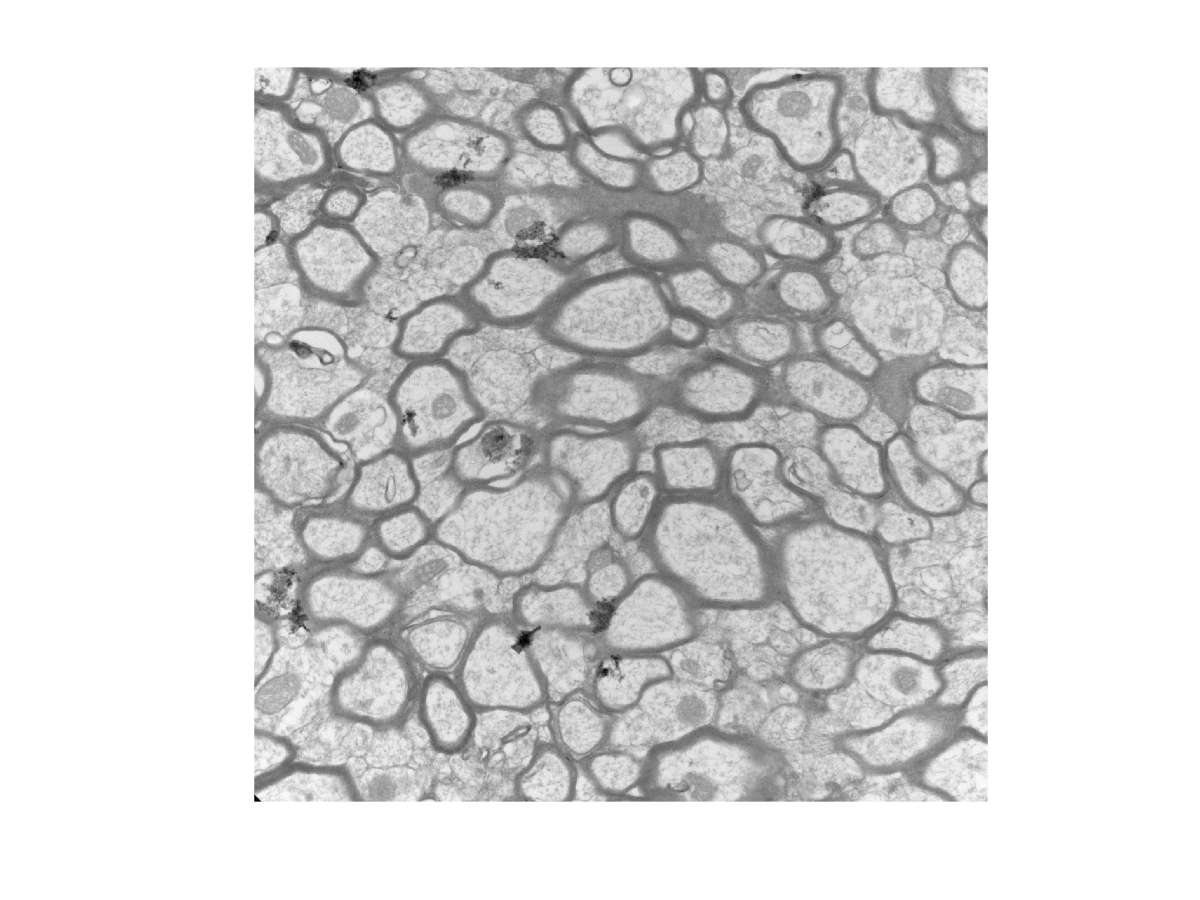

Supplement: Supplementary file 1 — Supplementary material [file mmc1.zip › Histology/Control_3_MidCC_3.tif]

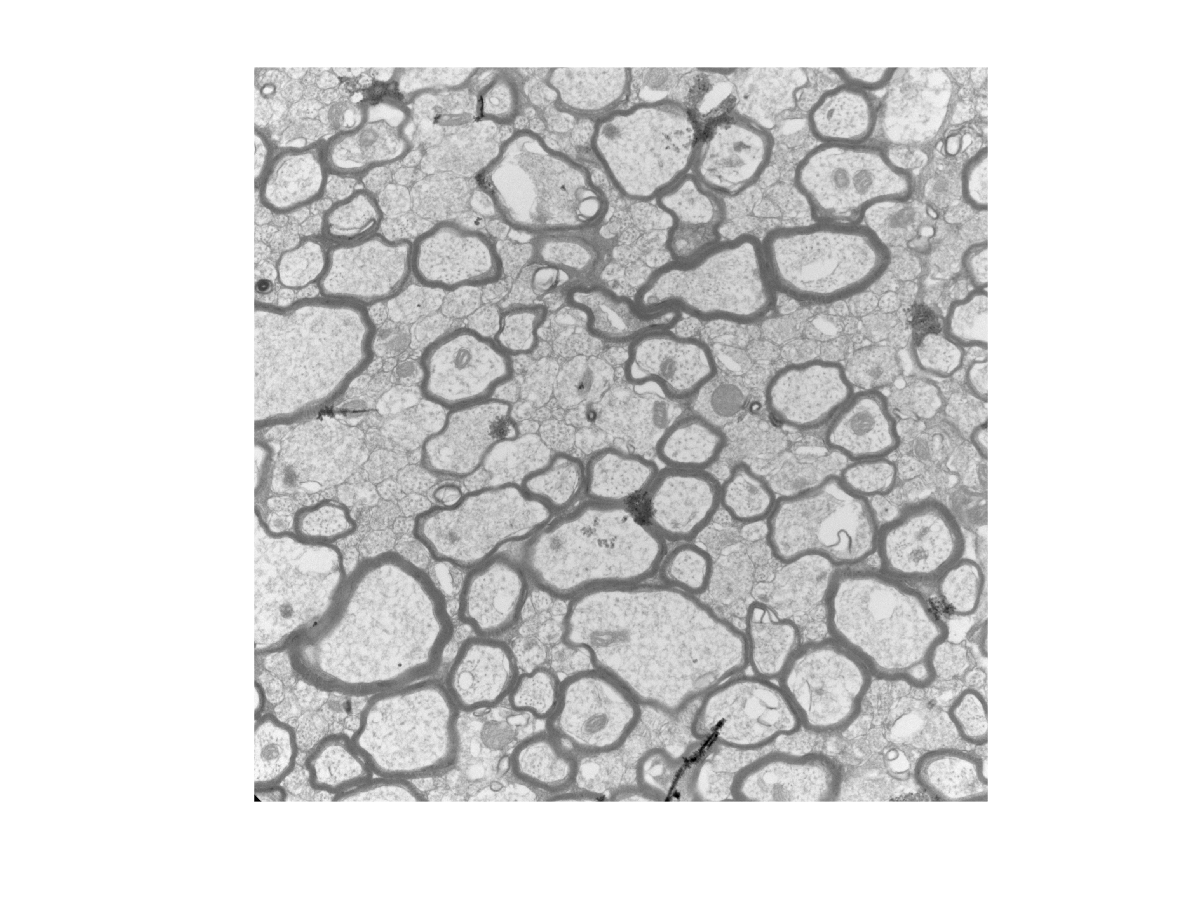

Supplement: Supplementary file 1 — Supplementary material [file mmc1.zip › Histology/Control_3_MidCC_4.tif]

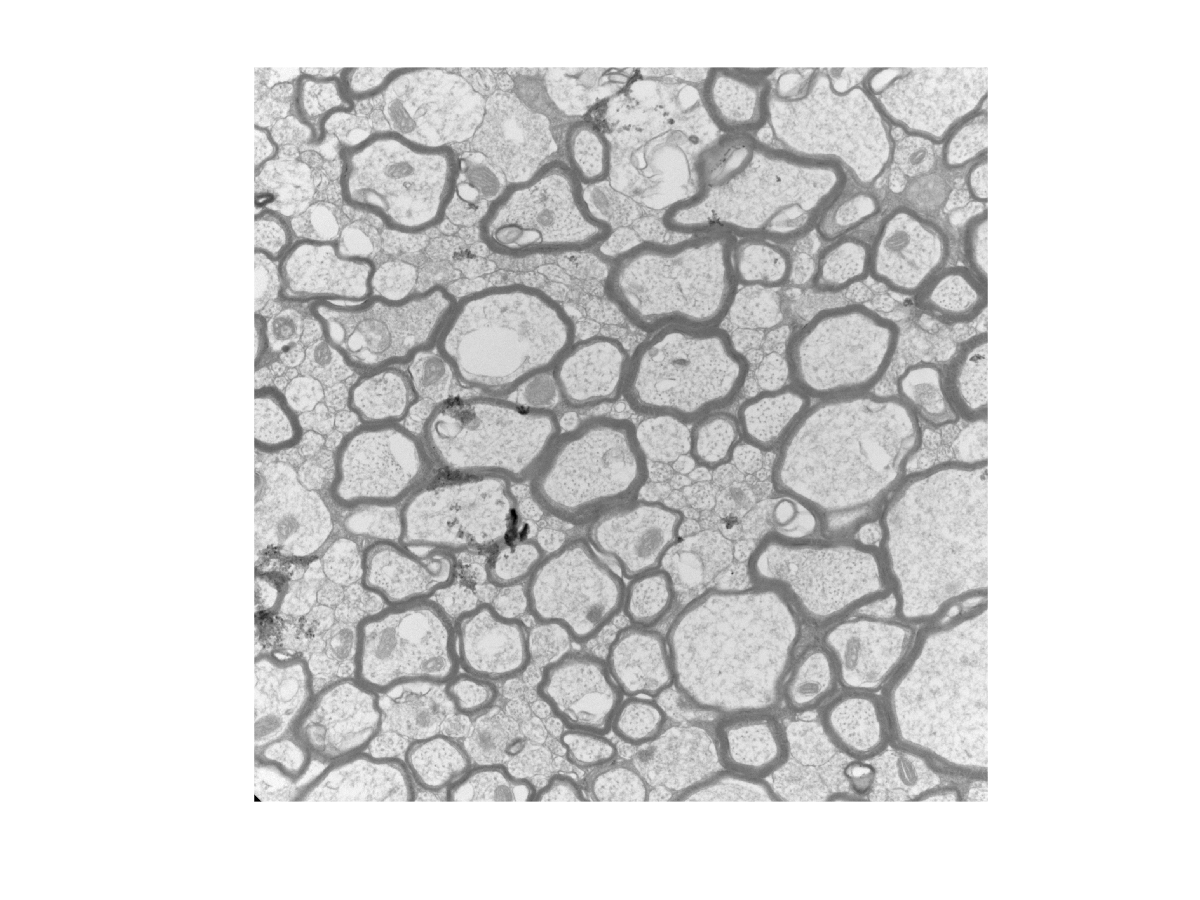

Supplement: Supplementary file 1 — Supplementary material [file mmc1.zip › Histology/Control_3_MidCC_5.tif]

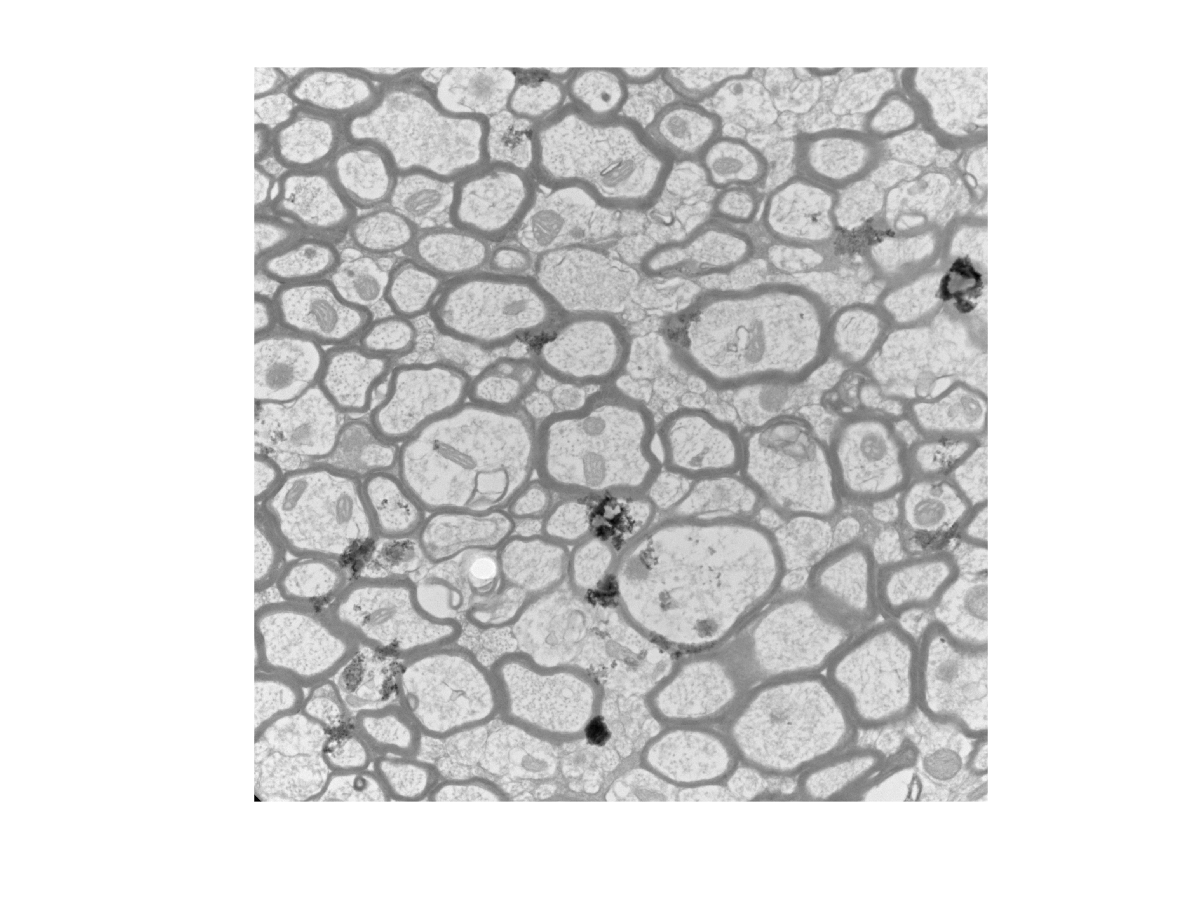

Supplement: Supplementary file 1 — Supplementary material [file mmc1.zip › Histology/Control_3_MidCC_6.tif]

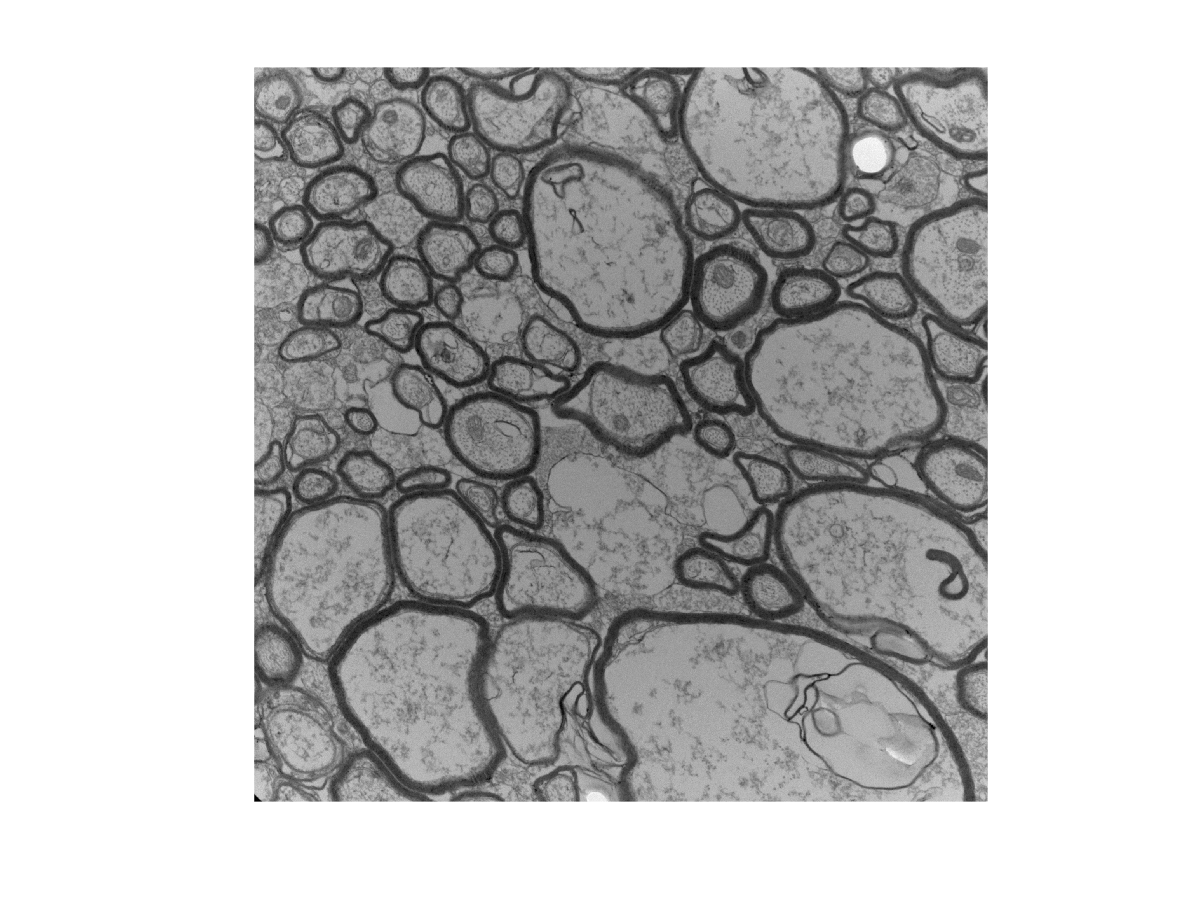

Supplement: Supplementary file 1 — Supplementary material [file mmc1.zip › Histology/Control_4_GCC_1.tif]

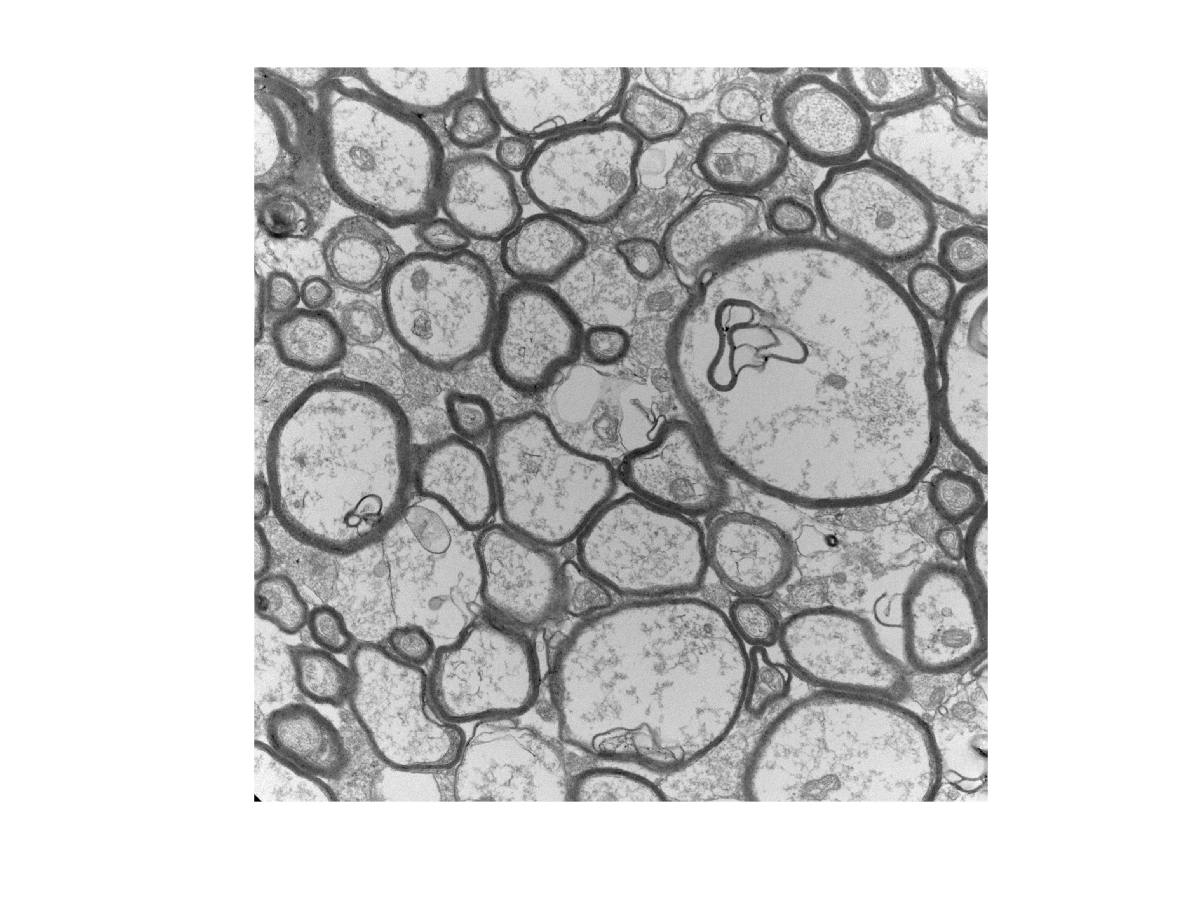

Supplement: Supplementary file 1 — Supplementary material [file mmc1.zip › Histology/Control_4_GCC_2.tif]

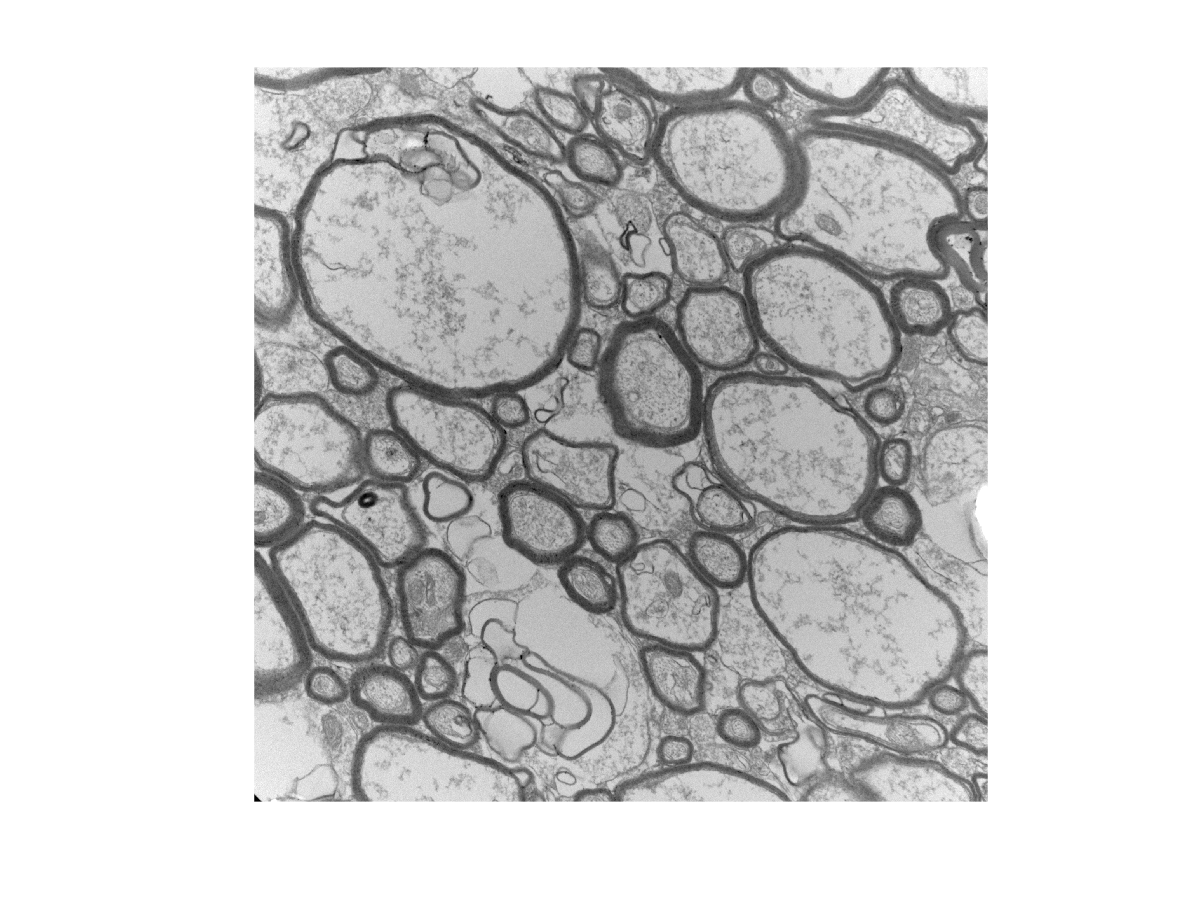

Supplement: Supplementary file 1 — Supplementary material [file mmc1.zip › Histology/Control_4_GCC_3.tif]

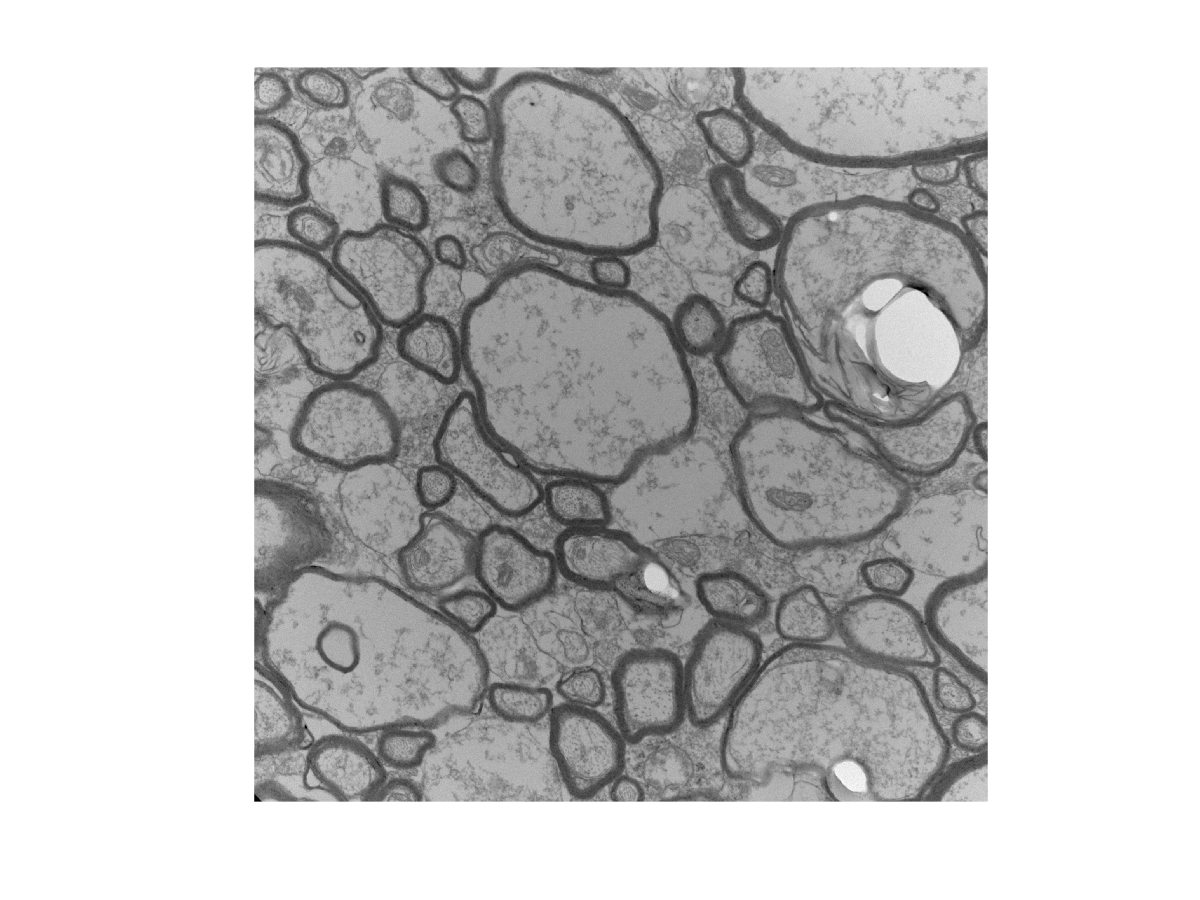

Supplement: Supplementary file 1 — Supplementary material [file mmc1.zip › Histology/Control_4_GCC_4.tif]

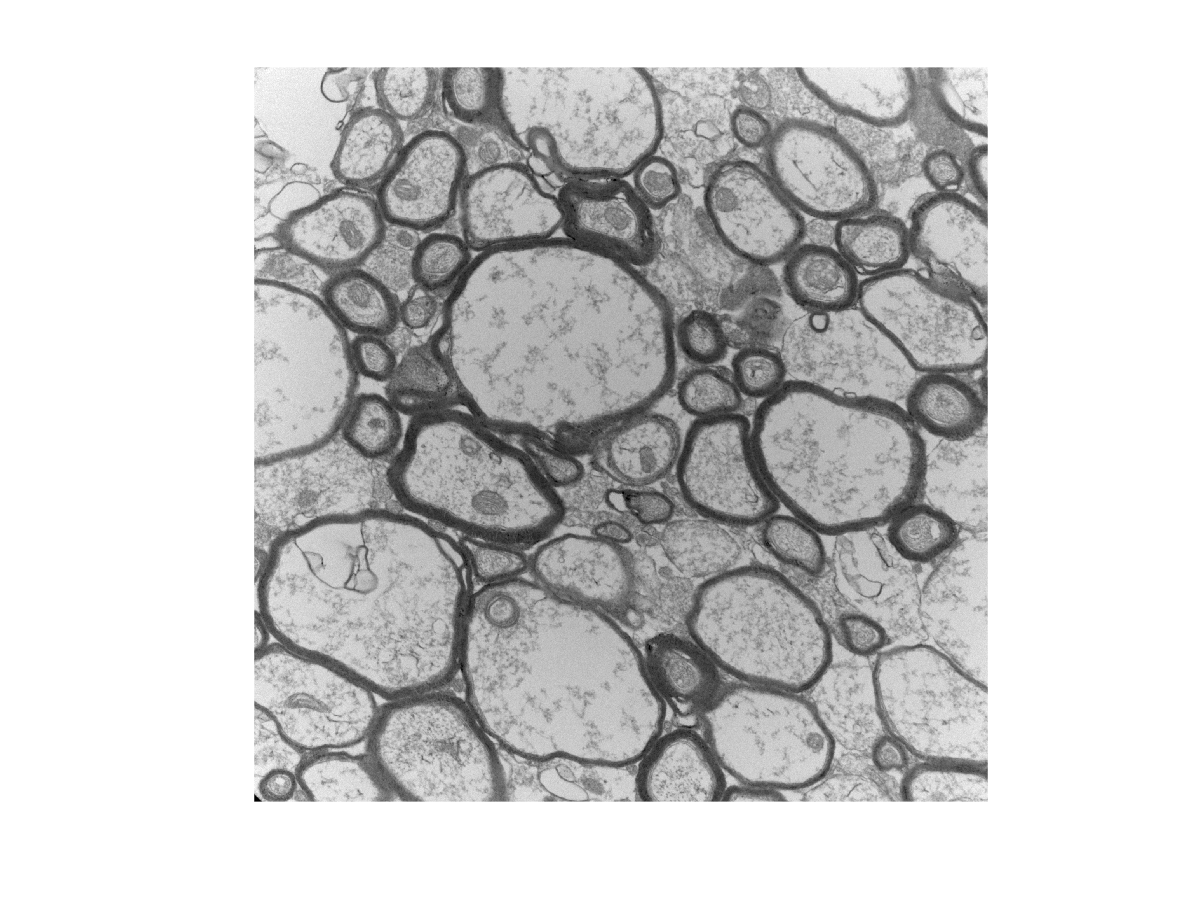

Supplement: Supplementary file 1 — Supplementary material [file mmc1.zip › Histology/Control_4_GCC_5.tif]

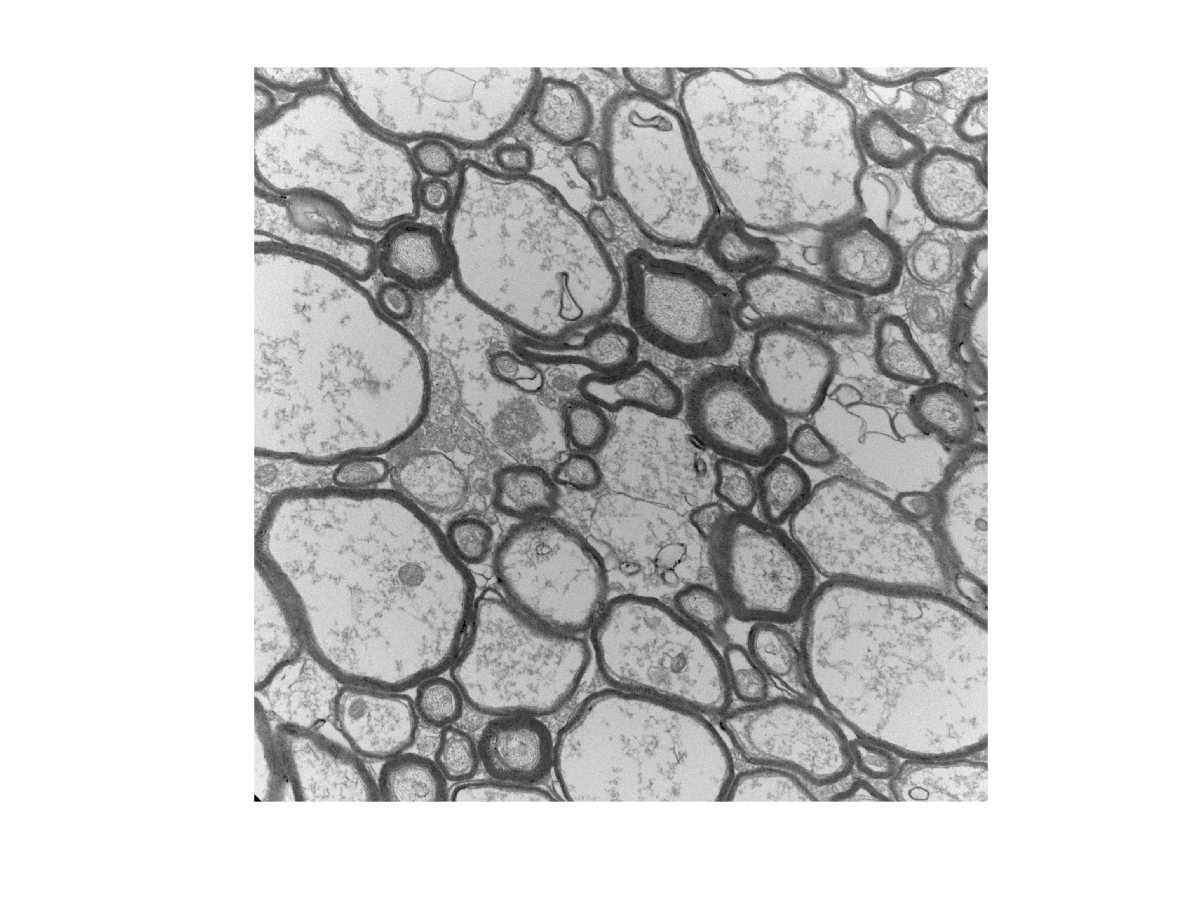

Supplement: Supplementary file 1 — Supplementary material [file mmc1.zip › Histology/Control_4_GCC_6.tif]

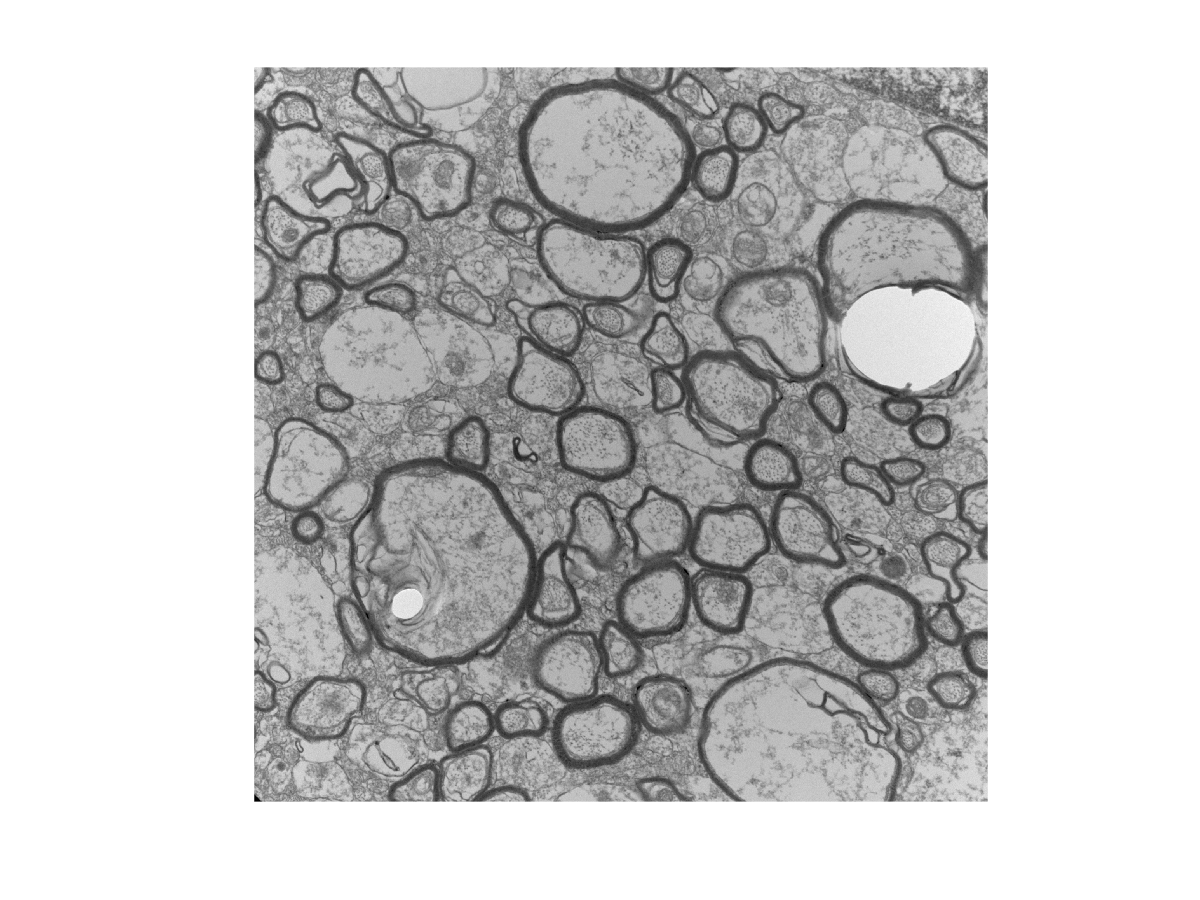

Supplement: Supplementary file 1 — Supplementary material [file mmc1.zip › Histology/Control_4_MidCC_1.tif]

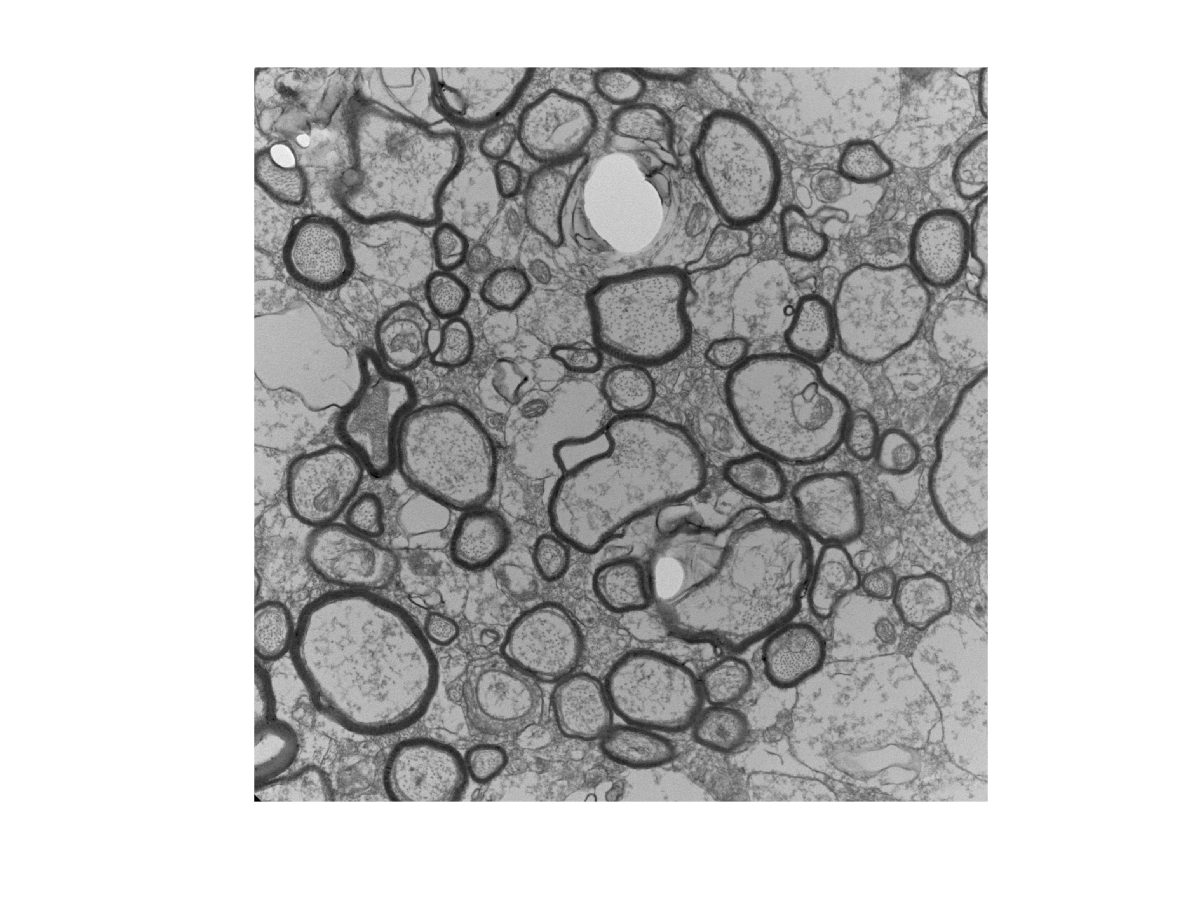

Supplement: Supplementary file 1 — Supplementary material [file mmc1.zip › Histology/Control_4_MidCC_2.tif]

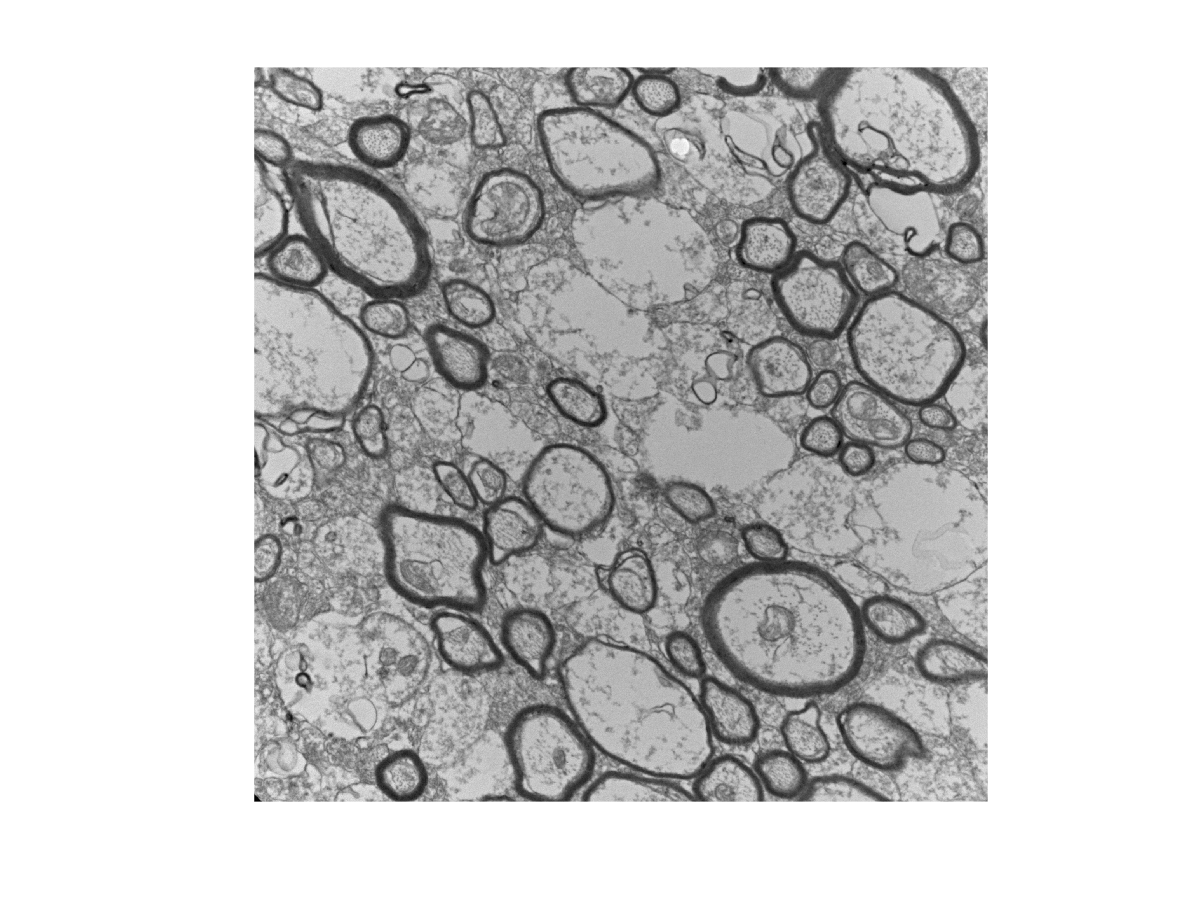

Supplement: Supplementary file 1 — Supplementary material [file mmc1.zip › Histology/Control_4_MidCC_3.tif]

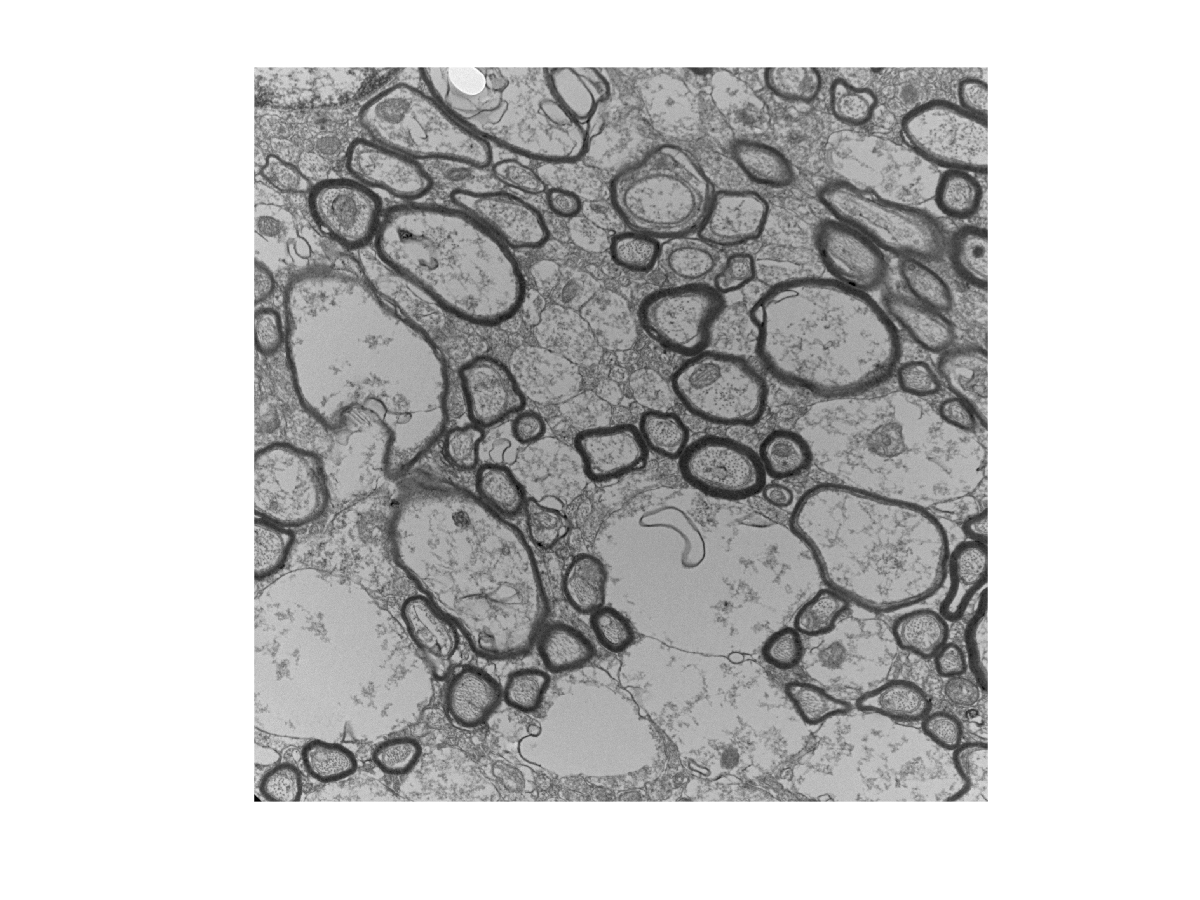

Supplement: Supplementary file 1 — Supplementary material [file mmc1.zip › Histology/Control_4_MidCC_4.tif]

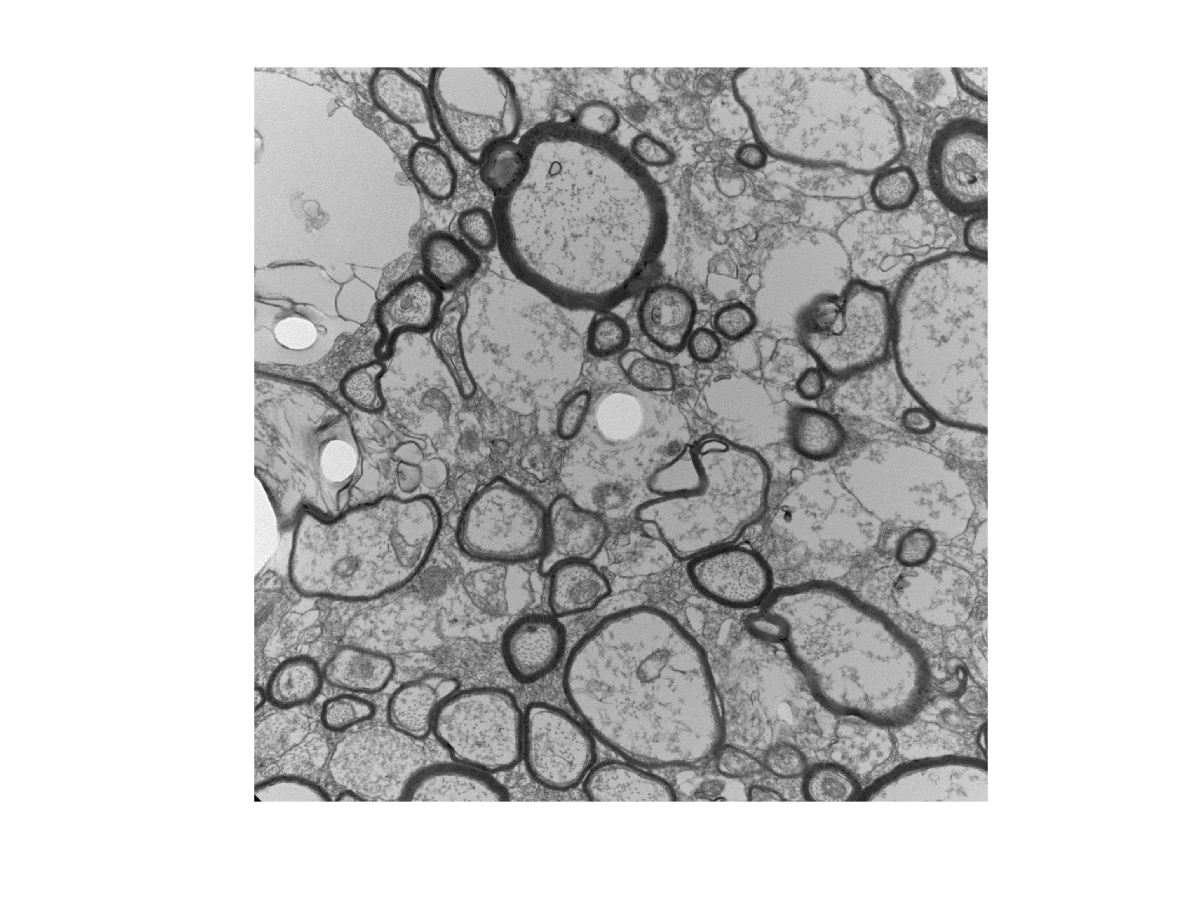

Supplement: Supplementary file 1 — Supplementary material [file mmc1.zip › Histology/Control_4_MidCC_5.tif]

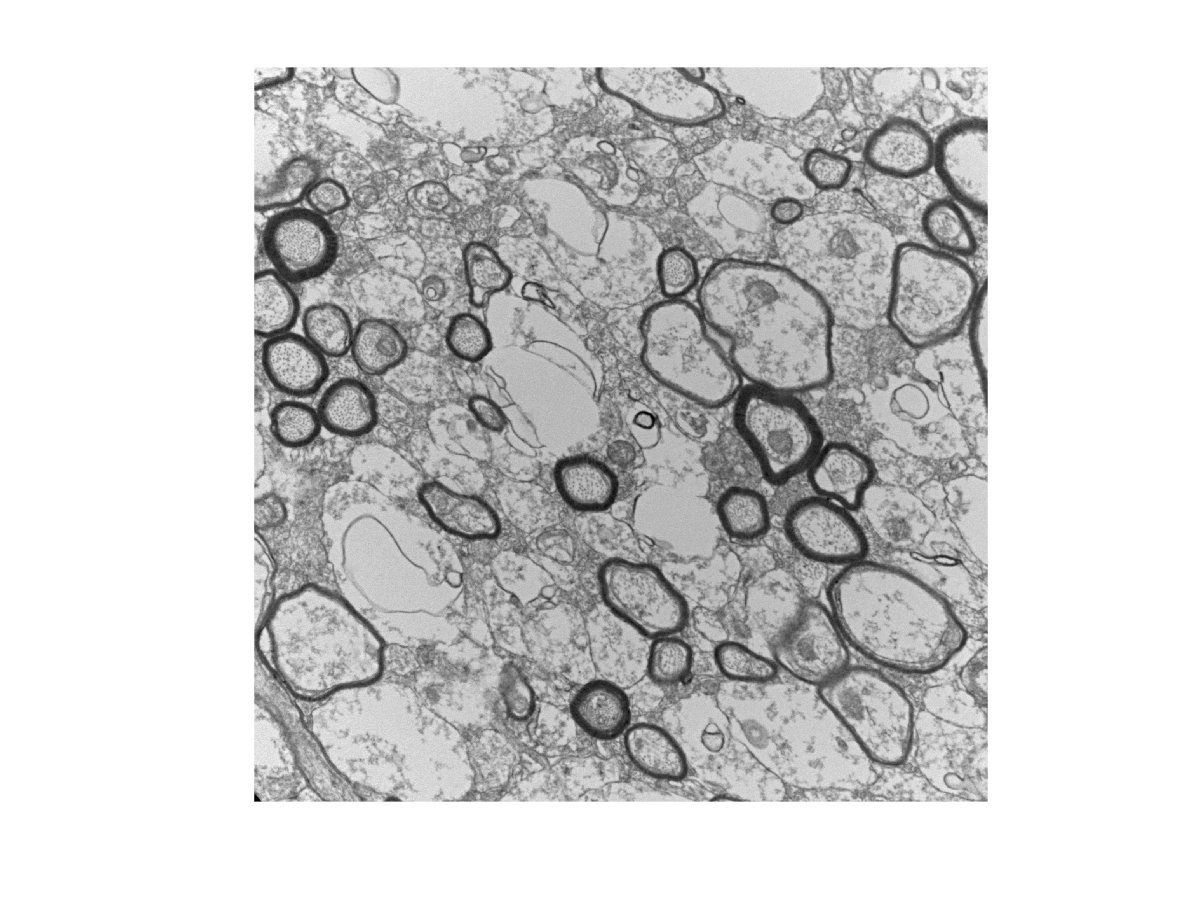

Supplement: Supplementary file 1 — Supplementary material [file mmc1.zip › Histology/Control_4_MidCC_6.tif]
